# Polyarylether‐Based 2D Covalent‐Organic Frameworks with In‐Plane D–A Structures and Tunable Energy Levels for Energy Storage

**DOI:** 10.1002/advs.202104898

**Published:** 2021-12-26

**Authors:** Nana Li, Kaiyue Jiang, Fermín Rodríguez‐Hernández, Haiyan Mao, Sheng Han, Xiaobin Fu, Jichao Zhang, Chongqing Yang, Changchun Ke, Xiaodong Zhuang

**Affiliations:** ^1^ School of Chemistry and Chemical Engineering Shihezi University Shihezi Xinjiang 832003 China; ^2^ The Meso‐Entropy Matter Lab School of Chemistry and Chemical Engineering State Key Laboratory of Metal Matrix Composites Shanghai Key Laboratory of Electrical Insulation and Thermal Aging Frontiers Science Center for Transformative Molecules Shanghai Jiao Tong University Shanghai 200240 China; ^3^ College of Chemistry and Molecular Engineering Zhengzhou University Zhengzhou Henan 450001 China; ^4^ Departamento de Química Módulo 13, Universidad Autónoma de Madrid Madrid 28049 Spain; ^5^ Department of Chemical and Biomolecular Engineering University of California Berkeley CA 94720 USA; ^6^ School of Chemical and Environmental Engineering Shanghai Institute of Technology Shanghai 201418 China; ^7^ Department of Molten Salt Chemistry and Engineering Shanghai Institute of Applied Physics Chinese Academy of Sciences Shanghai 201800 China; ^8^ Shanghai Synchrotron Radiation Facility Zhangjiang Laboratory Shanghai Advanced Research Institute Chinese Academy of Sciences Shanghai 201204 China; ^9^ Institute of Fuel Cells School of Mechanical Engineering Shanghai Jiao Tong University Shanghai 200240 China

**Keywords:** band gap, covalent‐organic frameworks, hexadecafluorophthalocyanine, micro‐supercapacitors, polyarylether

## Abstract

The robust fully conjugated covalent organic frameworks (COFs) are emerging as a novel type of semi‐conductive COFs for optoelectronic and energy devices due to their controllable architectures and easily tunable the highest occupied molecular orbital (HOMO) and the lowest occupied molecular orbital (LUMO) levels. However, the carrier mobility of such materials is still beyond requirements due to limited *π*‐conjugation. In this study, a series of new polyarylether‐based COFs are rationally synthesized via a direct reaction between hexadecafluorophthalocyanine (electron acceptor) and octahydroxyphthalocyanine (electron donor). These COFs have typical crystalline layered structures, narrow band gaps as low as ≈0.65 eV and ultra‐low resistance (1.31 × 10^−6^ S cm^−1^). Such COFs can be composed of two different metal‐sites and contribute improved carrier mobility via layer‐altered staking mode according to density functional theory calculation. Due to the narrow pore size of 1.4 nm and promising conductivity, such COFs and electrochemically exfoliated graphene based free‐standing films are fabricated for in‐plane micro‐supercapacitors, which demonstrate excellent volumetric capacitances (28.1 F cm^−3^) and excellent stability of 10 000 charge–discharge cycling in acidic electrolyte. This study provides a new approach toward dioxin‐linked COFs with donor‐acceptor structure and easily tunable energy levels for versatile energy storage and optoelectronic devices.

## Graphical abstract

A series of polyarylether‐based 2D covalent‐organic frameworks with in‐plane donor–acceptor structure and tunable energy levels are rationally synthesized. These materials show possible AA stacking with conduction band and also show excellent volumetric capacitance of up to 28.1 F cm^−3^.

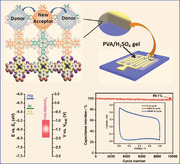

## 1 Introduction

Covalent‐organic frameworks (COFs) are a novel class of crystalline polymers with a variety of topological structures, intrinsic porosity and incredible functions.^[^ ^1^ ^]^ Among them, 2D fully conjugated COFs, with well‐defined layered structures, both in‐plane and out‐of‐plane conjugations and functional pores,^[^ ^2^ ^]^ have emerged as ideal scaffolds for light emitters,^[^ ^3^ ^]^ proton conductions,^[^ ^4^ ^]^ semiconductors,^[^ ^5^ ^]^ gas adsorption and separation,^[^ ^6^ ^]^ heterogeneous catalysis,^[^ ^7^ ^]^ energy conversion and storage,^[^ ^8^ ^]^ and so on. Linkages such as, B—O,^[^ ^1^, ^9^ ^]^ C—N,^[^ ^10^ ^]^ C═N,^[^ ^11^ ^]^ C═C,^[^ ^12^ ^]^ B═N,^[^ ^13^ ^]^ N—N,^[^ ^14^ ^]^ have been effectively established so for synthesis various types of 2D COFs. However, most of these bonds are single bonds to link different building blocks, limiting the *π*‐electron delocalization in‐plane and out‐of‐plane. In 2018, Yaghi and co‐workers^[^ ^15^ ^]^ introduced irreversible heteroaromatic dioxin linkage by nucleophilic aromatic substitution (S_N_Ar) reactions into COFs. The polyaromatic structure of these COFs benefits their excellent chemical stability,^[^ ^8a^ ^]^ functionability,^[^ ^15^, ^16^ ^]^ promising carrier mobility and/or electrical conductivity and electrochemical catalytic properties.^[^ ^8^, ^17^ ^]^ However, such polyarylether‐based 2D COFs (PAE‐2D COFs) can only be synthesized from very limited F‐contained building blocks and remain challenge for topological structure and energy level controlling.

Phthalocyanine (Pc) is a fascinating planar *π*‐building blocks with broad absorption profiles that can serve as intriguing units for construction of 2D frameworks.^[^ ^18^ ^]^ However, most reports only use octa‐hydroxyphthalocyanine (PcOH_8_) as a building block to synthesize 2D COFs because of its electron‐donor property and photo‐sensitiveness.^[^ ^19^ ^]^ Fluorinated phthalocyanine currently is also of great interest because of their remarkable electron transition properties: electron with fluorine peripheral substituents slightly irritates molecular orbitals that causing a little shift at the fluorescence and absorbance peaks, and enhanced tendency toward aggregation. Moreover, increased dipole moment at carbon bonds and the electron‐deficient nature for fused benzenes make such molecule a perfect substrate for attacking by S_N_Ar.^[^ ^20^ ^]^ Among these, hexadecafluorophthalocyanine (PcF_16_) displays charge convey and *π*‐electron delocalization according to the typical the lowest occupied molecular orbital (LUMO) and the highest occupied molecular orbital (HOMO) levels. The higher HOMO and LUMO energy levels of PcF_16_ also indicate that it has strong electron‐deficient property.^[^ ^21^ ^]^ Metal‐N_4_ coordination centers commonly exhibit reversible electrochemical redox couples, which contribute to the pseudocapacitance.^[^ ^22^ ^]^ For many years, Pc has been added to the carbon/carbon‐based materials to improve capacitive behavior.^[^ ^22b^ ^]^ Neena and colleagues reported that nickel phthalocyanine with reduced graphene oxide (GO) could have excellent electrical conductivity and high capacitance.^[^ ^23^ ^]^ However, because Pc is poorly soluble in most solvents, it is extremely difficult to precisely control the molecular‐level distribution of Pc in composite materials.^[^ ^24^ ^]^ Due to their Pc‐enriched structure, promising conductivity and outstanding stability, Pc‐based 2D metal–organic frameworks (MOFs) with strong layered interactions have lately shown tremendous potential in supercapacitors.^[^ ^25^ ^]^ But the development of 2D conjugated COFs with both rich Pc‐based building blocks and robust layered interactions remains great challenge.

Herein, PcF_16_, a type of fluorine‐rich building block, is used to synthesize new PAE‐based 2D COFs. These COFs are prepared by direct reaction between PcF_16_ and PcOH_8_ under solvothermal condition. Narrow micropore size (1.4 nm) was easily achieved. Various metal‐based PcF_16_ (denoted as MPc, M = Ni, Cu, Zn) were used to synthesize COFs, which exhibit low band gap down to ≈0.65 eV and ultra‐low resistance (1.31 × 10^−6^ S cm^−1^). Furthermore, these COFs also exhibit high chemical stability in concentrated acid and base media, which makes such PAE‐type COFs as appealing material platform for energy storage in harsh chemical environments. COF‐based free‐standing films with good conductivity can be easily fabricated after compositing with exfoliated graphene (EG). Using a direct laser scribing technique, interdigital microsupercapacitors (MSCs) were readily fabricated as binder‐free thin films and they exhibit excellent volumetric capacitances (28.1 F cm^−3^) as well as outstanding stability during 10 000 charge‐discharge cycles in acidic electrolyte. The fluorine‐rich building blocks for rational PAE‐based 2D COFs syntheses are presented in this study, paving the way for the development of high‐performance energy‐storage devices.

## 2 Results and Discussion

**Figure 1:**
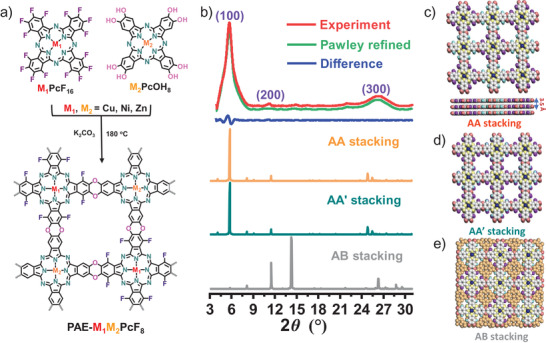
Synthesis of PAE‐M_1_M_2_PcF_8_ (M_1_, M_2_ = Ni, Cu, Zn) with dioxin linkages. a) Schematic illustration of nucleophilic aromatic substitution (S_N_Ar) reaction between M_1_PcOH_8_ and M_2_PcF_16_ to form PAE‐M_1_M_2_PcF_8_; b) PXRD patterns of PAE‐NiNiPcF_8_ (red curve, experimental result; green curve, Pawley refined pattern; blue curve, difference; orange curve, AA stacking; navy blue curve, AA′ stacking; gray curve, AB stacking); c) top view (top) and side view (bottom) of the AA stacking model; top view of d) the AA′ stacking and e) the AB stacking model.

In order to ascertain the feasibility of generating these PAE‐2D COFs, a dioxin‐linked molecular analog was prepared by S_N_Ar between the 1,2,3,4‐tetrafluoro benzene (TFB) and catechol building units in presence of potassium carbonate (K_2_CO_3_). When TFB was subjected to 1 equiv. of catechol, the proton of catechol and fluorine at position 2,3 of TFB are eliminated to generate dioxin‐linkage product, 4‐difluoro‐dibenzo‐1,4‐dioxin (1, see model reaction in Supporting Information). Because of the strong electron withdrawing fluoride atoms at positions 1 and 4, the electrophilicity of C—F bonds at positions 2 and 3 of TFB are expected to promote, which determines their high reactivity with catechol.^[^ ^8^, ^15^, ^16^, ^26^ ^]^ On the basis of this model reaction, the dioxin‐linked Pc‐based PAE‐type COFs (denoted as PAE‐M_1_M_2_PcF_8_) were synthesized through S_N_Ar reaction between extended square building block of hexadecafluoro metal‐phthalocyanine (M_1_PcF_16_) and octahydroxy metal‐phthalocyanine (M_2_PcOH_8_) in presence of K_2_CO_3_ in dimethylacetamide (**Figure** **1**a). The synthesis and characterizations of monomers (M_1_PcF_16_ and M_2_PcOH_8_) are displayed in supporting information (Figures S1–S15, Supporting Information). Not only this method offers a F‐rich building block toward PAE‐based COFs with in‐plane corrected (D–A) structure, but also paves the way for possible D–A structure out‐of‐plane in the future.^[^ ^27^ ^]^

### 2.1 Crystalline Structure

**Figure 2:**
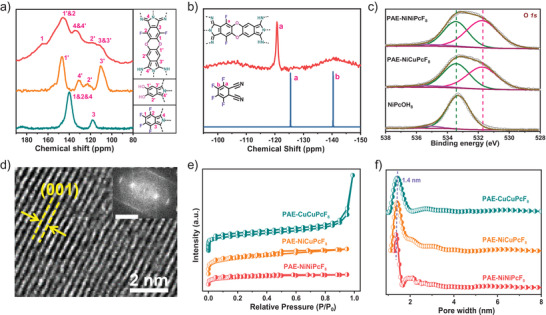
a) Solid‐state ^13^C NMR spectra of PAE‐NiNiPcF_8_, NiPcOH_8_, and NiPcF_16_, respectively; b) solid‐state ^19^F NMR spectrum of PAE‐NiNiPcF_8_ and the ^19^F NMR spectrum of TFP in DMSO‐d_6_; c) high‐resolution O1s XPS spectra of PAE‐NiNiPcF_8_, PAE‐NiCuPcF_8_, and NiPcOH_8_; d) HRTEM image and lattice fringes of PAE‐CuCuPcF_8_ (inset: fast Fourier transform analysis; scale bar: 5 nm^−1^); e) N_2_ physisorption isotherms and f) pore size distributions of PAE‐NiNiPcF_8_, PAE‐CuCuPcF_8_, and PAE‐NiCuPcF_8_.

The crystalline structures of PAE‐based 2D COFs were analyzed by powder X‐ray diffraction (PXRD) in combination with theoretical structural simulations (Figure 1b–e and Figure S16, Supporting Information). The PXRD of PAE‐NiNiPcF_8_ exhibited crystalline nature with a series of peaks at 5.7°, 11.0°, and 26.1°, attributing to (100), (200), and (001) facets, respectively, which indicates long‐range ordering structure of the ab‐plane with center‐to‐center distance of 15.4 Å. The broad peak at 26.1° is consistent to an interlayer distance and manifests the poor coherence of stacking‐layer in the out‐of‐plane direction (Figure 1b, red curve).^[^ ^25a^ ^]^ Several tetragonal skeletons with different interlayer arrangements were optimized to study the lattice information (Figure 1c–e). Fully eclipsed AA‐stacking model (Figure 1c) and staggered AA′‐stacking (Figure 1d) model with offset distances show lattice parameters *α* = *β* = *γ* = 90°, *a* = *b* = 15.4 Å, and *c* = 3.5 Å and give PXRD patterns (Figure 1b), consistent with the experimental results. Furthermore, the Pawley refinement duplicated concrete PXRD profile is carried out with Rwp = 5.92% and Rp = 4.33% (**Figure** **2**b, green curve), which is in good agreement with the experimental pattern. Additionally, the alternative staggered 2D arrangement called AB stacking model also be considered, in which these adjacent layers are offset horizontally by *a*/2 and *b*/2 distances (Figure 1b, gray line and Figure 1e). The simulated PXRD pattern of AB stacking showed significant deviations comparing the experimental data, ruling out the AB stacking model. According to relative stability theory calculations of layered PAE‐NiNiPcF_8_ in different stacking modes, the AA geometry was energetically most favored (Table S1, Supporting Information). On the basis of these results, PAE‐NiNiPcF_8_ is proposed to have the AA stacking architecture with square cell (*a* = *b* = 15.4 Å, *c* = 3.5 Å), which may result from the strong tendency of aromatic groups between adjacent sheets to generate coplanar aggregates.^[^ ^16^ ^]^

The solid‐state ^13^C cross‐polarization magic‐angle‐spinning nuclear magnetic resonance spectroscopy (CP/MAS NMR), solid‐state ^19^F MAS NMR spectroscopy and Fourier transform infrared spectroscopy (FTIR) were devoted to explore the chemical bond of PAE‐type COFs. The ^13^C CP/MAS NMR spectrum of PAE‐NiNiPcF_8_ shows resonance signals at 146.2 and 165.5 ppm, which are characteristic of carbons from NiPcOH_8_ at position 1′ and NiPcF_16_ at position 1, respectively (Figure 2a).^[^ ^8^, ^15^, ^28^ ^]^ The signals at *δ* = 110.7, 122.5, and 133.6 ppm can be identified to these carbons on the Pc and phenyl groups. Furthermore, the ^19^F MAS NMR spectrum of PAE‐NiNiPcF_8_ shows only one distinct peak at −120.79 ppm (Figure 2b), which is different from the starting material of two kinds of fluorine (−125.45 and −140.39 ppm). The slightly deviation fluorine position of PAE‐NiNiPcF_8_ compared with tetrafluorophthalonitrile (TFP) should originate from the orthogonal fluorine (−140.39 ppm) in TFP.^[^ ^29^ ^]^ Similarly, the ^13^C CP‐MAS spectrum of PAE‐NiCuPcF_8_ can also be similarly assigned (Figure S17, Supporting Information). Besides, the FTIR spectrum of PAE‐NiNiPcF_8_ shows vibration bands at ≈1256 and ≈1048 cm^−1^, belonging to the characteristic asymmetric and symmetric stretching modes of dioxin C—O, respectively, which indicates the formation of PAE linkages (Figure S18, Supporting Information).^[^ ^15^, ^16^ ^]^ All these results confirm that the dioxin‐linked PAE‐type 2D COFs are successfully synthesized by S_N_Ar reaction.

The X‐ray photoelectron spectroscopy (XPS) analysis was estimated to determine the surface chemical states and elemental composition of these PAE‐based COFs (Figure 2c and Figures S19–S21, Supporting Information). The O 1s spectra of PAE‐NiNiPcF_8_, PAE‐NiCuPcF_8_ and NiPcOH_8_ are shown in Figure 2c. Deconvolution of the O 1s signals of PAE‐NiNiPcF_8_ and PAE‐NiCuPcF_8_ generated three kinds of peaks at ≈531.7, ≈533.5, and ≈535.4 eV, which should be attributed to the C—O—C bond of dioxin linkages, C—O—H bond and chemisorbed oxygen, respectively.^[^ ^30^ ^]^ Compared with NiPcOH_8_, the peak of C—O—H functional group significantly decreased and a new peak at 531.7 eV dominants in PAE‐NiNiPcF_8_ and PAE‐NiCuPcF_8_, suggesting the presence of C—O—C linkage. These results are accordance with those results of FTIR analysis, further confirming the formation of polyarylether linkages. Moreover, the Ni 2p and Cu 2p XPS spectra exhibit two groups of characteristic peaks in 854.58 and 872.05 eV for Ni 2p_3/2_ and Ni 2p_1/2_, 934.79 and 954.77 eV for Cu 2p_3/2_ and Cu 2p_1/2_, respectively, suggesting stable existence of Ni (II) and Cu (II) species in PAE‐NiNiPcF_8_, PAE‐CuCuPcF_8_, and PAE‐NiCuPcF_8_ (Figures S19–S21 and Table S3, Supporting Information).^[^ ^31^ ^]^ To further elaborate the chemical states of the Ni and Cu atoms, X‐ray absorption spectroscopy and extended X‐ray absorption fine structure (EXAFS) analyses were conducted (Figures S22–S25, Supporting Information). The Ni K‐edge X‐ray absorption near‐edge structure (XANES) spectrum of PAE‐NiNiPcF_8_ (Figure S22a, Supporting Information) exhibits a typical Ni (II) peak at 8340 eV, which is similar to reported NiPc, consisting with the XPS results. The EXAFS spectrum of PAE‐NiNiPcF_8_ (Figure S22b, Supporting Information) displays the radial structure functions and clearly demonstrates the characteristic Ni—N coordination in PAE‐NiNiPcF_8_ with intensive peak at ≈1.4 Å.^[^ ^32^ ^]^ The Cu K‐edge XANES spectrum of PAE‐CuCuPcF_8_ (Figure S23a, Supporting Information) shows a typical Cu peak at 8985 eV (1s to 3d electron transition), indicating the oxidation valence of Cu atom as +2. The EXAFS spectrum of PAE‐CuCuPcF_8_ (Figure S23b, Supporting Information) reveals the existence of Cu—N coordination in PAE‐CuCuPcF_8_ with intensive peak at ≈1.5 Å, which is in agreement with control sample CuPc.^[^ ^33^ ^]^ All these results provide solid proof for chemical states and coordination environment of as‐synthesized COFs.

The high resolution transmission electron microscopy (HRTEM) was performed to visualized analysis the sample morphology of PAE‐2D COF. The TEM image of PAE‐CuCuPcF_8_ (Figure 2d) displays the distinct lattice fringes of (001) crystal plane corresponding to an intralayer distance of 3.5 Å, which is suitable to the simulated crystal structure in Figure 1c. The surface areas and porosity of these samples were evaluated by nitrogen sorption measurements at 77.3 K. As shown in Figure 2e and Figure S26, Supporting Information, the N_2_ isotherms of these COFs are found to have characteristic type‐I shape, indicating micro‐sized (<2.0 nm) porosity. The Brunauer–Emmett–Teller (BET) surface areas of PAE‐NiNiPcF_8_, PAE‐CuCuPcF_8_, and PAE‐NiCuPcF_8_ were measured to be 230, 264, 369 m^2^ g^−1^, respectively. Besides, the pore size distributions of these COFs were calculated using a quenched solid functional theory model to fit the adsorption isotherms, yielding pore widths centered at ≈1.40 nm, which agrees well with the XRD results (Figure 2f). The chemical stability of PAE‐2D COFs also be surveyed by immersing PAE‐NiNiPcF_8_ into extreme chemical conditions, including concentrated H_2_SO_4_ (12 m), HCl (12 m), and KOH (12 m) at room temperature for over 24 h. After treatment in those solutions, PAE‐NiNiPcF_8_ was collected by filtration and rinsed thoroughly with water and tetrahydrofuran (The sample is abbreviated as PAE‐NiNiPcF_8_‐CST). After treatment, the crystallinity of PAE‐NiNiPcF_8_ can be maintained (Figure S27, Supporting Information). The SEM image of PAE‐NiNiPcF8‐CST shows sheet morphology with sizes from 5 to 20 µm (Figure S28, Supporting Information). The BET surface area of PAE‐NiNiPcF_8_‐CST also retained well as ≈224 m^2^ g^−1^ (Figure S29, Supporting Information). The FTIR spectrum of PAE‐NiNiPcF_8_‐CST displays obvious asymmetric and symmetric stretching peak of C—O—C at ≈1256 and ≈1048 cm^−1^, respectively (Figure S30, Supporting Information). Thermogravimetric analysis （TGA） result indicates that PAE‐NiNiPcF_8_‐CST is stable up to 400 °C (Figure S31, Supporting Information). Such high chemical stability can be ascribed to the robust dioxin linkage. All these results indicate good stability of as‐prepared COFs.

### 2.2 Optical and Electronic Structure Characterization

**Figure 3:**
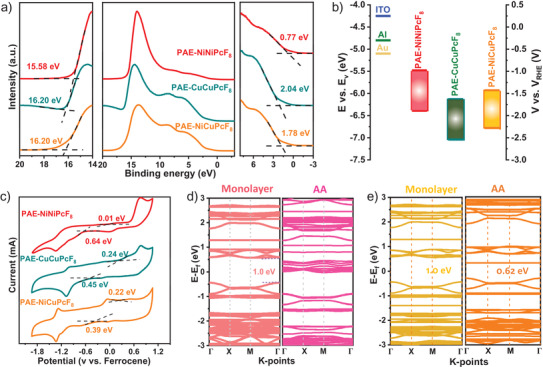
a) The UPS spectra and b) CB and VB of PAE‐NiNiPcF_8_, PAE‐CuCuPcF_8_, and PAE‐NiCuPcF_8_; c) CV profiles of PAE‐NiNiPcF_8_, PAE‐CuCuPcF_8_, and PAE‐NiCuPcF_8_ measured in CH_3_CN solution at 20 mV s^−1^; the calculated band structures of monolayer and AA stacking for d) PAE‐NiNiPcF_8_ and e) PAE‐NiCuPcF_8_.

The ultraviolet photoelectron spectroscopy (UPS) was studied to detect energy levels of valence band (*E*_vb_) of these COFs. The *E*_vb_ of PAE‐NiNiPcF_8_ was determined as 6.39 eV by subtracting the width of UPS using excitation photon energy (HeI, 21.22 eV), which is lower than the *E*_vb_ of PAE‐CuCuPcF_8_ (7.04 eV) and PAE‐NiCuPcF_8_ (6.78 eV) (**Figure** **3**a). Furthermore, the optical bandgap ($E_{g}^{\mathrm{opt}}$) of PAE‐NiNiPcF_8_, PAE‐CuCuPcF_8_, and NiCuPcF_8_‐COF could be estimated from the absorption edge of the UV absorption spectra, in which the *T*_auc_ plots reveal the $E_{g}^{\mathrm{opt}}$ of ≈0.91, ≈0.91, ≈0.85 eV, respectively (Figures S32–S35, Supporting Information). Then, the conduction band (*E*_cb_) can be measured according to $E_{g}^{\mathrm{opt}}$. The summarized *E*_vb_ and *E*_cb_ of these COFs are shown in Figure 3b in comparison with the work functions of ITO, Al, and Au. The narrow band gaps ≈0.90 eV demonstrate the semiconductive nature of the prepared samples. Besides, the cyclic voltammetry (CV) of PAE‐based 2D COFs were analyzed in nitrogen‐saturated CH_3_CN solution to investigate electrochemical bandgap ($E_{g}^{\mathrm{elec}}$). According to the onset reduction and oxidation potentials (vs ferroncene), the energy level of *E*_vb_ and $E_{g}^{\mathrm{elec}}$ can be calculated to be 4.81 and 0.65 eV for PAE‐NiNiPcF_8_, 5.04 and 0.69 eV for PAE‐CuCuPcF_8_, 5.02 and 0.61 eV for PAE‐NiCuPcF_8_, which are consistent with optical band gaps and UPS results (Figure 3c and Table S4, Supporting Information).^[^ ^25c^ ^]^

To better understand the effect of different metal‐centered and stacking models on energy band, density functional theory (DFT), including the energy band structures and projected density of states (PDOS) were employed to understand the energy bands of PAE‐NiNiPcF_8_, PAE‐CuCuPcF_8_ and PAE‐NiCuPcF_8_ (Figure 3d,e; Figures S37 and S38, Supporting Information). Figure S37, Supporting Information, reveals the conduction band (CB) and valence band (VB) with nearly dispersionless of these PAE‐based COFs, which means that in‐plane charge transport of monolayer was virtually null due to the extraordinarily large effective mass of charge carriers.^[^ ^25c^ ^]^ Meanwhile, the similar band structure indicates independent of metal center substitution in‐plane. Notably, for the AA stacking model, the band gaps of PAE‐NiNiPcF_8_ and PAE‐NiCuPcF_8_ become narrowing down to 0 and 0.62 eV compared with that of monolayer (≈1.0 eV) owing to the enhancement of *π*–*π* interaction between layers (Figure 3d,e).^[^ ^25c^ ^]^ This survey on these PAE‐based COFs also shows that proximity effect has a significant effect on the band gap and electronic structure of framework materials.^[^ ^34^ ^]^ The underlying reason of PAE‐NiNiPcF_8_ and PAE‐NiCuPcF_8_ for the energy level difference can be attributed to NiPc shows much smaller resonance energy with its neighboring units and therefore decreases the energy barrier of out‐of‐plane charge transfer transition.^[^ ^35^ ^]^ Furthermore, the PDOS plots (Figure S38, Supporting Information) of PAE‐NiNiPcF_8_ and PAE‐NiCuPcF_8_ indicate that both C and N contribute to CB minimum, whereas the VB maximum is dominated by the C and O atoms, suggesting Pc units mainly contribute to the CB, while PAE units dominant the VB. Besides, the AA stacking exhibits typical higher electron states near a Fermi level than monolayer in according with highly dispersive of band, thereby benefitting to the band‐like transportation of charge carrier.^[^ ^36^ ^]^

### 2.3 PAE‐M_1_M_2_PcF_8_ as Electrodes for MSCs

**Figure 4:**
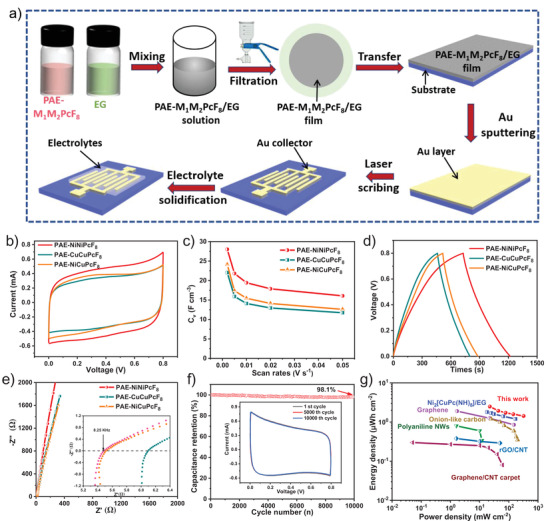
The MSCs behavior of PAE‐M_1_M_2_PcF_8_. a) Schematic fabrication of PAE‐M_1_M_2_PcF_8_ based MSCs; b) CV curves at 50 mV s^−1^; c) specific volumetric capacitances calculated from CV curves at different scan rates; d) GCD curves at 0.04 mA cm^−2^; e) the impedance curves of the PAE‐NiNiPcF_8_, PAE‐CuCuPcF_8_, and PAE‐NiCuPcF_8_‐based MSCs (inset: magnified high‐frequency region); f) the cycling stability of PAE‐NiNiPcF_8_ for MSC at 50 mV s^−1^ (inset: CV curves at 1st, 5000th, and 10 000th cycle); g) Ragone plots of PAE‐NiNiPcF_8_ and reported carbon/graphene, conducting polymers and other reported framework materials for the MSCs.

The fabrication process for MSCs is schematically illustrated in **Figure** **4**a. First, the PAE‐M_1_M_2_PcF_8_/EG solution was prepared from the combination of PAE‐M_1_M_2_PcF_8_ and EG in N‐methyl‐2‐pyrrolidone at room temperature. Then, PAE‐M_1_M_2_PcF_8_/EG hybrid films were fabricated through vacuum filtration and transferred onto glass substrates. Subsequently, the as‐prepared films were deposited using Au and then interdigital electrodes were directly produce by laser‐scribed method. After that, gel electrolyte (H_2_SO_4_/PVA) was drop‐casted onto these electrodes and solidified overnight. The electrochemical behavior of MSCs based on these hybrid films were investigated first by electrochemical CV and galvanostatic charge–discharge (GCD) (Figure 4b–d and Figures S42–S48, Supporting Information). The integral area of CV curve for PAE‐NiNiPcF_8_ based MSC presented highest specific volumetric capacitance (*C*_v_) (28.1 F cm^−3^ at scan rate of 2 mV s^−1^) compared with other fabricated devices (22.0 F cm^−3^ for PAE‐CuCuPcF_8_ and 24.1 F cm^−3^ for PAE‐NiCuPcF_8_) (Figure 4b,c). Moreover, the CV profiles of PAE‐NiNiPcF_8_ displayed the approximately rectangular shape at different scanning rates from 2 to 50 mV s^−1^, arising from the well‐defined structure and high specific surface area of PAE‐NiNiPcF_8_ (Figure S43, Supporting Information).^[^ ^25a^ ^]^ The capacitive property of these COFs was further investigated through GCD measurement at various current densities ranging from 0.04 to 0.4 mA cm^−2^. PAE‐NiNiPcF_8_ exhibited the highest *C*_v_ of 24.5 F cm^−3^ at 0.04 mA cm^−2^ (Figure 4d), consistent with the result based on CV curves.

Furthermore, to better understand the processes that occur at electrode and surfaces, the electrochemical impedance spectra (EIS) of PAE‐NiNiPcF_8_, PAE‐CuCuPcF_8_, and PAE‐NiCuPcF_8_ were studied (Figure 4e, inset: magnified view of the high‐frequency region). The semicircle in the EIS derives from the combination of capacitance and resistance, whose intercept in the high‐frequency region contributes the solution resistance (*R*_s_).^[^ ^23^ ^]^ From the inset of Figure 4e, the *R*_s_ is found to be ≈5.48, ≈5.52, ≈6.10 Ω for PAE‐NiNiPcF_8_, PAE‐CuCuPcF_8_, and PAE‐NiCuPcF_8_, respectively. Besides, the absence of a prominent semicircle indicates that the charge transfer resistance between electrolyte and electrode surface is negligible, similar to carbon material.^[^ ^37^ ^]^ Furthermore, the Nyquist plots of PAE‐NiNiPcF_8_/EG electrode are closer to *Y*‐axis than other MSCs in the low‐frequency region, implying the ideal capacitive behavior.^[^ ^22a^ ^]^ The long‐term cycling stability of PAE‐NiNiPcF_8_ electrode was estimated by CV cycling measurement at 50 mV s^−1^ (Figure 4f). After 10 000 cycles, PAE‐NiNiPcF_8_ electrode possesses outstanding cycling stability over 98.1% of the initial capacitance performance. To illustrate the overall property of the PAE‐NiNiPcF_8_, Ragone plots were calculated in Figure 4g. Notably, PAE‐NiNiPcF_8_ MSCs deliver ultrahigh areal power density of 321.34 mW cm^−2^, which are higher than those of typical conducting MOF‐based MSCs,^[^ ^25a^ ^]^ carbon‐based MSCs,^[^ ^38^ ^]^ and graphene‐based MSCs^[^ ^39^ ^]^ (Table S5, Supporting Information). In addition, the pseudocapacitance (*C*_p_) and double layer capacitance (*C*_dl_) behavior contribution of PAE‐NiNiPcF_8_ was studied through Trasatti analysis (Figure S48, Supporting Information). The capacitance contribution rate of PAE‐NiNiPcF_8_ from *C*_p_ and *C*_dl_ were calculated to be 49.5% and 50.5% using the Trasatti method, respectively, which quantitatively analyzes the *C*_dl_ behavior of PAE‐NiNiPcF_8_.^[^ ^40^ ^]^

In order to check the energy storage performance of PAE‐NiNiPcF_8_‐CST, the electrochemical behavior was investigated by electrochemical CV and GCD in PVA/H_2_SO_4_ (Figure S49, Supporting Information). The integral area of CV curve for PAE‐NiNiPcF_8_‐CST‐based MSC exhibits excellent specific *C*_v_ of 27.8 F cm^−3^ at scan rate of 2 mV s^−1^, which is comparable to PAE‐NiNiPcF_8_ (28.1 F cm^−3^ at scan rate of 2 mV s^−1^, Figure S49a, Supporting Information). The capacitive property of PAE‐NiNiPcF_8_‐CST by GCD measurement exhibits that the *C*_v_ is 21.4, 20.3, 19.4, 16.3, 14.8 F cm^−3^ at 0.04, 0.06, 0.08, 0.16, 0.4 mA cm^−2^ (Figure S49b, Supporting Information), respectively, consistent with the result based on CV curves. Furthermore, the EIS curves of PAE‐NiNiPcF_8_‐CST were studied. From the inset of Figure S49d, Supporting Information, the *R*_s_ is found to be ≈7.50 Ω for PAE‐NiNiPcF_8_‐CST and the negligible semicircle shows that the charge transfer resistance is very small, indicating the good conductivity of PAE‐NiNiPcF_8_.

### 2.4 Charge Storage Mechanism

**Figure 5:**
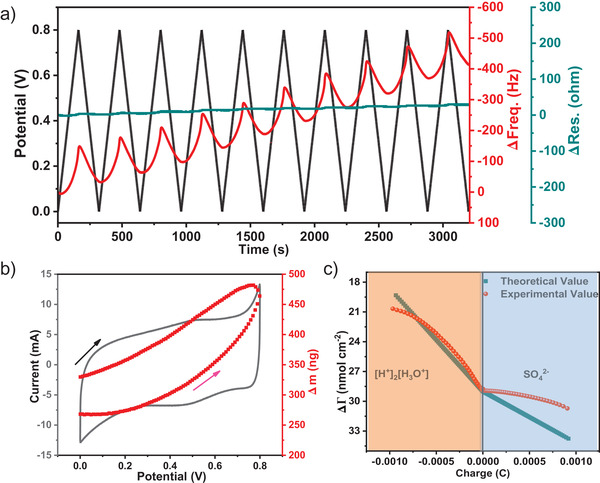
a) The responses of EQCM measures for PAE‐NiNiPcF_8_. The black curve relates to the potential‐time dependency relationship; the red curve indicates the response to the frequency; the blue curve is the resonance resistance changes; b) CV curve and corresponding Δ*m* of PAE‐NiNiPcF_8_ in H_2_SO_4_ electrolyte; c) the experimental and theoretical Δ*Γ* as a function of charge density (Δ*Q*) during charging–discharging processes.

Electrochemical quartz crystal microbalance (EQCM) test could serve as a kind of in situ experimental method for probing the charge storage mechanism of supercapacitors due to its ultra‐sensitive change of the thickness and gravimetry (10^−9^ g) in the charging–discharging process.^[^ ^41^ ^]^ The original EQCM data is showed in **Figure** **5**a. With the increase of positive potential, the quartz resonance frequency (∆f) decreases monotonously, indicating a monotonous increase of the electrode's mass during the charge process (charge accumulation process of the electrode materials), which should correspond to the adsorption of electrolyte ions.^[^ ^42^ ^]^ The resonance resistance (Δ*R*) of PAE‐NiNiPcF_8_ hardly changes during the electrochemical measurement (Figure 5a), so it is feasible to calculate the mass change (Δ*m*) of the electrode in electrochemical cycles by Sauerbrey equation.^[^ ^41^ ^]^ Meanwhile, the Δ*R* maintained constant means no change in viscosity during charging and discharging process, indicating that PAE‐NiNiPcF_8_ sample do not deform.^[^ ^41^ ^]^ The CV curve and Δ*m* of electrode material at 5 mV s^−1^ are described in Figure 5b. When the voltage increase, the electrode's mass continuously increases corresponding to the adsorption of cations and anions. In contrast, when the voltage decrease, the mass of electrode decreases indicating the desorption process of ions.^[^ ^43^ ^]^ The electrode mass after electrochemical cycling is slightly incremental compared to the initial value, due to the accumulation of anions and cationic.^[^ ^41^, ^44^ ^]^ Figure 5c exhibits the experimental and theoretical ion population changes (Δ*Γ*) as a function of charge (Q). In the stage of *Q* < 0, the Δ*Γ*_theor_ and Δ*Γ*_exp_ display a good fit when the *M*_i_ is equal to 19 g mol^−1^, which indicates H_3_O^+^ ions adsorbed on the electrode surface. In addition, in the stage of *Q* > 0, the SO_4_^2−^ ions will be adsorbed and exactly *M*_i_ of 96 g mol^−1^ is a good coincidence for Δ*Γ*_theor_ and Δ*Γ*_exp_. Besides, the increase behavior of Δ*Γ*_theor_ during *Q* > 0 may derive from the Faraday reaction of NiPc and SO_4_^2−^, in which SO_4_^2−^ adsorbed onto pyrrole N of NiPc, corresponding with the reported storage mechanism of MPc units.^[^ ^45^ ^]^

### 2.5 Quantum Capacitance Calculation

**Figure 6:**
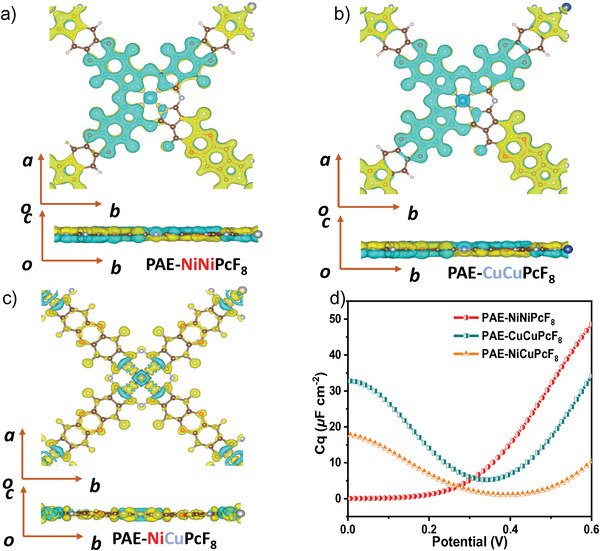
Differential charge density of a) PAE‐NiNiPcF_8_; b) PAE‐CuCuPcF_8_; c) PAE‐NiCuPcF_8_. Yellow and blue colors indicate charge accumulation and reduction, respectively; d) calculated potential dependent CQs for PAE‐NiNiPcF_8_, PAE‐CuCuPcF_8_, and PAE‐NiCuPcF_8_.

In addition, to reflect the charge gains/losses and bond properties between atoms, the differential charge density was calculated. **Figure** **6**a–c show the differential charge density distribution of PAE‐NiNiPcF_8_, PAE‐CuCuPcF_8_, and PAE‐NiCuPcF_8_. There is significant charge accumulation around polyarylether units and the charge loss around Pc bone, matching to PDOS results (Figure S34, Supporting Information). Combining with the above experimental and computational results, the charge‐storage mechanism of PAE‐NiNiPcF_8_ is summarized as the following: 1) when PAE‐NiNiPcF_8_ is negatively charged, the Faraday reaction occurs on nucleophilic centers (polyarylether units) with H_3_O^+^ adsorbed; 2) when PAE‐NiNiPcF_8_ is positively charged, the Faraday reaction occurs on electrophilic centers (NiPc units) with SO_4_^2−^ adsorbed onto pyrrole N of Pc.^[^ ^45^ ^]^ To explore the intrinsic capacitance, the quantum capacitances (CQs) of the electrode materials are further calculated.^[^ ^46^ ^]^ As shown in Figure 6d, the CQ of PAE‐NiNiPcF_8_ increases gradually and up to 48.7 µF cm^−2^ at 0.6 V with the increasing of potential. The CQ plots of PAE‐CuCuPcF_8_, PAE‐NiCuPcF_8_ present U‐shape and reduce to the minimum at 0.33 V. However, when their potential reach higher than 0.33 V, the CQs of all three electrodes increase sharply, indicating enhanced density of states under high voltage.^[^ ^46^, ^47^ ^]^

A series of PAE‐ZnZnPcF_8_, PAE‐NiZnPcF_8_, and PAE‐CuZnPcF_8_ COFs based on the same strategy of PAE‐NiNiPcF_8_ have also been synthesized to verify the method‐versatility of PAE‐2D COFs with tunable metal centers in MSCs. The PAE‐ZnZnPcF_8_, PAE‐NiZnPcF_8_, and PAE‐CuZnPcF_8_ have the same morphology and crystalline structure as that of PAE‐NiNiPcF_8_ (Figures S50–S59, Supporting Information). The supercapacitive behavior of PAE‐ZnZnPcF_8_, PAE‐NiZnPcF_8_, and PAE‐CuZnPcF_8_ were also evaluated (Figure S60–S62, Supporting Information). PAE‐ZnZnPcF_8_, PAE‐NiZnPcF_8_, and PAE‐CuZnPcF_8_ exhibit the excellent specific areal capacitance (*C*_A_) of 23.2, 21.0, 21.6 mF cm^−2^ at 0.04 mA cm^−2^, respectively. The study on above unprecedented experimental and theoretical calculation demonstrates such new PAE‐2D COFs have remarkable practical applications in versatile fields.

## 3 Conclusion

In summary, a kind of fluorine‐rich building blocks (PcF_16_) are applied to synthesize a series of tunable metal‐center PAE‐2D COFs by direct reaction between PcF_16_ and PcOH_8_, which exhibits excellent crystallinity and higher stability even under concentrated acids and base. Owing to highly ordered crystalline molecular framework and periodic Pc‐on‐Pc columns, these PAE‐based 2D COFs can afford transmission pathways for charge carrier. According to DFT simulation and experiments, the prepared PAE‐NiNiPcF_8_ based on a high degree of conjugation and periodic structure exhibit narrow band gap and low resistance. Besides, these COF assembling with electrochemically EG constitute layer‐by‐layer thin nanosheets to form composite with good conductivity. Benefiting from these features, PAE‐NiNiPcF_8_‐based interdigital MSCs show excellent *C*_v_ of 28.1 F cm^−3^ and excellent stability of 10 000 charge–discharge continuous cycling in acidic electrolytes. This also may highlight the way for development of promising COFs‐based solid‐state energy storage devices^[^ ^48^ ^]^ by optimizing the redox active sites, crystallinity, porosity as well as conductivity.

## Conflict of Interest

The authors declare no conflict of interest.

## Supporting information

Supporting Information

## Data Availability

Research data are not shared.
