# Supplementary material for: Polyarylether‐Based 2D Covalent‐Organic Frameworks with In‐Plane D–A Structures and Tunable Energy Levels for Energy Storage
Source: Adv Sci (Weinh). 2021 Dec 26;9(6):2104898. doi: 10.1002/advs.202104898 (PMC8867148; doi:10.1002/advs.202104898)
Supplement: Supplementary file 1 — Supporting Information [file ADVS-9-2104898-s001.pdf]

WILEY-VCH

ADVANCED SCIENCE
Open Access

Supporting Information

for *Adv. Sci.*, DOI: 10.1002/advs.202104898

Polyarylether-based Two-dimensional Covalent-organic Frameworks with In-plane D-A Structures and Tunable Energy Levels for Energy Storage

Nana Li, Kaiyue Jiang, Fermín Rodríguez-Hernández, Haiyan Mao, Sheng Han, Xiaobin Fu, Jichao Zhang, Chongqing Yang,* Changchun Ke,* Xiaodong Zhuang**

Supporting Information

Polyarylether-based Two-dimensional Covalent-organic Frameworks with In-plane D-A Structures and Tunable Energy Levels for Energy Storage

Nana Li, Kaiyue Jiang, Fermín Rodríguez-Hernández, Haiyan Mao, Sheng Han, Xiaobin Fu, Jichao Zhang, Chongqing Yang,* Changchun Ke,* Xiaodong Zhuang**

N. Li, Prof. S. Han
School of Chemistry and Chemical Engineering, Shihezi University, Shihezi, Xinjiang 832003, China. E-mail: hansheng654321@sina.com (S.H.)
N. Li, K. Jiang, Dr. C. Yang, Prof. X. Zhuang
The meso-Entropy Matter Lab, School of Chemistry and Chemical Engineering, State Key Laboratory of Metal Matrix Composites, Shanghai Key Laboratory of Electrical Insulation and Thermal Aging, Frontiers Science Center for Transformative Molecules, Shanghai Jiao Tong University, Shanghai 200240, China. E-mail: chongqing@sjtu.edu.cn (C.Y.); zhuang@sjtu.edu.cn (X.Z.)
Dr. F. Rodríguez-Hernández
Departamento de Química, Módulo 13, Universidad Autónoma de Madrid, 28049 Madrid, Spain.
Dr. H. Mao
Department of Chemical and Biomolecular Engineering, University of California, Berkeley, California 94720, United States
Dr. J. Zhang
Shanghai Synchrotron Radiation Facility, Zhangjiang Laboratory, Shanghai Advanced Research Institute Chinese Academy of Sciences, Shanghai 201204, China
Dr. X. Fu
Department of Molten Salt Chemistry and Engineering, Shanghai Institute of Applied Physics, Chinese Academy of Sciences, Shanghai 201800, China.
Dr. C. Ke
Institute of Fuel Cells, School of Mechanical Engineering, Shanghai Jiao Tong University, Shanghai 200240, China. E-mail: kechangchun@sjtu.edu.cn (C.K.)
Prof. S. Han
School of Chemical and Environmental Engineering, Shanghai Institute of Technology, Shanghai 201418, China.
K. Jiang
College of Chemistry and Molecular Engineering, Zhengzhou University, Zhengzhou, 450001, Henan, China

Content

S1. Model reaction 4

S2. Synthesis of phthalocyanine and PAE-2D COFs 4

S2.1 Synthesis of metal (2,3,9,10,16,17,23,24-octahydroxyphthalocyaninato) (II) ($MPcOH_8$, M= Ni, Zn, Cu) 4

S2.2 Synthesis of metal hexadecafluoro(phthalocyaninato) ($MPcF_{16}$, M= Zn, Ni, Cu) 7

S2.3 PAE-2D COFs synthetic procedures 8

S3. Electrochemical measurement for energy Levels 9

S4. Electrochemical measurement of microsupercapacitors 9

S5. Monomer materials characterization 13

S6. PAE-2D COFs material characterization 20

S6.1. Powder X-ray diffraction 20

S6.2. Solid state ^{13}C cross-polarization magic angle spinning NMR 21

S6.3. Fourier-transform infrared spectroscopy 21

S6.4. X-ray photoelectron spectroscopy 22

S6.5. X-ray absorption spectroscopy 25

S6.6. N_2 adsorption-desorption isotherms 28

S7. Chemical stability 29

S8. Optical and electronic structure characterization 30

S8.1. Ultraviolet-visible absorption spectra 30

S8.2. Ultraviolet photoelectron spectroscopy 31

S8.3. Cyclic voltammetry measurement for energy level 33

S8.4. Computational model of band structures and PDOS 34

S9. Microsupercapacitor experiments 35

S10. Extended PAE-2D COFs based on ZnPc monomer 44

S1. Model reaction

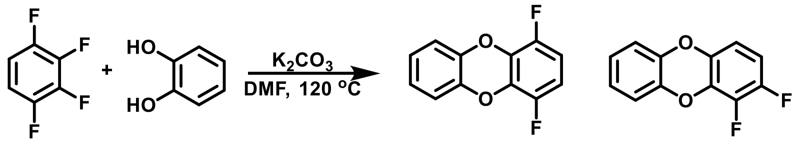

1 Yield: 83%

2 Not detected

Synthesis of molecular analog 1: Following a modified procedure from reference[1]. 1,2,3,4-tetrafluorobenzene (3.3 g mL, 22 mmol), catechol (2.20 g, 20.0 mmol), potassium carbonate (8.30 g, 60.0 mmol) and N,N-dimethylformamide (DMF) (60 mL) were placed into a flask under nitrogen atmosphere. The reaction mixture was stirred at 120 °C for 10 h, and then cooled to room temperature. The mixture was acidified with 1M HCl aq. to pH 4 and extracted with ethyl acetate (EtOAc). The organic layer was washed with water, dried over $MgSO_4$, and filtered. The filtrate was evaporated and the crude product was purified by a short pad of silica gel column chromatography (EtOAc) to yield the product (**1**) as a white powder (3.65 g, 16.6 mmol, 83%). ^{1}H NMR (500 MHz, DMSO-d_6): δ 7.10-7.02 (m, 4H), δ 7.02-6.98 (t, 2H); ^{13}C NMR (126 MHz, DMSO-d_6) δ 147.46 (d, J = 3.8 Hz), 140.41, 131.90 (dd, J = 13.0, 6.4 Hz), 125.74, 117.31, 110.66 (dd, J = 17.2, 11.2 Hz); ^{19}F NMR (471 MHz, DMSO-d_6): δ -139.53 (s, 2F).

S2. Synthesis of phthalocyanine and PAE-2D COFs

S2.1 Synthesis of (2,3,9,10,16,17,23,24-octahydroxyphthalocyaninato) metal (II) ($MPcOH_8$, M= Ni, Zn, Cu)

The synthesis of $MPcOH_8$ (M= Ni, Zn, Cu) was carried out according to the previous reported literatures with minor modification.[2]

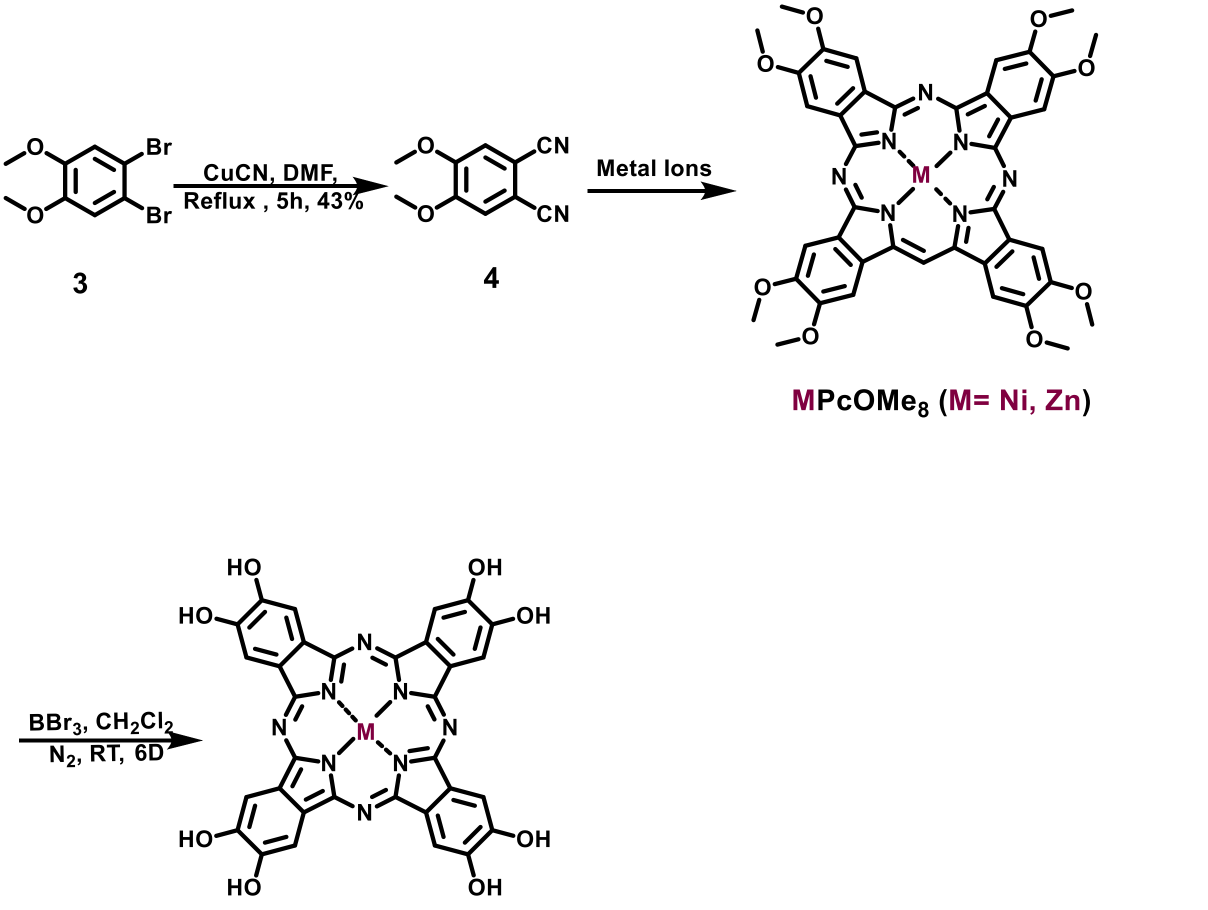

MPcOH$_8$ (M= Ni, Zn)

1,2-Dicyano-4,5-dimethoxybenzen: 1,2-Dibromo-4,5-dimethoxybenzene (25 g, 1 eq) was heated under reflux (bath temperature 165 °C) for 5 h with 22.7 g (3 eq) of CuCN in 350 mL of DMF. After being cooled, the reaction mixture was stirred in 1 L of concentrated ammonium hydroxide under air atmosphere overnight. The blue solution was suction filtered (sintered glass), and the solid residue was washed with a little dilute ammonium hydroxide and then with copious amounts of water until the filtrates were neutral. The dry, crude olive-green product was placed in the thimble of a Soxhlet extractor and extracted for 3 days with acetone. The white crude powder isolated from the acetone was purified through the silica gel column with dichloromethane and petroleum ether. Finally, the product was further crystallized from methanol: yield 6.84 g, 43 %; colorless small needles. ^{1}H NMR (500 MHz, $CDCl_3$, δ): δ 7.18 (s, 2H, Ar H); δ 4.00 (s, 6H, CH_3). ^{13}C NMR (126 MHz, $CDCl_3$, δ): δ 152.61, 115.77, 114.80, 108.92, 56.68.

Nickel (II) (2,3,9,10,16,17,23,24-octamethoxyphthalocyaninato) (NiPcOMe$_8$): **1,2-Dicyano-4,5-dimethoxybenzen** (5.0 g, 26.6 mmol), urea (1.6 g, 26.6 mmol), $NiCl_2$ (907 mg, 7 mmol), and ammonium molybdate (300 mg, 0.25 mmol) were refluxed in 100 mL of ethylene glycol under argon atmosphere for 4 days. The mixture was then cooled to room temperature, and 100

mL of water was added. Filtration gave a dark blue precipitate as the crude product. The precipitate purified by Soxhlet extraction with methanol (24 h), acetone (24 h) and chloroform (24 h). The product was then dried under vacuum to give (2,3,9,10,16,17,23,24-octamethoxyphthalocyaninato) Ni (II) as a dark green solid in 63% yield (3.40 g). MS (MALDI-TOF): calculated for $C_{40}H_{32}N_8O_8Ni$: 810.17 (100%), found: 810.08 (100%).

Zinc (II) (2,3,9,10,16,17,23,24-octamethoxyphthalocyaninato) (ZnPcOMe$_8$): **1,2-Dicyano-4,5-dimethoxybenzen** (4.00 g, 1 eq) and zinc(II) acetate (1.17 g, 0.25 eq) together with 1.28 g urea were stirred in N,N-dimethylacetamide (DMAC) (40 mL) at 140 ^{o}C for 3 days under the argon atmosphere. After cooling to room temperature, the reaction mixture was treated with a mixture of methanol and water (3/1in vol.; 100 mL), and the resulting solid obtained was filtered, washed with methanol and acetone. The obtained green sample was vacuum dried in ~70% yield. MS (MALDI-TOF): calculated for $C_{40}H_{32}N_8O_8Zn$: 816.16 (100%), found: 816.18 (100%).

(2,3,9,10,16,17,23,24-Octahydroxyphthalocyaninato) nickel (II) (NiPcOH$_8$): **NiPcOMe$_8$** (1.2 g, 14.76 mmol) was suspended in 50 mL of dried dichloromethane at room temperature, and BBr_3 (5.7 mL, 59 mmol, 4 e.q.) was added under argon atmosphere. The mixture was stirred for 6 days, and then slowly poured into 50 mL MeOH. The precipitate was collected by centrifugation. The obtained solid was then dispersed in MeOH (30 mL), shaken vigorously and then centrifuged. The solid was collected and then subjected by MeOH washing using centrifuge for another 4 times. The product was then dried under vacuum to give NiPcOH$_8$ as a dark green solid (0.75 g, yield 73%). ^{1}H NMR (500 M, DMSO-d_6): δ 8.60 (s, 8H), 10.37 (br, 8H). MS (MALDI-TOF): calculated for $C_{32}H_{16}N_8O_8Ni$: 698.22 (100%), found: 698.05 (100%).

zinc (II) (2,3,9,10,16,17,23,24-Octahydroxyphthalocyaninato) (ZnPcOH$_8$): The procedure for the synthesis of *ZnPcOH$_8$* was the similar with that of *NiPcOH$_8$*. Yield: 70%. ^{1}H NMR (pyridine-d_5): δ 9.57 (s, 8H), 6.79 (s, 8H). MS (MALDI-TOF): calculated for $C_{32}H_{16}N_8O_8Zn$: 704.04 (100%), found: 704.09 (100%).

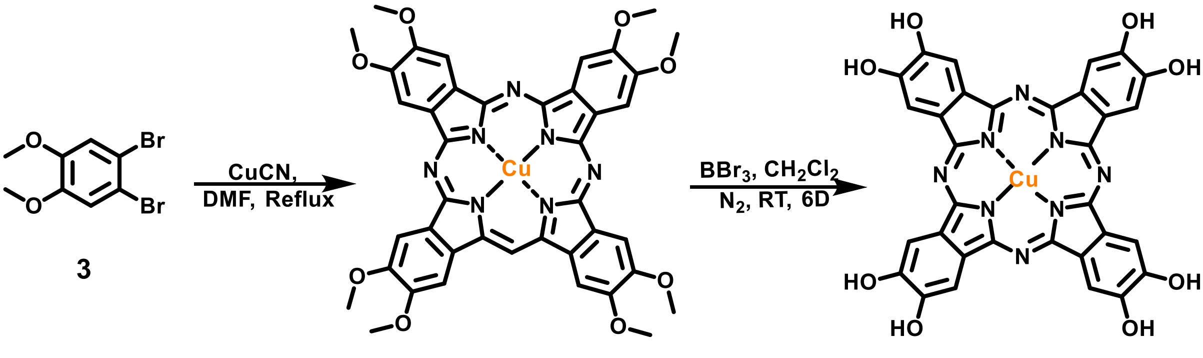

3

CuPcOMe8 CuPcOH8

Copper (II) (2,3,9,10,16,17,23,24-octamethoxyphthalocyaninato) (CuPcOMe$_8$): 1, 2-Dibromo-4,5-dimethoxybenzene (15.0 g, 0.05 mol) and 13.6 g (0.15 mol) of CuCN was refluxed in DMF (200 mL) at 165 ^{o}C for 3 days. After cooling at room temperature, 600 mL of concentrated ammonium hydroxide was added into the above reaction mixture and it was stirred at room temperature for 18 h under air atmosphere. Then the green solid was obtained by filtration and washing with copious amount of diluted ammonium hydroxide and water. The dry, crude olive-green product was placed in the thimble of a Soxhlet extractor and extracted with methanol, acetone and chloroform. The product was then dried under vacuum to give CuPcOMe$_8$ as a green solid (3.1 g, yield 30%). MS (MALDI-TOF): calculated for $C_{40}H_{32}N_8O_8Cu$: 815.16 (100%), found: 815.29 (100%).

Copper (II) (2,3,9,10,16,17,23,24-octahydroxyphthalocyaninato) (CuPcOH$_8$): The procedure for the synthesis of *CuPcOH$_8$* was the similar with that of *NiPcOH$_8$*. Yield: 60%. MS (MALDI-TOF): calculated for $C_{32}H_{16}N_8O_8Cu$: 703.04 (100%), found: 702.97 (100%).

S2.2 Synthesis of hexadecafluoro(phthalocyaninato) Metal (MPcF$_{16}$, M= Zn, Ni, Cu) [3]

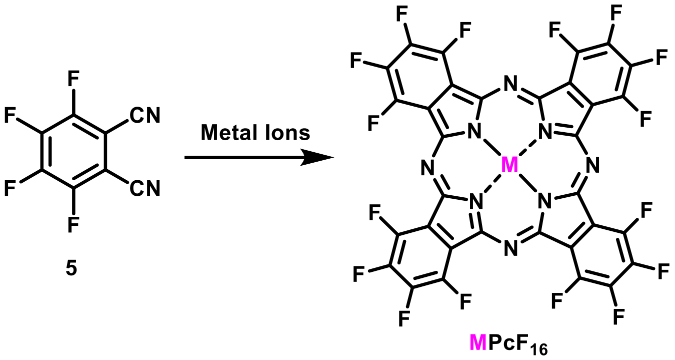

5

MPcF16

Synthesis of zinc (II) hexadecafluoro(phthalocyaninato) (ZnPcF$_{16}$): tetrafluorophthalonitrile (480 mg, 2.4 mmol) and dry zinc(II) acetate (110 mg, 0.6 mmol) was intensively mixed in a mortar. The mixture was filled in a glass vessel, three times flushed with nitrogen and vacuum, and finally the glass ampoule was sealed under vacuum. After heating for 2 h at 190 ^{o}C, the blue product was isolated and washed with water, acetone and petrol ether to remove impurities.

Yield 0.34 g (65%). MS (MALDI-TOF): calculated for $C_{32}F_{16}N_8Zn$: 863.93 (100%), found: 863.79 (100%).

The syntheses of ***CuPcF₁₆*** and ***NiPcF₁₆*** were performed similarly, starting from $CuCl_2$ (135 mg, 1 mmol, 45 mol% based on tetrafluorophthalonitrile), $Ni(OAc)_2$ (177 mg, 1 mmol, 37 mol% yield).

S2.3 PAE-2D COFs synthetic procedures

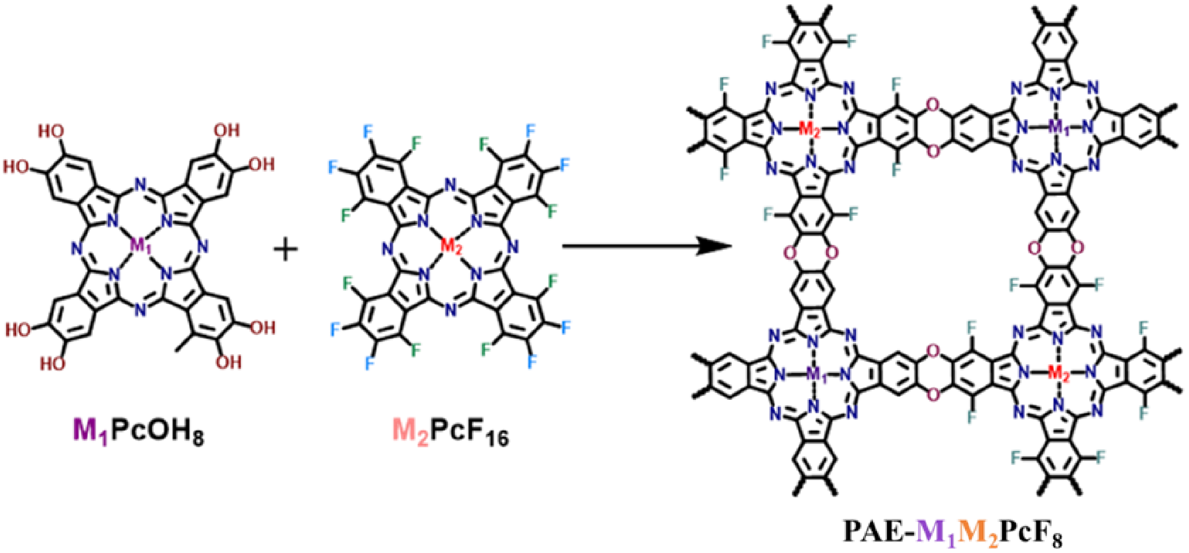

Synthesis of PAE-M₁M₂PcF₈

PAE-NiNiPcF₈: A Pyrex tube measuring 1.2 × 25 cm (o.d × length) was charged with $NiPcOH_8$ (25.0 mg, 0.035 mmol), $NiPcF_{16}$ (30.0 mg, 0.035 mmol), K_2CO_3 (97 mg, 0.7 mmol), DMAC (3.0 mL) in sequence. After sonication for about 20 minutes to disperse evenly, the tube was flash-frozen at 77 K (liquid N_2 bath) and degassed through three freeze-pump-thaw cycles by evacuated through an oil pump and then sealed under vacuum. After the temperature recovers to room temperature, the mixture was heated at 180 ºC and left undisturbed for 7 days. A black precipitate was isolated by filtration and washed with N-methyl-2-pyrrolidone (NMP), DMF, deionized water, dichloromethane, methanol and acetone until the filtrate was colorless. Finally, the product was evacuated at 60 ºC under vacuum overnight to yield activated samples (~40 mg).

PAE-CuCuPcF₈, PAE-NiCuPcF₈, PAE-ZnZnPcF₈, PAE-NiZnPcF₈, PAE-CuZnPcF₈: The synthesis of PAE-$CuCuPcF_8$, PAE-$NiCuPcF_8$, PAE-$ZnZnPcF_8$, PAE-$NiZnPcF_8$ and PAE-$CuZnPcF_8$ were carried out following the same protocol as PAE-$NiNiPcF_8$, by using phthalocyanines of different metals (M_1PcOH_8 and M_2PcF_{16}).

S3. Electrochemical measurement for energy Levels

Cyclic voltammetry (CV) was performed on a CHI 650E electrochemical analyzer in anhydrous CH_3CN containing recrystallized tetra-n butylammoniumhexafluorophosphate ($TBAPF_6$, 0.1 M) as supporting electrolyte at 298 K. A conventional three electrode cell was used with a glassy carbon working electrode (surface area of 0.3 mm^2) and a platinum wire as the counter electrode. The glassy carbon working electrode was routinely polished with a polishing alumina suspension and rinsed with acetone before use. The measured potentials were recorded with respect to Ag/AgCl reference electrode. The sample was wet-transferred onto the surface of a glassy carbon working electrode and let the solvent evaporate at room temperature for 30 min.

S4. Electrochemical measurement of microsupercapacitors

Synthesis of electrochemically exfoliated graphene: The electrochemically exfoliated graphene (EG) sheets were prepared according to previously reported method [4]. Typically, the natural graphite foil, a Pt wire, and 0.1 M $(NH_4)_2SO_4$ solution were acted as working electrode, counter electrode, and electrolyte, respectively. The distance between the graphite foil and the Pt electrode was about 2 cm during the electrochemical process. The electrochemical exfoliation of graphite was carried out by applying positive voltage at 10 V. After the graphite exfoliation was completed, the product was collected through a cotton fiber membrane filter with ~0.2 µm pore size and washed several times with deionized water by vacuum filtration. The resultant EG was then dispersed in NMP by sonication for 60 min. The dispersion was maintained for 2 days to precipitate un-exfoliated graphite flakes or particles. The supernatant dispersion of EG nanosheets could be directly used for device fabrication.

PAE-$M_1M_2PcF_8$/graphene hybrid and device fabrication: Typically, EG (~1 mg mL^{-1}) and PAE-$M_1M_2PcF_8$ (~0.3 mg mL^{-1}) were dispersion in NMP and mixed them with different volume ratios. After sonication for another 60 min, the mixture solution was stirred overnight to form a homogeneous suspension. Subsequently, the film was obtained by the suspension through vacuum filtration of with a polypropylene filter membrane (pore size of 0.22 µm) and washed with ethanol and deionized water respectively. After drying at room temperature, PAE-$M_1M_2PcF_8$/EG hybrid film was transferred to rigid glass slide substrate, then a thin Au layer was deposited these film surface. The interdigital electrode was fabricated by directly laser scribing. Then, 1 M PVA/H_2SO_4 gel electrolyte was drop-casted onto the surface of interdigital electrode and solidified overnight. Finally, all solid-state hybrid film-based in-plane microsupercapacitors (MSCs) were manufactured. For further discussion, the PAE-$M_1M_2PcF_8$/EG hybrid film MSCs were prepared based on different EG and PAE-NiNiPcF_8 weight ratios that were denoted as PAE-NiNiPcF_8/EG-3 (EG: PAE-NiNiPcF_8=3), PAE-

$NiNiPcF_8$/EG-5 (EG: PAE-$NiNiPcF_8$=5) and PAE-$NiNiPcF_8$/EG-7 (EG: PAE-$NiNiPcF_8$ =7). For comparison, the pure EG film-based MSC also was prepared under the same experimental condition. The PVA/H_2SO_4 gel electrolyte was prepared by mixing PVA (6 g) (molecular weight 85,000-124,000, Sigma-Aldrich) and H_2SO_4 (6 mL) in 60 ml deionized water, and heated at 85 °C under magnetic stirring until forming a clear solution. Finally, cooled naturally to room temperature. The H_2SO_4/PVA gel electrolyte was obtained for the experiment. If no otherwise specified, the PAE-$M_1M_2PcF_8$/EG hybrid film or PAE-$M_1M_2PcF_8$ hybrid film is PAE-$M_1M_2PcF_8$/EG-5.

Electrochemical characterization: All the electrochemical measurements including cyclic voltammetry (CV), galvanostatic charge-discharge (GCD) measurements, and electrochemical impedance spectroscopy (EIS) were conducted using an electrochemical workstation (CHI660E).

The specific capacitance values of the device are calculated from the CV data according to the equation (1) and (2):

$$C_A = \frac{1}{2v \times A \times (V_f - V_i)} \int_{V_i}^{V_f} I(V) dV \quad (1)$$

$$C_V = \frac{1}{2v \times V \times (V_f - V_i)} \int_{V_i}^{V_f} I(V) dV \quad (2)$$

where C_A is donated as specific areal capacitance (mF cm^{-2}) of PAE-$M_1M_2PcF_8$ /EG MSCs, v is the scan rate (V s^{-1}), V_f and V_i are the integration voltage limits of the voltammetric curve, and $I(V)$ is the voltammetric current with unit of ampere (A), A (cm^{-2}) is the areal of the entire device. C_V is donated as specific volume capacitance (F cm^{-3}) and V (cm^{-3}) is the volume of the entire device.

The specific areal capacitance (C_A, mF cm^{-2}) and specific volume capacitance (C_V, F cm^{-3}) of the entire MSCs on the basis of GCD curves can be obtained according to the following equation (3) and (4):

$$C_A = \frac{J \times \Delta t}{\Delta V} \quad (3)$$

$$C_V = \frac{C_A}{d} \quad (4)$$

where J is the current density (A m^{-2}) of charge/discharge, Δt is the discharged time (s), ΔV is voltage output window (V), and d is the thickness of PAE-$M_1M_2PcF_8$/EG film.

The electrochemical performance of the whole device showed in the Ragone plot is based on the volumetric stack capacitance from the CV data. The specific areal (E_A, μWh·cm^{-2}) and volumetric (E_V, Wh·cm^{-3}) energy densities are calculated from the equation (5) and (6):

$$E_A = \frac{1}{2} \times C_A \times \frac{(\Delta V)^2}{3600} \quad (5)$$

$$E_V = \frac{1}{2} \times C_V \times \frac{(\Delta V)^2}{3600} \quad (6)$$

The ΔV is the discharge potential range (in volts).

The specific areal (P_A, μW·cm^{-2}) and the volumetric (P_V, W·cm^{-3}) power densities are calculated according to the equation (7) and (8):

$$P_A = \frac{E_A}{\Delta t} \times 3600 \quad (7)$$

$$P_V = \frac{E_V}{\Delta t} \times 3600 \quad (8)$$

Δt is the discharge time in seconds.

Capacitance contribution calculation methods

The Trasatti method is used to differentiate the capacitance contribution from EDL capacitance (C_{dl}) and pseudocapacitance (C_p) reactions. CV measurements of PAE-NiNiPcF$_8$ are first collected with the scan rates ranging from 2 mV s^{-1} to 100 mV s^{-1}. Then, corresponding areal capacitances are evaluated based on the aforesaid equation. Plotting the reciprocal of areal capacitances (C^{-1}) against the square root of scan rates ($v^{-0.5}$) should yield a linear correlation between them. Specifically, the correlation can be described by the following equation (9):

$$C^{-1} = constant\ v^{0.5} + C_T^{-1} \quad (9)$$

where C is experimental areal capacitance, v is the scan rate and C_T was the total capacitance, respectively. The "total capacitance" equals the sum of EDL capacitance and pseudo-capacitance.

Plotting the areal capacitances (C) against the reciprocal of the square root of scan rates ($v^{0.5}$) should also give a linear correlation described by the following equation (10) (if assuming a semi-infinite diffusion of ions):

$$C = constant\ v^{-0.5} + C_{dl} \quad (10)$$

Linear fit the plot and extrapolate the fitting line to the y-axis gives the maximum EDL capacitance (C_{dl}). Subtraction of C_{dl} from C_T yield the maximum C_p.

Electrochemical quartz crystal microbalance analysis: The mass sensitivity of electrochemical quartz crystal microbalance (EQCM) originates from the relationship between the oscillation frequencies, as shown below equation (11):

$$\Delta m = -\frac{A\sqrt{\mu\rho}}{2f^2} \cdot \Delta f = -C_f \cdot \Delta f \quad (11)$$

where the EQCM sensor with the fundamental frequency of 7.946 MHz, A is the area of active surface (0.196 cm^2), μ is the AT-cut quartz constant (2.947 × 10^{11} g cm^{-1} s^{-2}), ρ is the quartz crystal density (2.84 g cm^{-3}), and then the sensitivity factor C_f is 1.42 ng Hz^{-1}.

The experimentally measured and theoretically calculated ion population changes ($\Delta\Gamma$) as a function of charge density (ΔQ). The experimental ion population change ($\Delta\Gamma_{exp}$) is calculated by the following equation (12):

$$\Delta\Gamma_{exp} = \frac{\Delta m}{M_i} \tag{12}$$

where Δm is the mass change, and the molecular mass (M_i) of $[H^+]_2[H_3O^+]$ and $SO4^{-2}$ is 19 and 96 g mol^{-1}, respectively. The theoretical ion population change ($\Delta\Gamma_{theor}$) is calculated with the adoption of Faraday's law equation (13):

$$\Delta\Gamma_{theor} = \frac{\Delta Q}{nF} \tag{13}$$

where ΔQ is the charge passed through the electrode (C), n is the valence number of the ion (n = 1), and F is the Faraday constant (96 485 C mol^{-1}).

S5. Monomer materials characterization

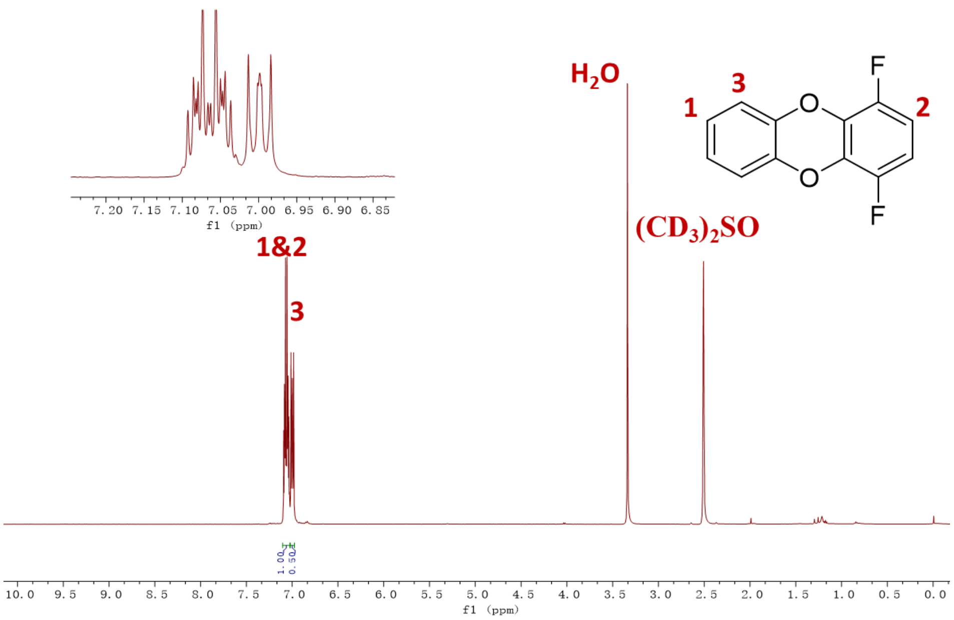

Figure S1. ^{1}H NMR specrum of **1**.

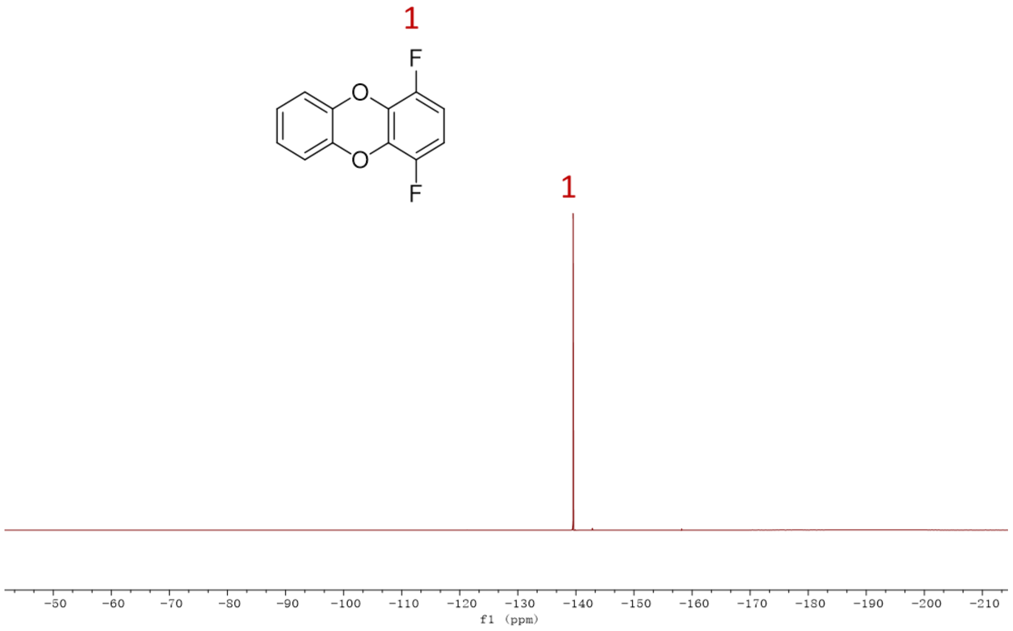

Figure S2. ^{19}F NMR spectrum of **1**.

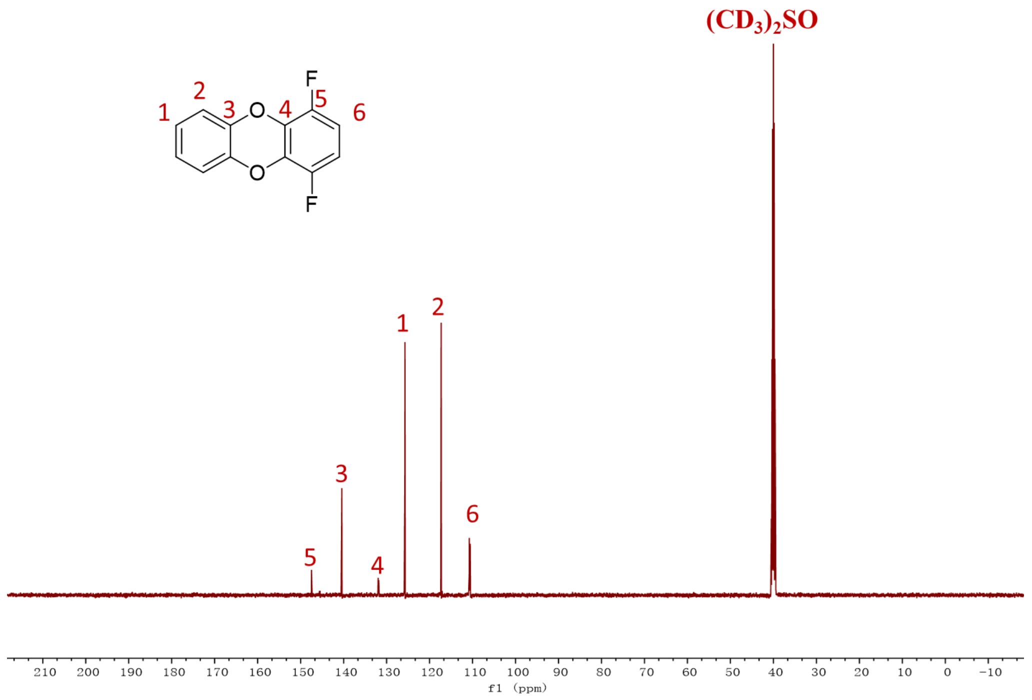

Figure S3. ^{13}C NMR spectrum of **1**.

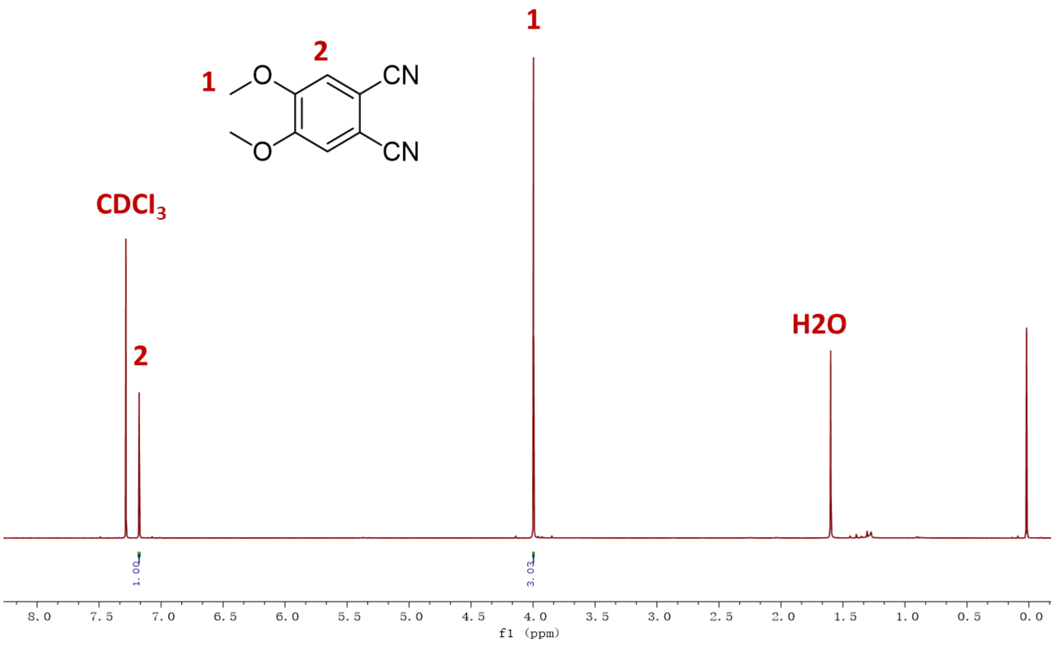

Figure S4. ^{1}H NMR spectrum for 1,2-dicyano-4,5-dimethoxybenzen.

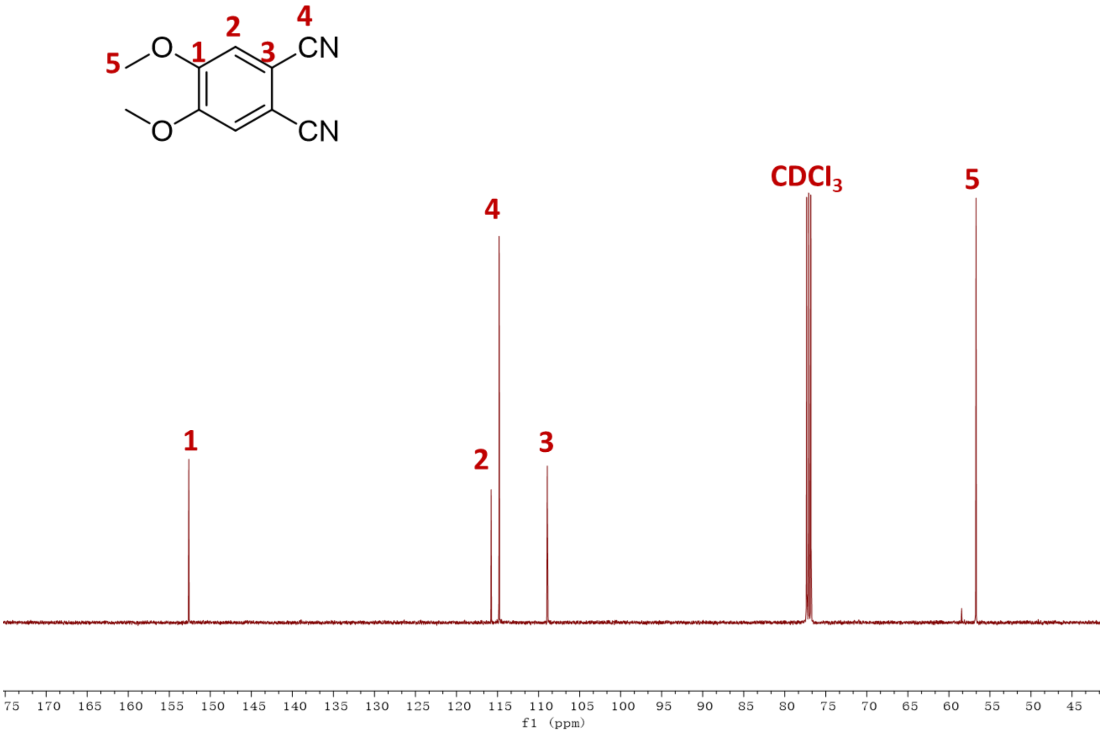

Figure S5. ^{13}C NMR spectrum of 1,2-dicyano-4,5-dimethoxybenzen.

a)

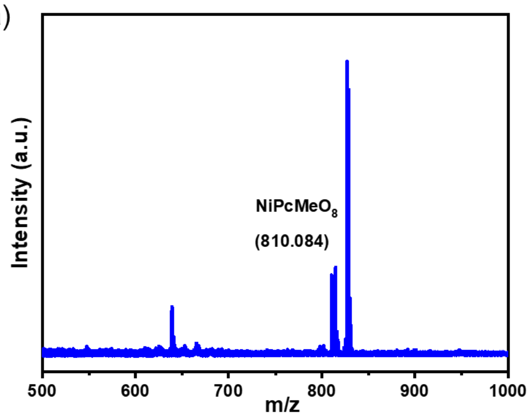

b)

Elements	N	C	H	O	Ni
Measured	11.24	50.56	4.26	21.27	6.49
Theoretical	13.32	59.95	4.55	15.21	6.97

Figure S6. a) MALDI-TOF MS and b) elemental analysis of $NiPcMeO_8$.

a)

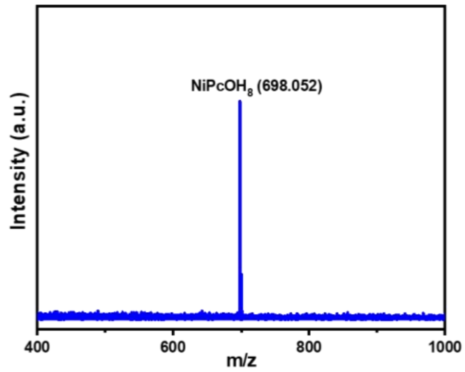

b)

Elements	N	C	H	O	Ni
Measured	13.50	48.14	3.18	23.66	6.49
Theoretical	15.37	56.00	3.04	17.55	8.05

Figure S7. a) MALDI-TOFMS and b) elemental analysis of $NiPcOH_8$.

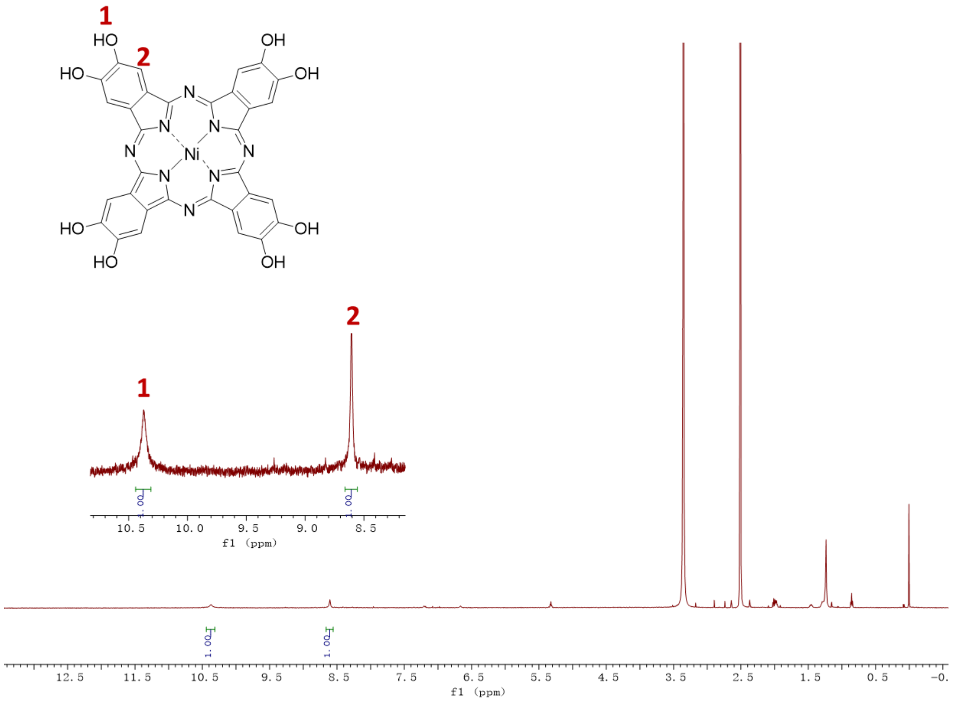

Figure S8. ^1H NMR spectrum of $NiPcOH_8$.

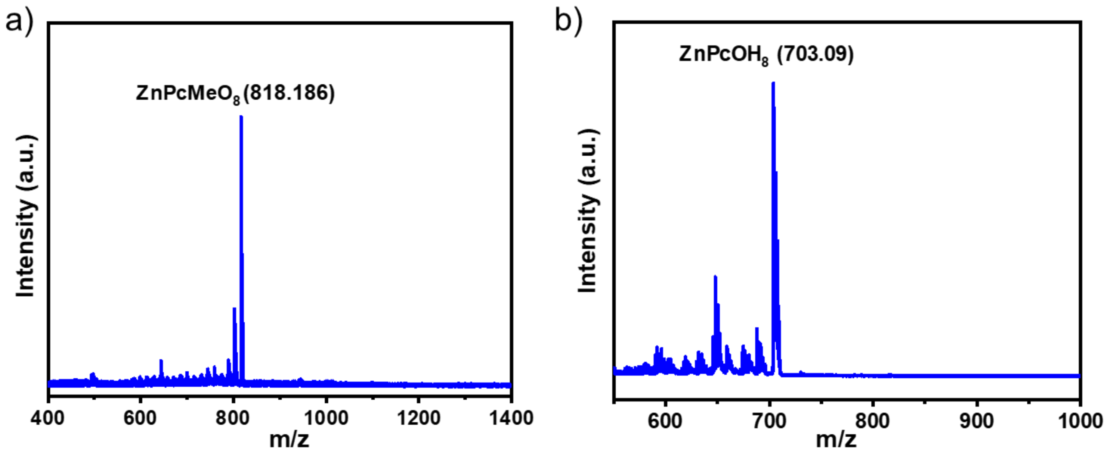

Figure S9. MALDI-TOF MS of a) $ZnPcMeO_8$ and b) $ZnPcOH_8$.

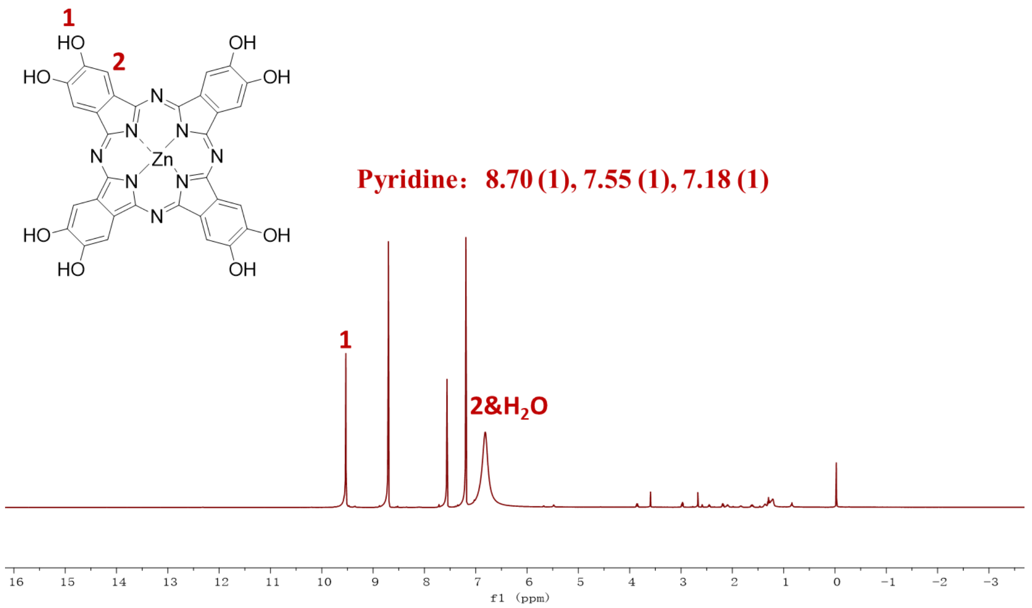

Figure S10. ^{1}H NMR specctrum of $ZnPcOH_8$.

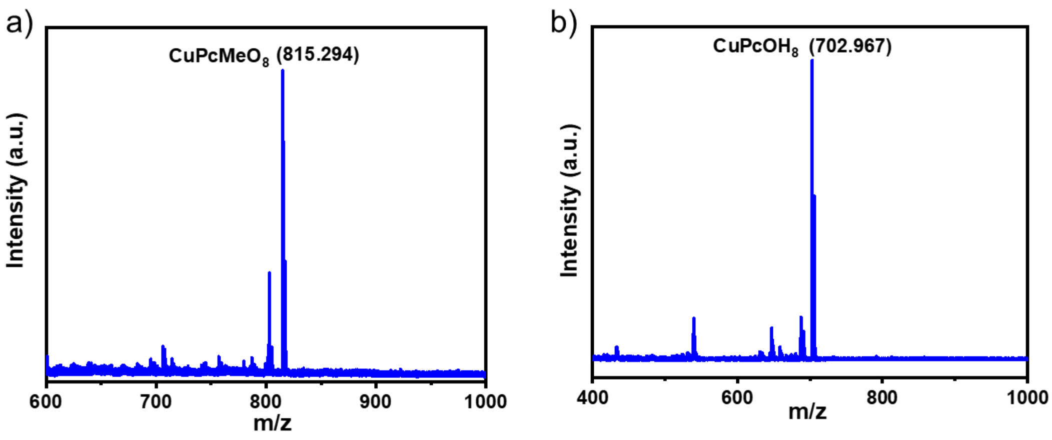

Figure S11. MALDI-TOF MS of a) $CuPcMeO_8$ and b) $CuPcOH_8$.

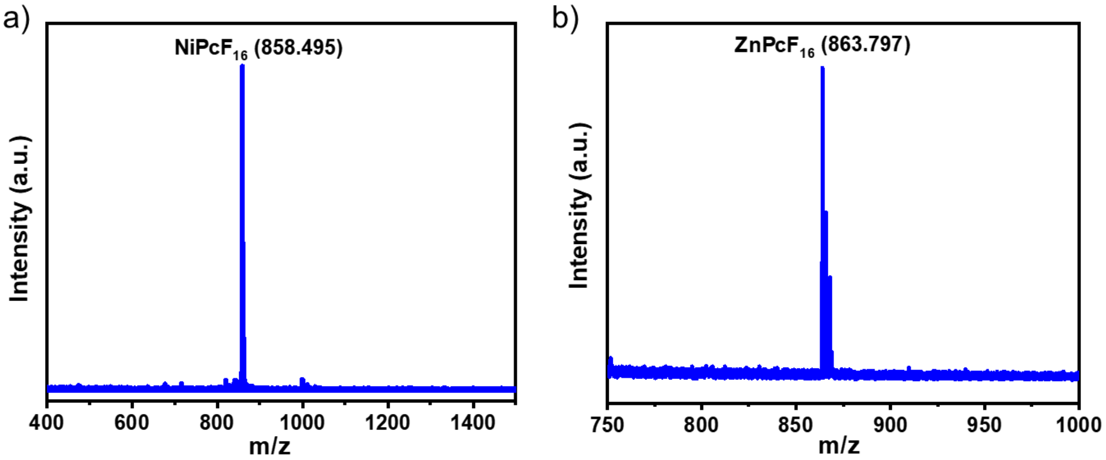

Figure S12. MALDI-TOF MS of a) $ZnPcF_{16}$ and b) $NiPcF_{16}$.

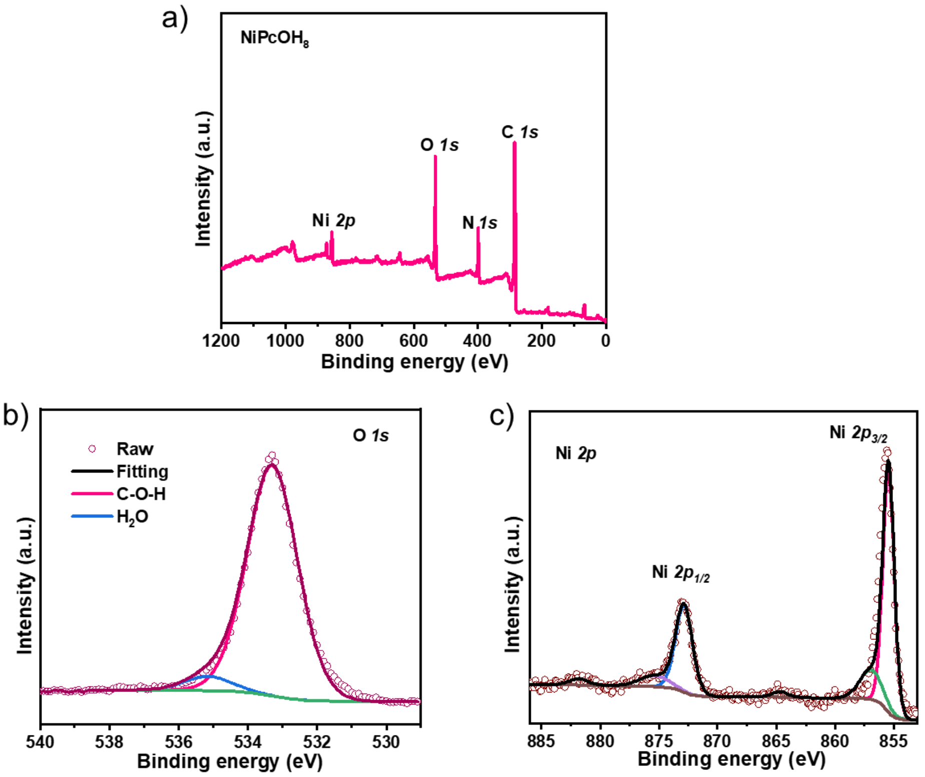

Figure S13. X-ray photoelectron spectroscopy survey spectrum a) and high-resolution spectra for b) O (*1s*) and c) Ni (*2p*) of $NiPcOH_8$.

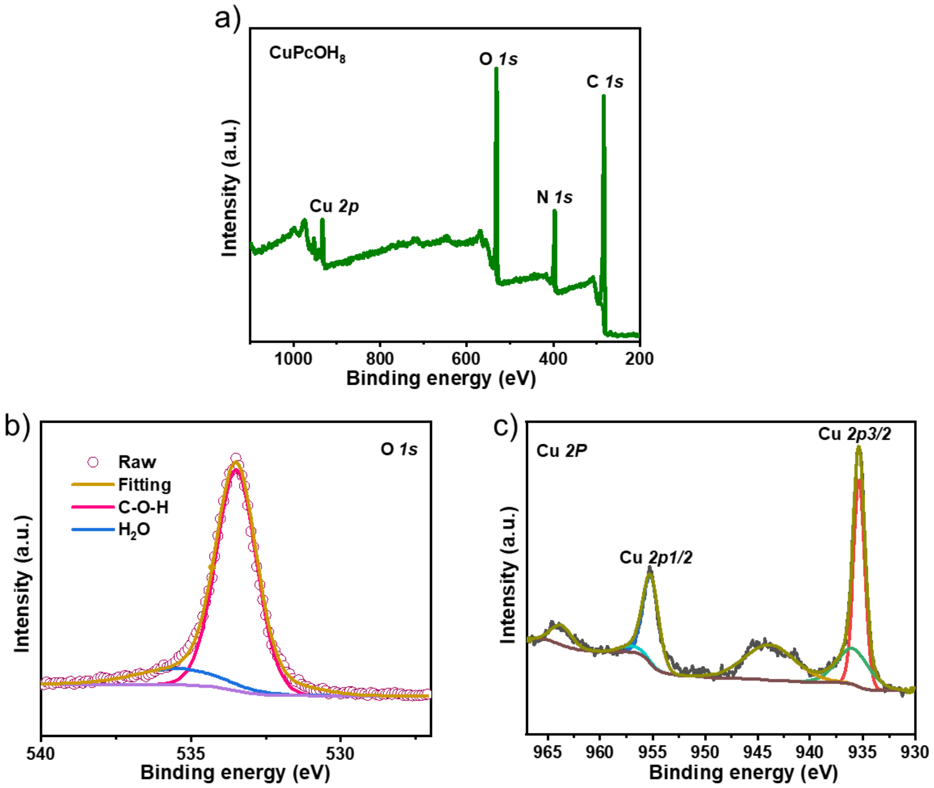

Figure S14. X-ray photoelectron spectroscopy survey spectrum a) and high-resolution spectra for b) O (*1s*) and c) Cu (*2p*) of $CuPcOH_8$.

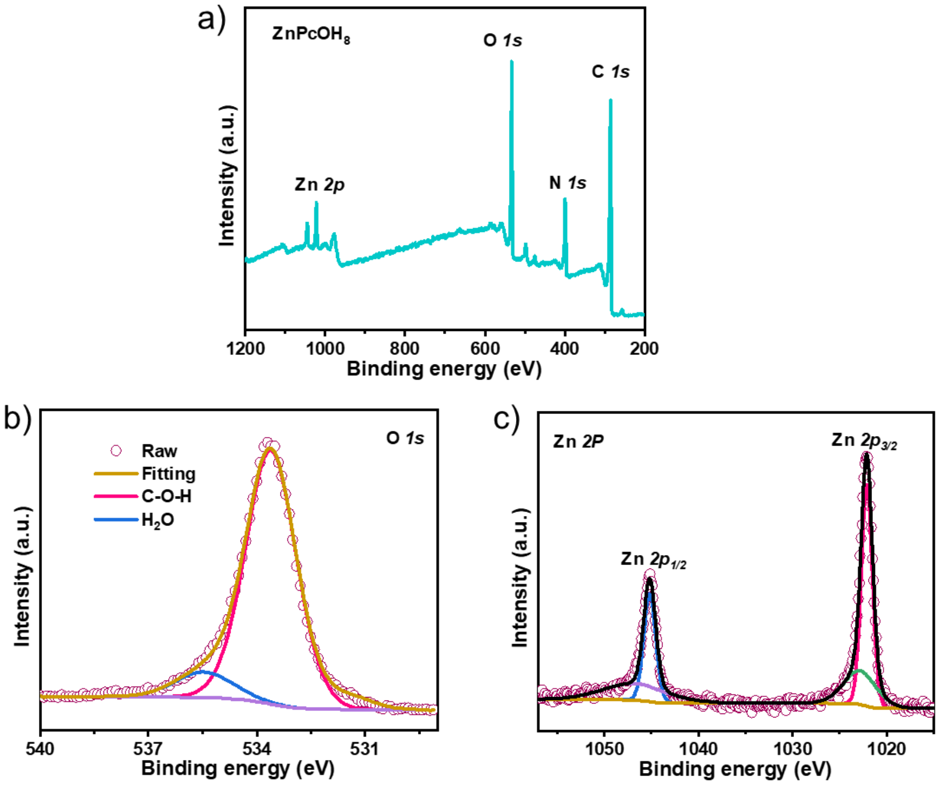

Figure S15. X-ray photoelectron spectroscopy survey spectrum a) and high-resolution spectra for b) O (*1s*) and c) Zn (*2p*) of $ZnPcOH_8$.

S6. PAE-2D COFs material characterization

S6.1. Powder X-ray diffraction

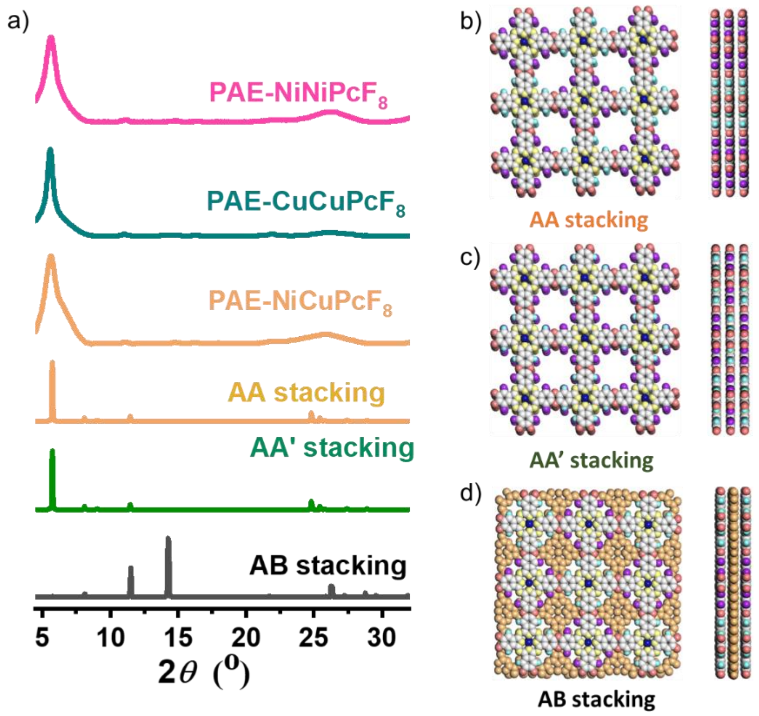

Figure S16. a) Experimental PXRD pattern of PAE-NiNiPcF$_8$ (red line), PAE-CuCuPcF$_8$ (wine red line) and PAE-NiCuPcF$_8$ (dark cyan line); calculated PXRD patterns for AA stacking (saffron yellow line), AA' stacking (dark green line) and AB stacking (dark line). b) Top view (left) and side view (right) of the AA stacking structure. c) Top view (left) and side view (right) of the AA' stacking structure. d) Top view (left) and side view (right) of the AB stacking structure.

S6.2. Solid state ^{13}C cross-polarization magic angle spinning NMR

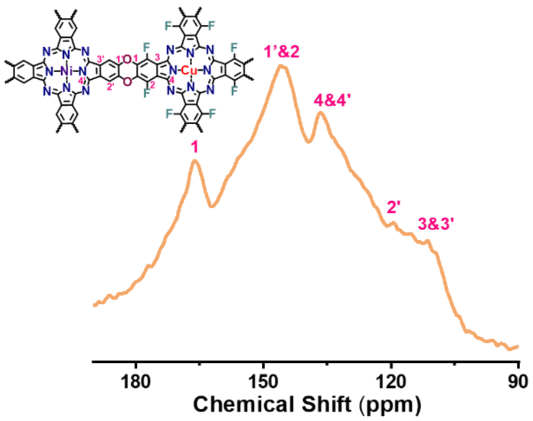

Figure S17. ^{13}C CP/MAS solid-state NMR spectrum for PAE-NiCuPcF$_8$. The resonance signals at 146 and 165 ppm are attributable to characteristic of carbons from C-O-C.

S6.3. Fourier-transform infrared spectroscopy

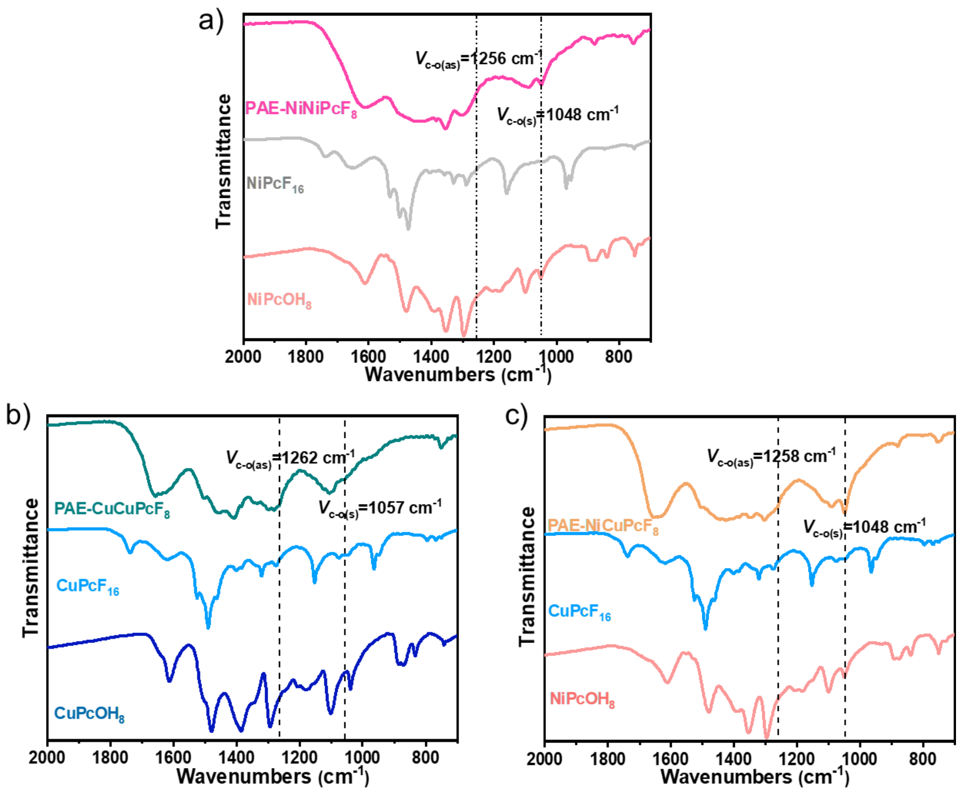

Figure S18. The FTIR spectra of PAE-M$_1$M$_2$PcF$_8$ and starting materials. The vibration bands at ~1260 cm^{-1} and ~1050 cm^{-1} belong to the characteristic peaks of dioxin C-O asymmetric and symmetric stretching modes.

S6.4. X-ray photoelectron spectroscopy

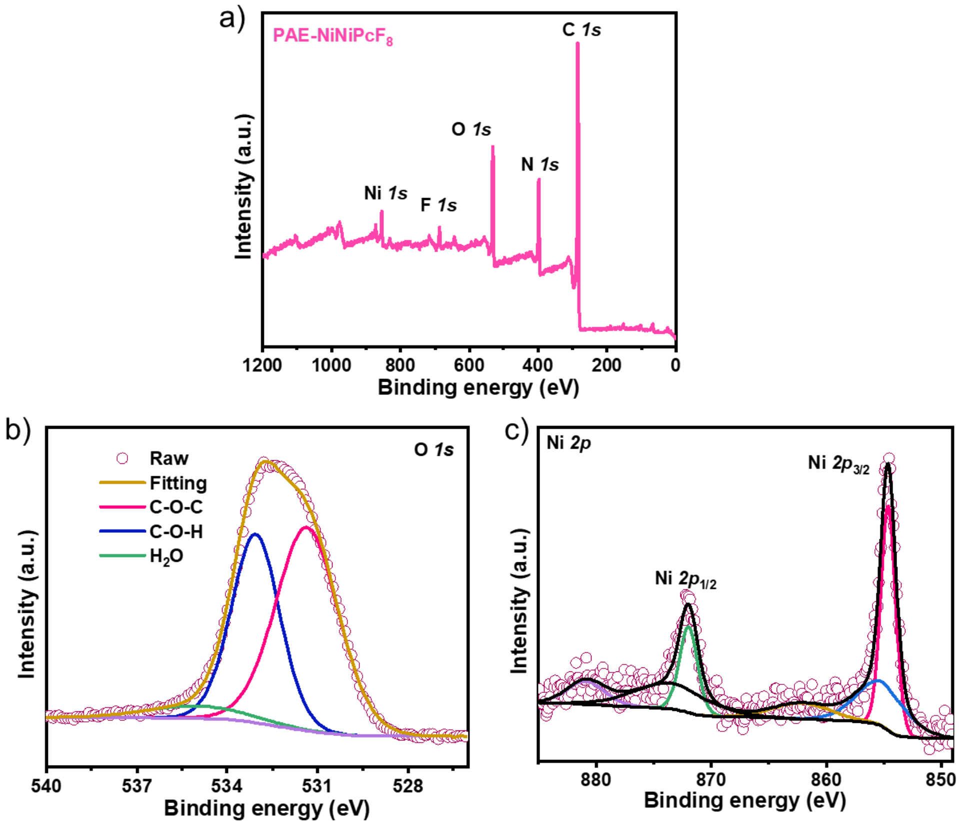

Figure S19. X-ray photoelectron spectroscopy survey spectrum a) and high-resolution spectra for b) O (1s) and c) Ni (2p) of PAE-NiNiPcF$_8$.

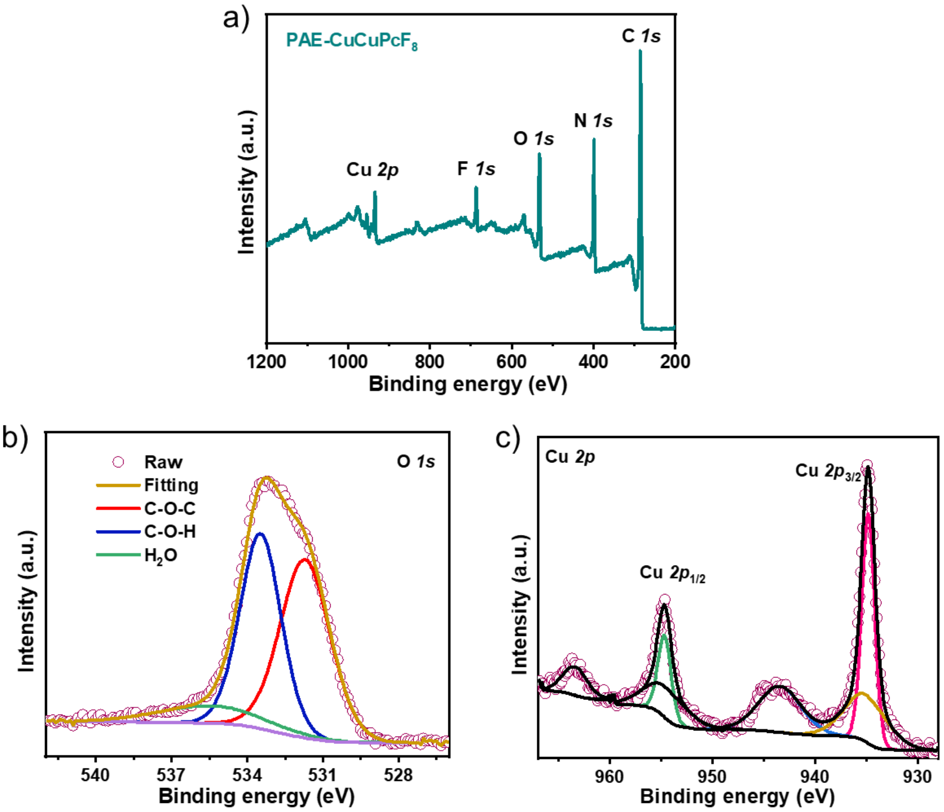

Figure S20. X-ray photoelectron spectroscopy survey spectrum a) and high-resolution spectra for b) O (*1s*) and c) Cu (*2p*) of PAE-CuCuPcF$_8$.

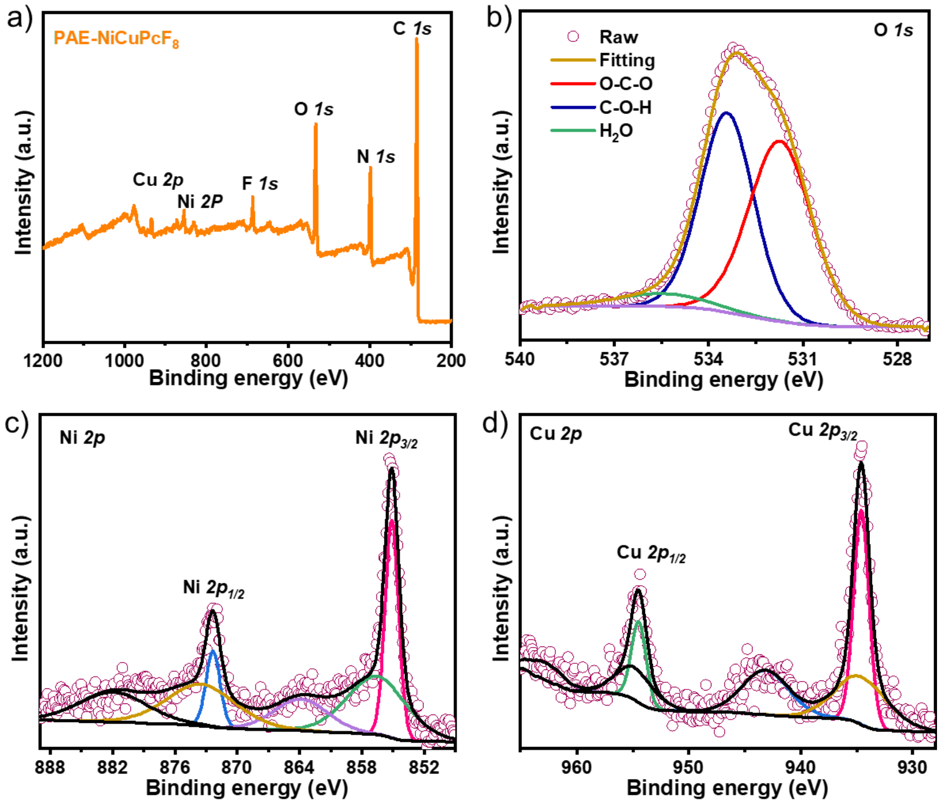

Figure S21. X-ray photoelectron spectroscopy survey spectrum a) and high-resolution spectra for b) O (*1s*), c) Ni (*2p*) and d) Cu (*2p*) of PAE-NiCuPcF$_8$.

S6.5. X-ray absorption spectroscopy

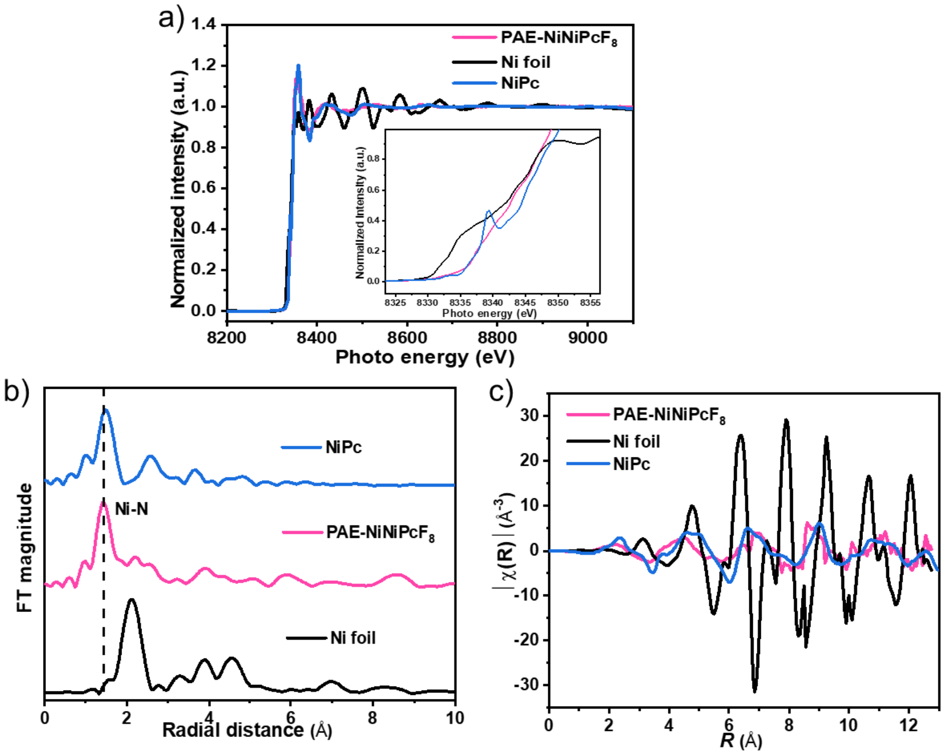

Figure S22. a) XANES of Ni K-edge. b) Fourier transform EXAFS spectra of Ni foil, NiPc and PAE-NiNiPcF$_8$. c) k^3-weighted k-space spectra.

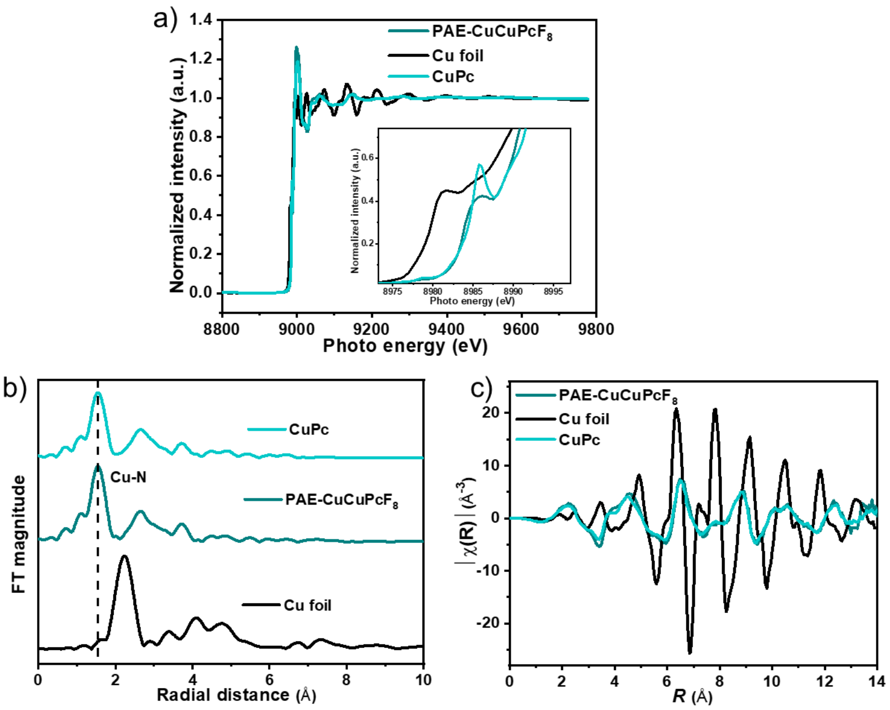

Figure S23. a) XANES of Cu K-edge. b) Fourier-transform EXAFS spectra of Cu foil, CuPc and PAE-CuCuPcF$_8$. c) k^3-weighted k-space spectra.

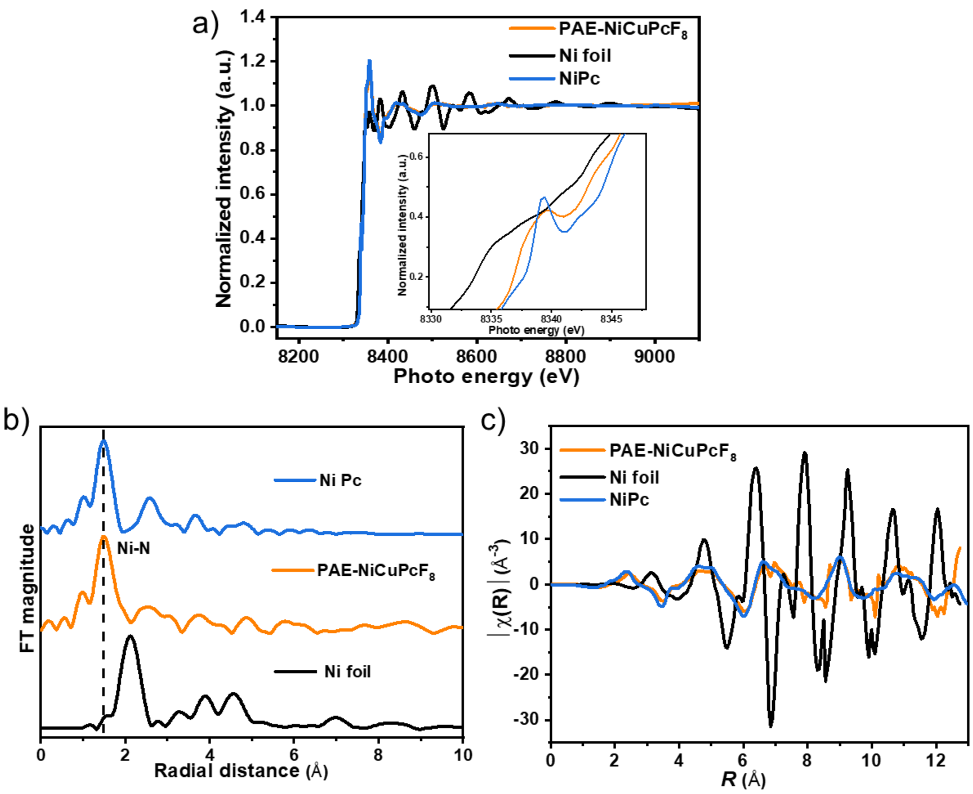

Figure S24. a) XANES of Ni K-edge. b) Fourier-transform EXAFS spectra of Ni foil, NiPc and PAE-NiCuPcF8. c) k^3-weighted *k*-space spectra. The Ni K-edge XANES spectra (Figure S24a) of PAE-NiCuPcF8 exhibits a typical Ni (II) peak at 8340 eV, which is similar to that of the reference NiPc. EXAFS of PAE-NiCuPcF8 (Figure S24b) displays the radial structure functions and clearly demonstrates the characteristic Ni-N coordination in PAE-NiCuPcF8 with intensive peak at around 1.4 Å.

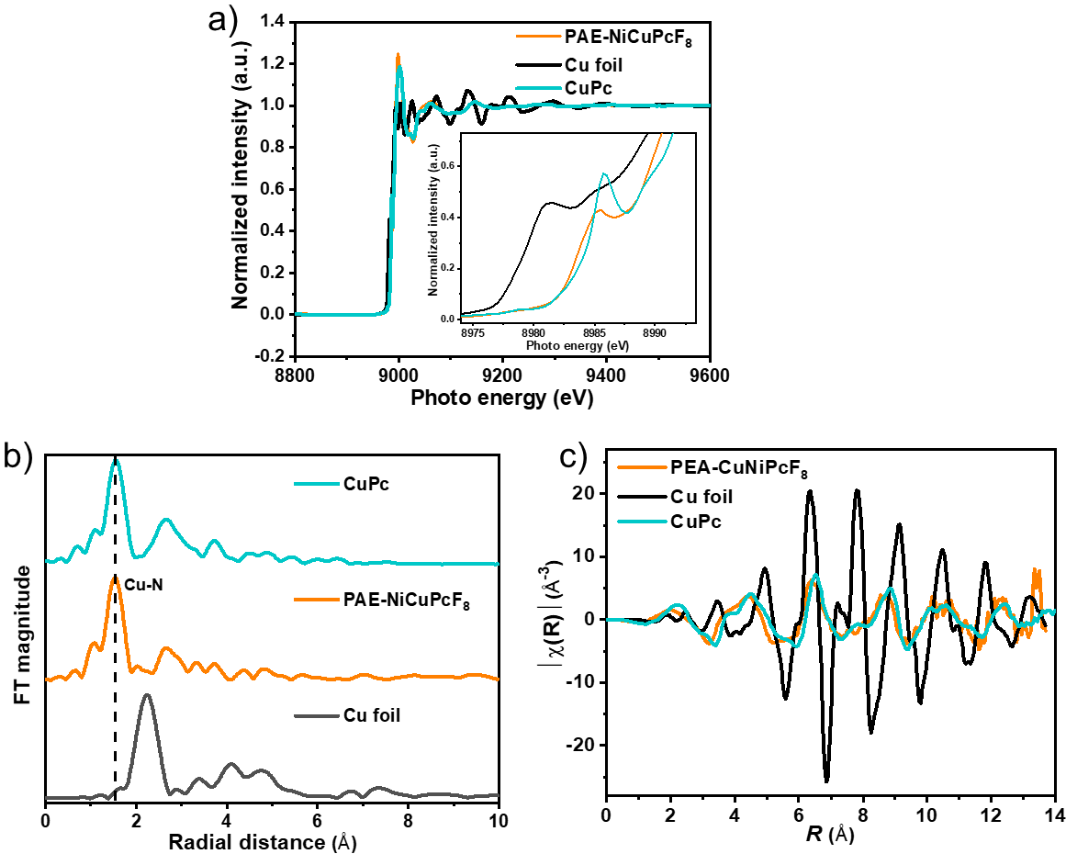

Figure S25. a) XANES of Cu K-edge. b) Fourier-transform EXAFS spectra of Cu foil, CuPc and PAE-NiCuPcF$_8$. c) k^3-weighted k-space spectra. The Cu K-edge XANES spectra of PAE-NiCuPcF$_8$ (Figure S25a) shows a typical Cu peak at 8985 eV (1s to 3d electron transition) indicating the oxidation valence of Cu atom as +2. EXAFS of PAE-NiCuPcF$_8$ (Figure S25b) reveals the existence of coordination bonds Cu-N coordination in PAE-NiCuPcF$_8$ with intensive peak at around 1.5 Å, in agreement with CuPc.

S6.6. N_2 adsorption-desorption isotherms

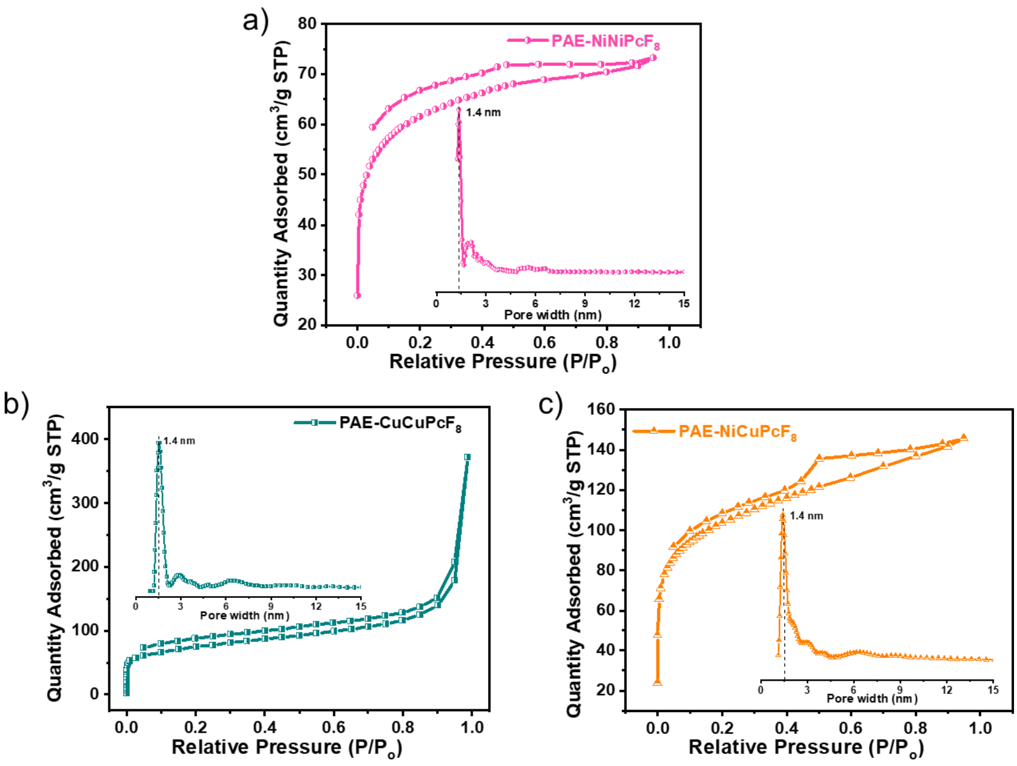

Figure S26. N_2 adsorption-desorption isotherms for a) PAE-NiNiPcF$_8$, b) PAE-CuCuPcF$_8$ and c) PAE-NiCuPcF$_8$. Insets: pore size distributions from fitting the quenched solid functional theory (QSDFT) model to the adsorption isotherms.

S7. Chemical stability

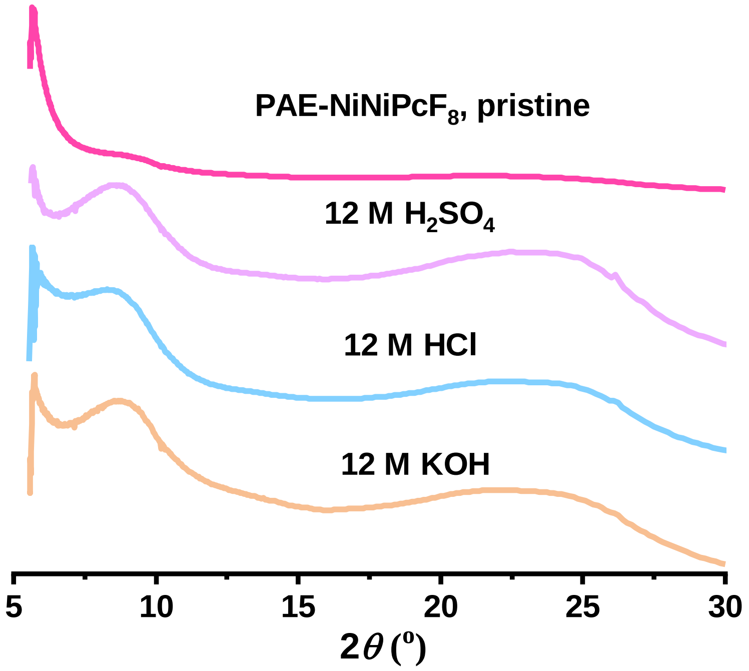

Figure S27. Chemical stability of PAE-NiNiPcF$_8$. The chemical stability of PAE-NiNiPcF$_8$ was examined by immersing it into different solvents (such as concentrated H_2SO_4 (12 M), concentrated HCl (12 M) and concentrated KOH (12 M)) at room temperature for at least 24 h. No obvious differences are observed from the PXRD after the treatment, indicating its structural robustness.

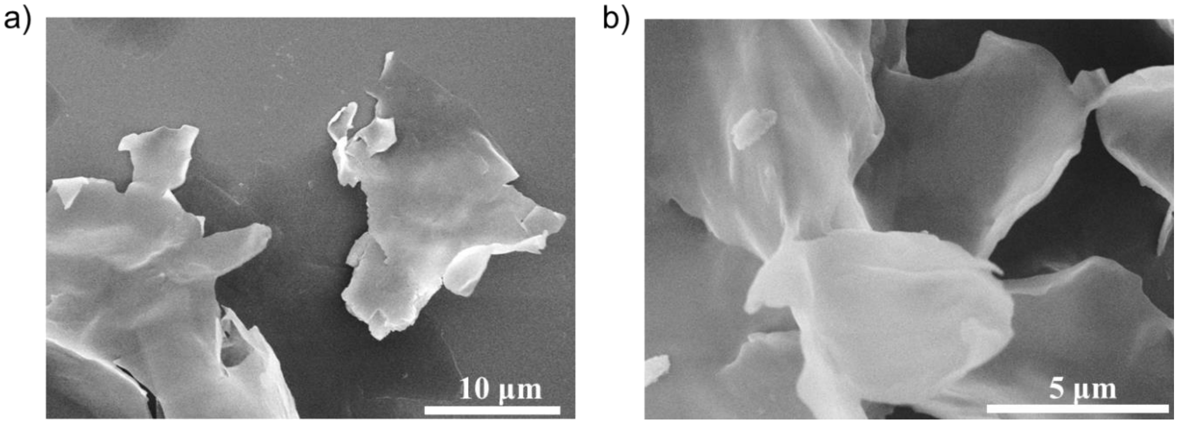

Figure S28. SEM of PAE-NiNiPcF$_8$-CST. First, disperse PAE-NiNiPcF$_8$-CST in acetone solution by ultrasonic, then scanning electron microscopy (SEM) were performed to characterize the morphology of PAE-NiNiPcF$_8$-CST, which showed a morphology of sheet structure with varying sizes from 5 to 20 µm.

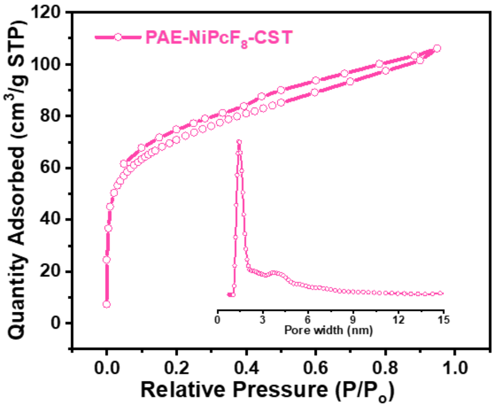

Figure S29. BET of PAE-NiNiPcF8-CST. The BET surface area also retained well with values around 223.76 m^2 g^{-1} for PAE-NiNiPcF8-CST

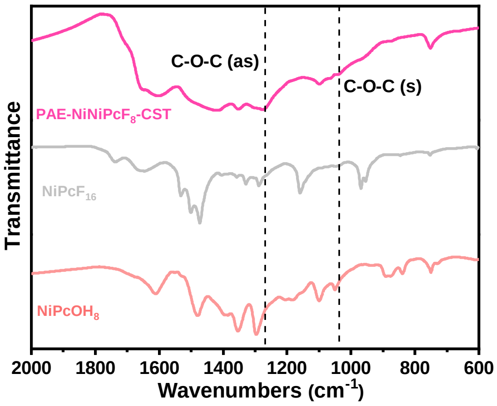

Figure S30. FT-IR spectra of PAE-NiNiPcF8-CST and start material. The FTIR spectrum of PAE-NiNiPcF8-CST sample display the obvious asymmetric and symmetric stretching peak of C-O-C at ~1256 and ~1048 cm^{-1}, respectively, that is the same as PAE-NiNiPcF8.

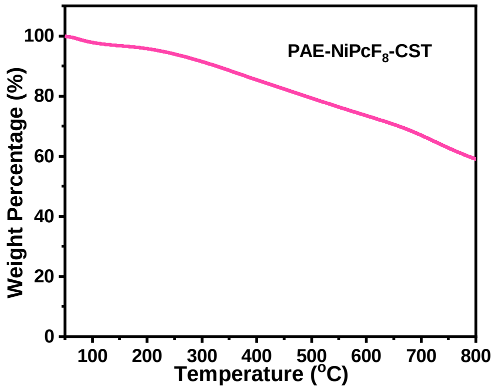

Figure S31. TGA profile of PAE-NiNiPcF8-CST under N_2 atmosphere. TGA results indicates that PAE-NiNiPcF8-CST was stable up to 400 °C

S8. Optical and electronic structure characterization

S8.1. Ultraviolet-visible absorption spectra

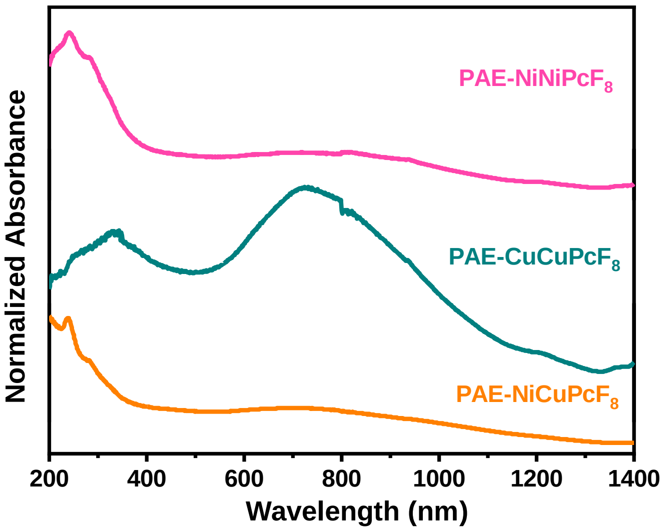

Figure S32. The UV-vis-NIR spectra of PAE-NiNiPcF$_8$, PAE-CuCuPcF$_8$ and PAE-NiCuPcF$_8$.

S8.2. Ultraviolet photoelectron spectroscopy

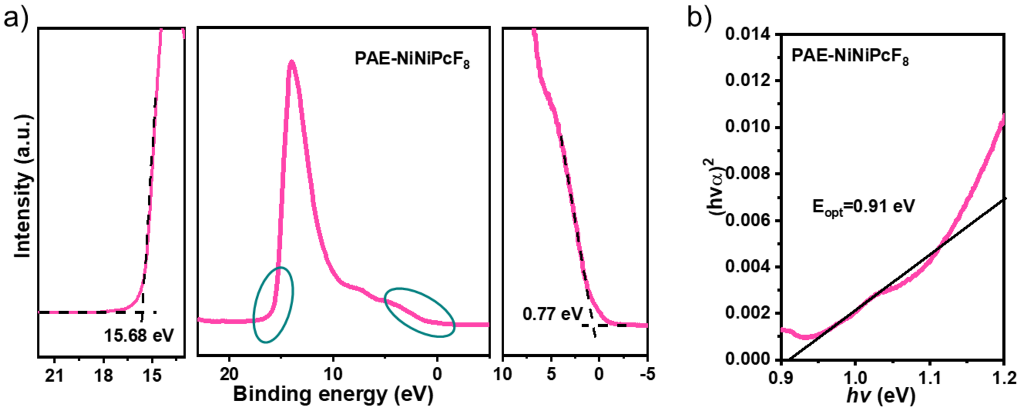

Figure S33. a) UPS spectrum and b) $(h\nu\alpha)^2$ versus $h\nu$ curve of PAE-NiNiPcF$_8$. The value at the intersection baseline and the tangent of the curve is the bandgap (E_{opt} = 0.91 eV).

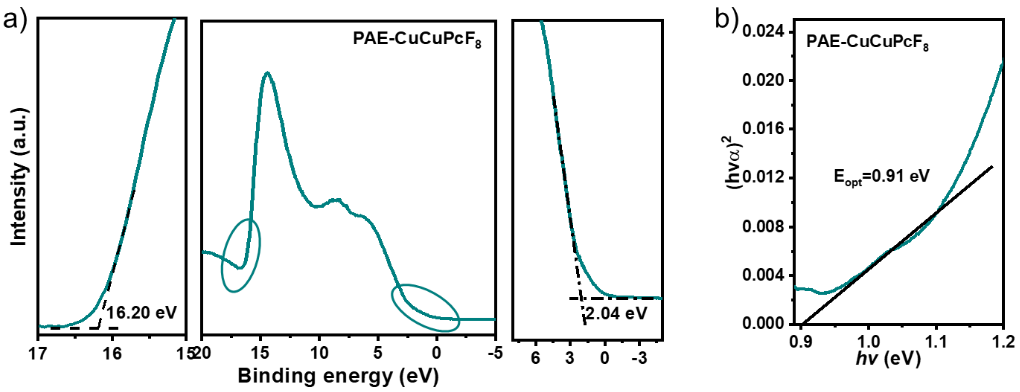

Figure S34. a) UPS spectrum and b) $(h\nu\alpha)^2$ versus $h\nu$ curve of PAE-CuCuPcF$_8$. The value at the intersection baseline and the tangent of the curve is the bandgap (E_{opt} = 0.91 eV).

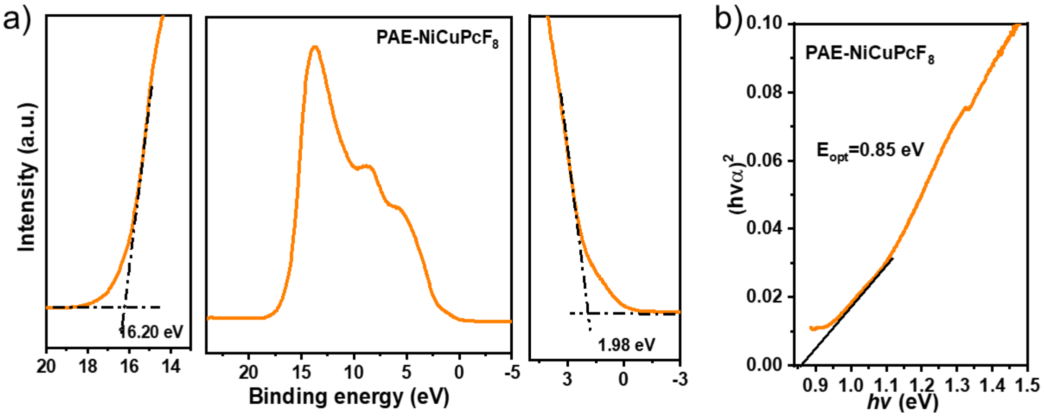

Figure S35. a) a) UPS spectrum and b) $(h\nu\alpha)^2$ versus $h\nu$ curve of PAE-$NiCuPcF_8$. The value at the intersection baseline and the tangent of the curve is the bandgap (E_{opt} = 0.85 eV).

S8.3. Cyclic voltammetry measurement for energy level

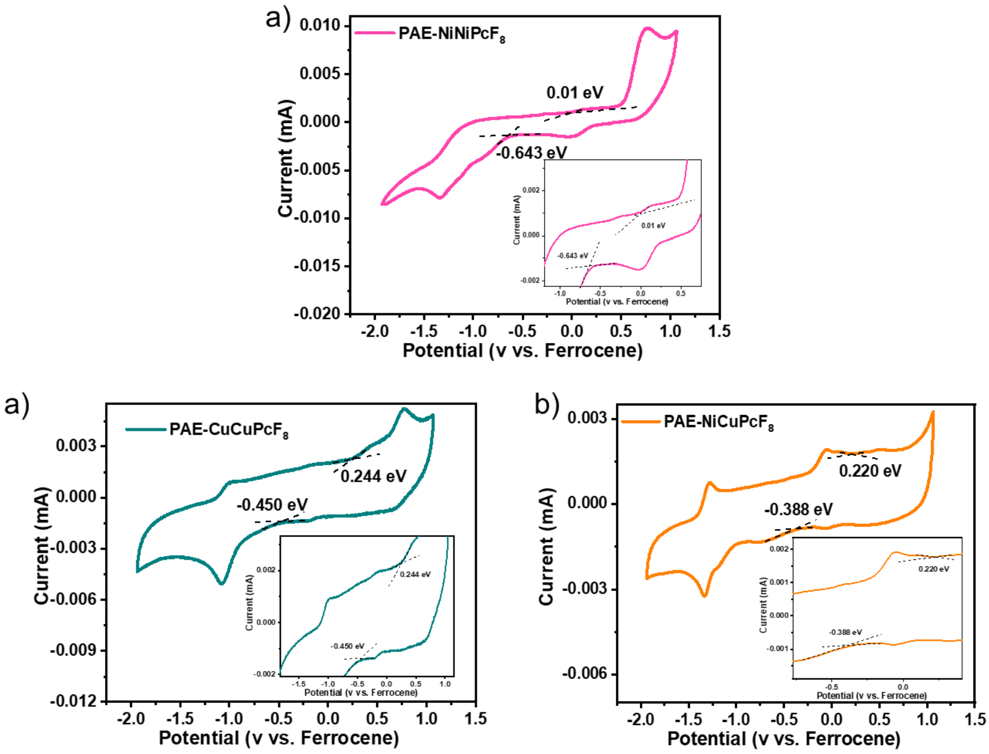

Figure S36. The cyclic voltammograms of the a) PAE-NiNiPcF$_8$, b) PAE-CuCuPcF$_8$ and c) PAE-NiCuPcF$_8$ measured in CH_3CN at a scan rate of 20 mV·s^{-1}.

S8.4. Computational model of band structures and PDOS

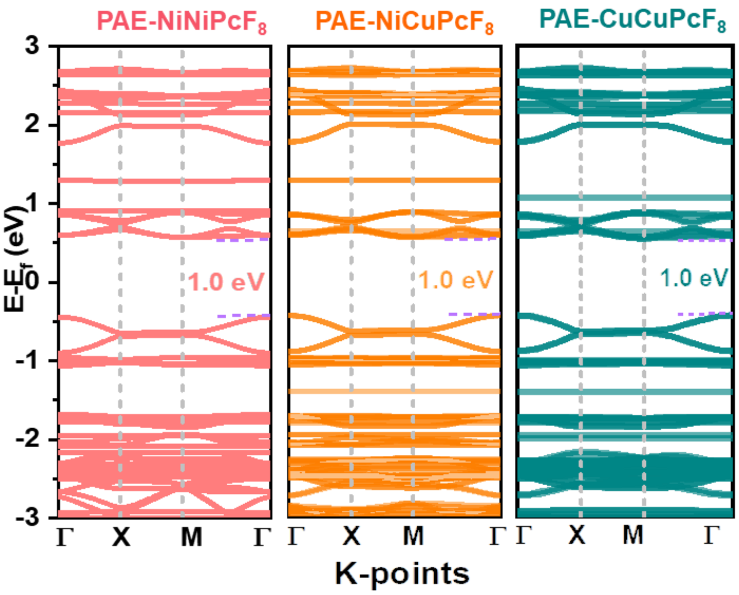

Figure S37. The calculated band structures of monolayer for PAE-NiNiPcF8, PAE-CuCuPcF8 and PAE-NiCuPcF8. Almost dispersionless CB and VB, implying that in-plane charge transport for monolayer was practically null because of an extremely large effective mass for charge carriers. Such similar band structures indicate that different metal center does not change the band gap of monolayer.

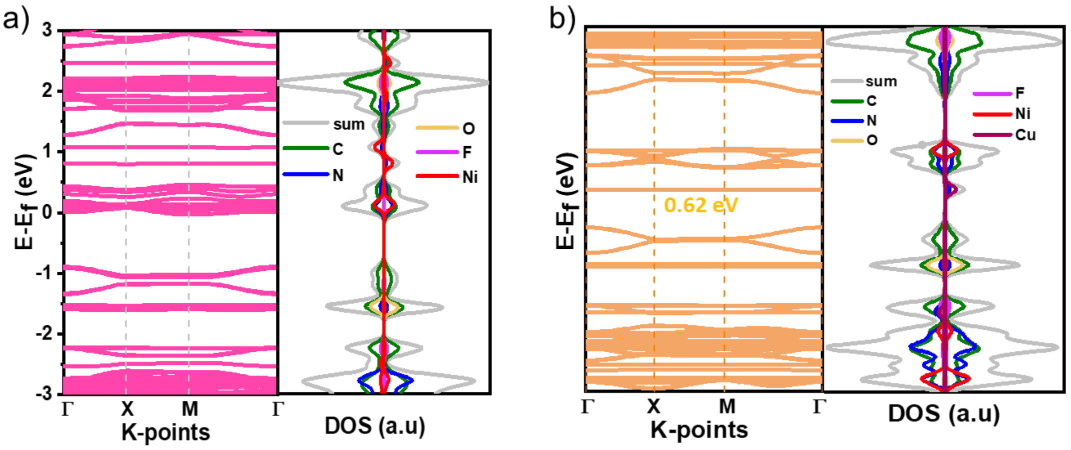

Figure S38. The calculated band structures and density of states (DOS) of AA stacking for a) PAE-NiNiPcF8 and b) PAE-NiCuPcF8. For AA stacking, the band gap of PAE-NiNiPcF8 and PAE-NiCuPcF8 are narrowed to 0 eV and 0.62 eV with respect to the monolayer (~1.0 eV) as result of the interlayer π-π interaction. The DOS plots of PAE-NiNiPcF8 and PAE-NiCuPcF8 indicate that both C and O contribute to the VBM, whereas CBM is dominated by C and N atoms. Thus, this suggests that the Pc mainly contributes to the conduction band, in contrast, the polyarylether unit is dominant in the valence band.

S9. Microsupercapacitor experiments

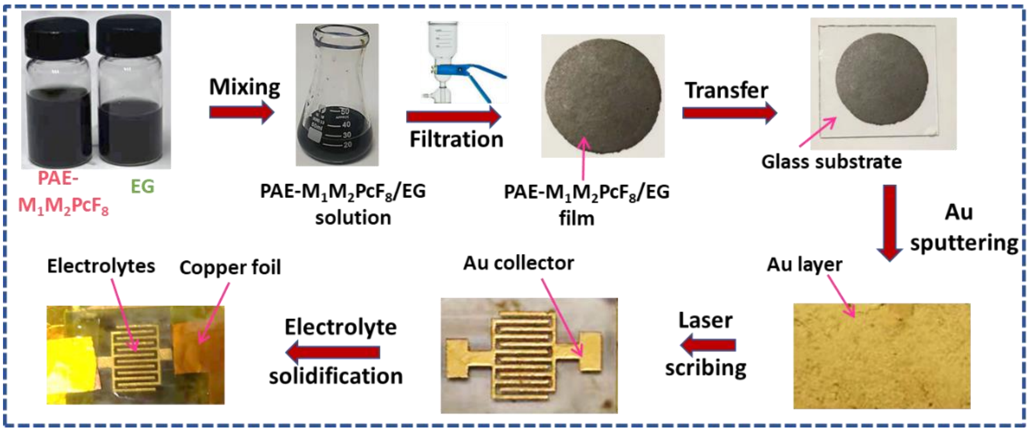

Figure S39. Schematic fabrication of PAE-$M_1M_2PcF_8$ based MSCs. First, the PAE-$M_1M_2PcF_8$/EG solution was prepared from the combination of PAE-$M_1M_2PcF_8$ and EG in NMP at room temperature. Then, PAE-$M_1M_2PcF_8$/EG hybrid films were fabricated through vacuum filtration that was transferred onto transparent glass substrates. Subsequently, the as-prepared films were deposited using Au and then laser-scribed to rapidly produce interdigitated electrodes. After that, electrolyte was drop casted on the interdigital electrodes and overnight solidification.

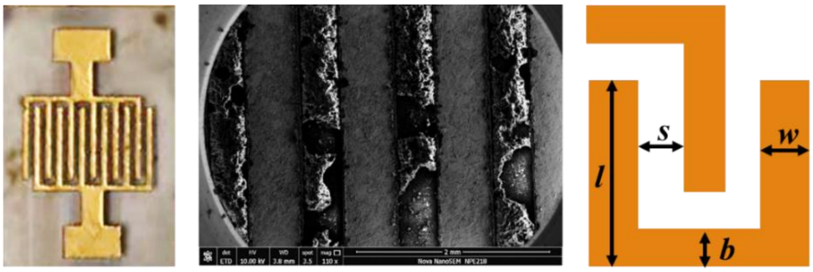

Interdigital Finger Number, N	12
Finger Width, *w* / mm	0.5
Finger Length, *l* / mm	7
Interspatial Distance, *s* / mm	0.5
Edge Breath, *b* / mm	0.5
Total Surface Area / mm^2	49.5

Figure S40. The parameters of the prepared PAE-$M_1M_2PcF_8$/EG hybrid film-based MSCs.

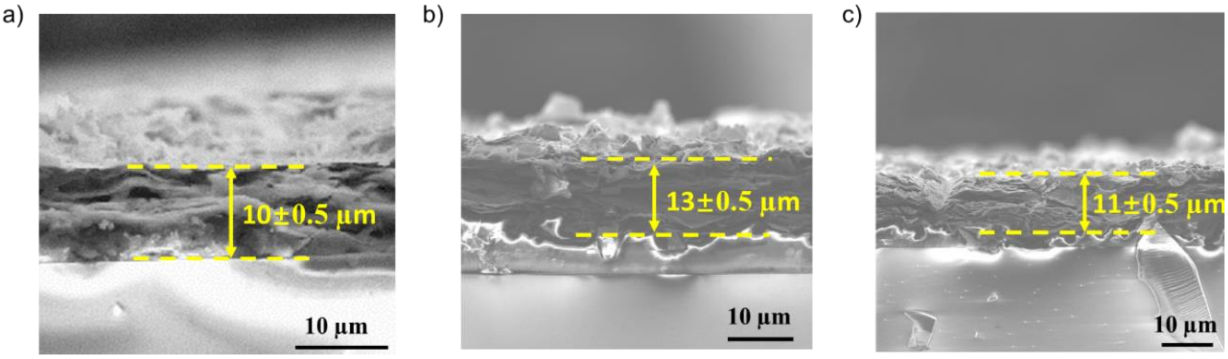

Figure 41. Cross-section view SEM images of a) PAE-NiNiPcF$_8$/EG, b) PAE-CuCuPcF$_8$/EG and c) PAE-NiCuPcF$_8$/EG electrode layered structure. All the side-view SEM images have the same morphology with layer-by-layer structure. By error correction, the film thickness of PAE-NiNiPcF$_8$/EG, PAE-CuCuPcF$_8$/EG and PAE-NiCuPcF$_8$/EG is ~10, ~13 and ~11 µm respectively.

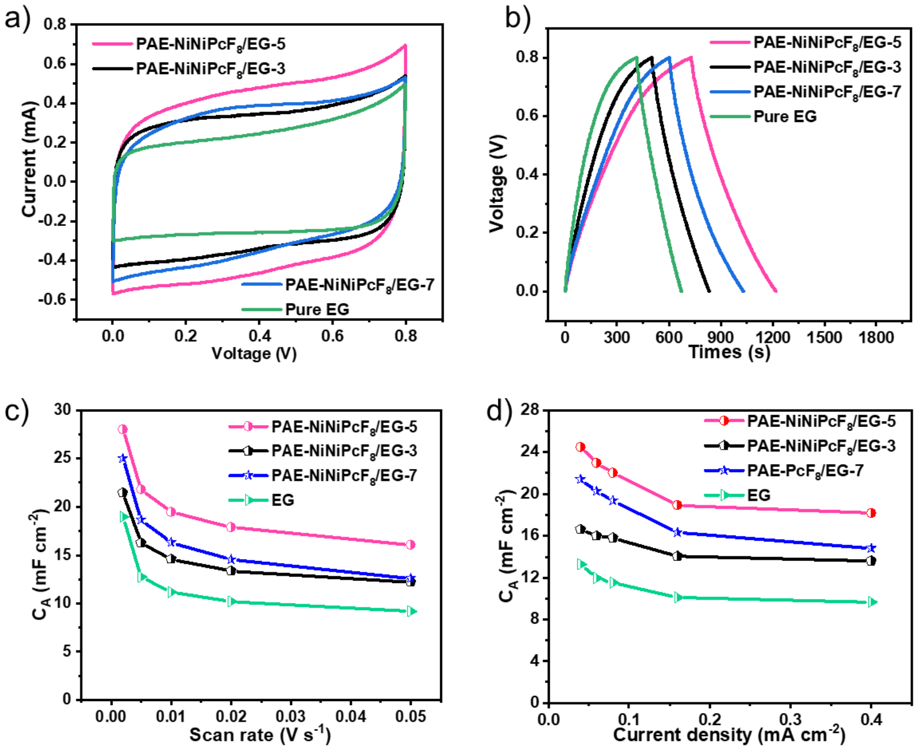

Figure S42. Supercapacitor performance of PAE-NiNiPcF$_8$/EG samples based on pure EG and different EG and PAE-NiNiPcF$_8$ weight ratios that were denoted as PAE-NiNiPcF$_8$/EG-3 (EG: PAE-NiNiPcF$_8$=3), PAE-NiNiPcF$_8$/EG-5 (EG: PAE-NiNiPcF$_8$=5) and PAE-NiNiPcF$_8$/EG-7 (EG: PAE-NiNiPcF$_8$=7) at PVA/H_2SO_4: a) CV curves at 50 mV s^{-1}; b) GCD curves at a current density of 0.04 mA cm^{-2}; c) specific areal capacitance calculated from CV curves at different scan rates; d) specific areal capacitance as a function of current densities based on GCD curves.

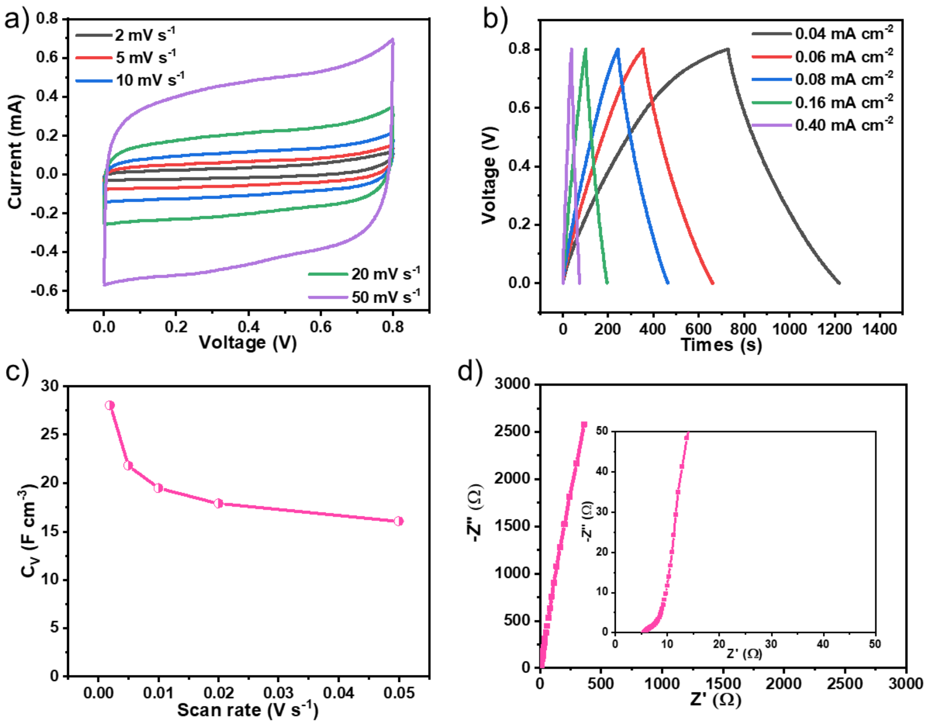

Figure S43. Electrochemical performances of PAE-NiNiPcF$_8$ at PVA/H_2SO_4: a) CV curves at scan rates from 2 to 50 mV s^{-1}; b) GCD curves at current densities of 0.04-0.4 mA cm^{-2}; c) specific volumetric capacitances calculated from CV curves at different scan rates; d) Nyquist plot.

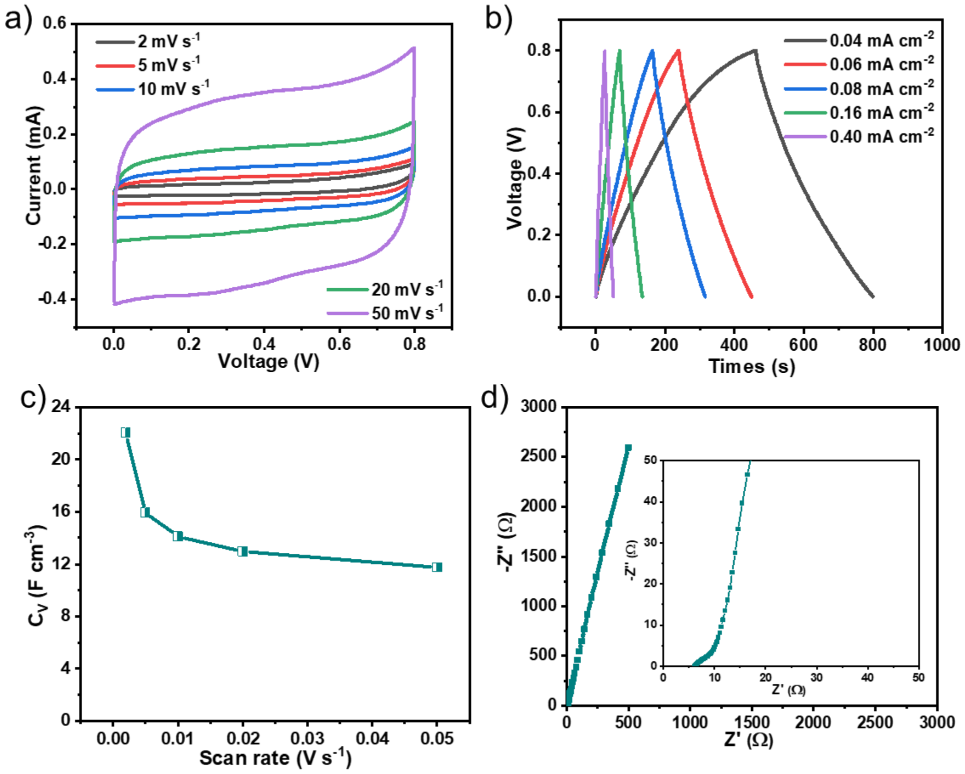

Figure S44. Electrochemical performance of PAE-CuCuPcF$_8$ at PVA/H_2SO_4: a) CV curves at scan rates from 2 to 50 mV s^{-1}; b) GCD curves at current densities of 0.04-0.4 mA cm^{-2}; c) specific volumetric capacitances calculated from CV curves at different scan rates; d) Nyquist plot.

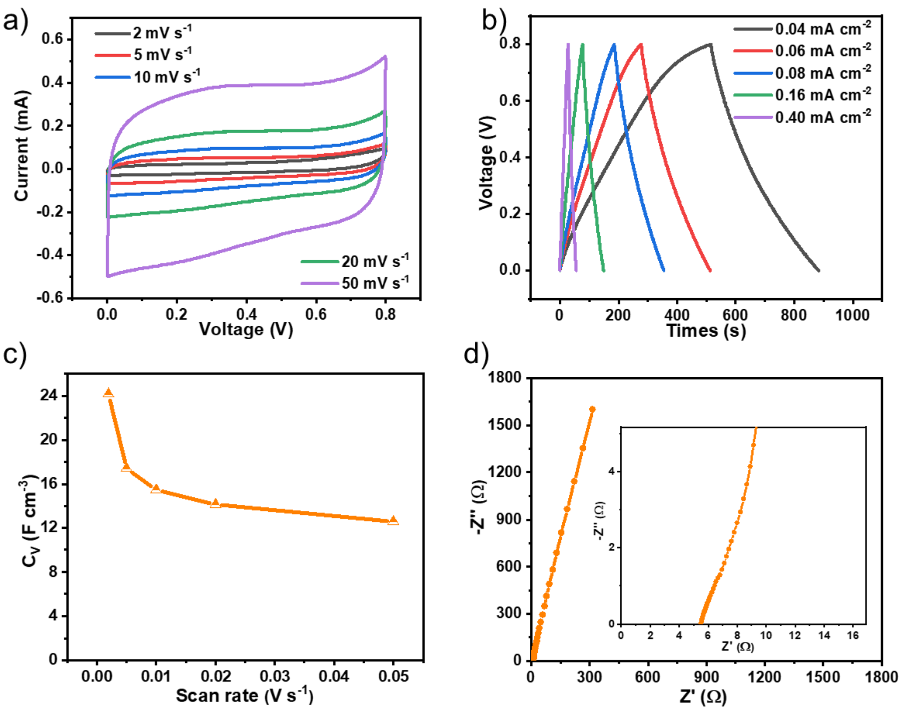

Figure S45. Electrochemical performances of PAE-NiCuPcF$_8$ at PVA/H_2SO_4: a) CV curves at scan rates from 2 to 50 mV s^{-1}; b) GCD curves at current densities of 0.04-0.4 mA cm^{-2}; c) specific volumetrics capacitance calculated from CV curves at different scan rates; d) Nyquist plot.

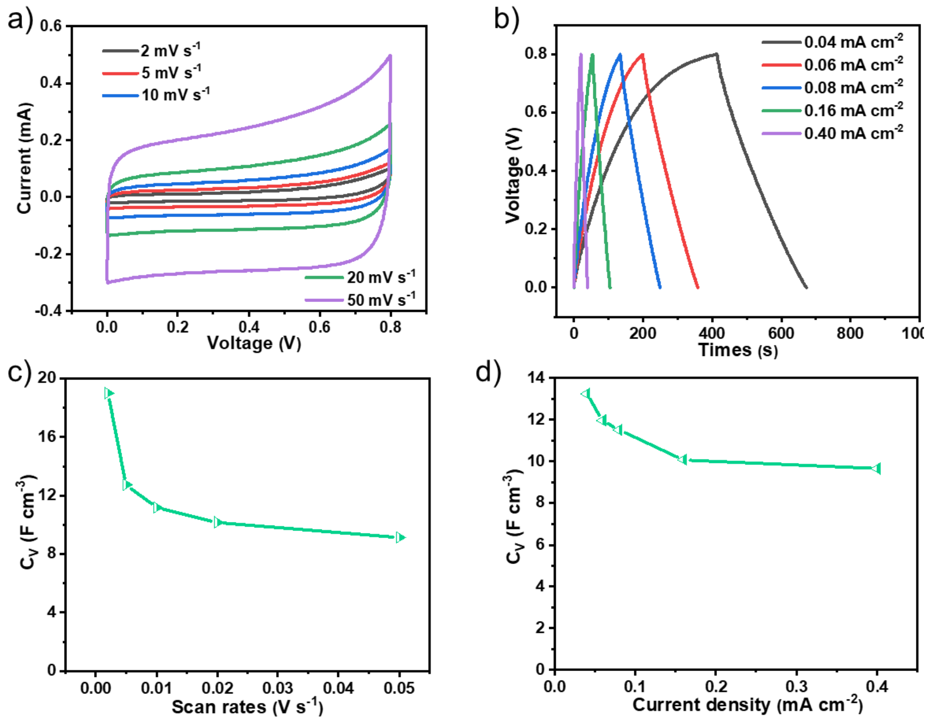

Figure S46. Electrochemical performances of Pure EG at PVA/H_2SO_4: a) CV curves at scan rates from 2 to 50 mV s^{-1}; b) GCD curves at current densities of 0.04-0.4 mA cm^{-2}; c) specific volumetric capacitances calculated from CV curves at different scan rates; d) specific areal capacitance as a function of current densities based on GCD curves.

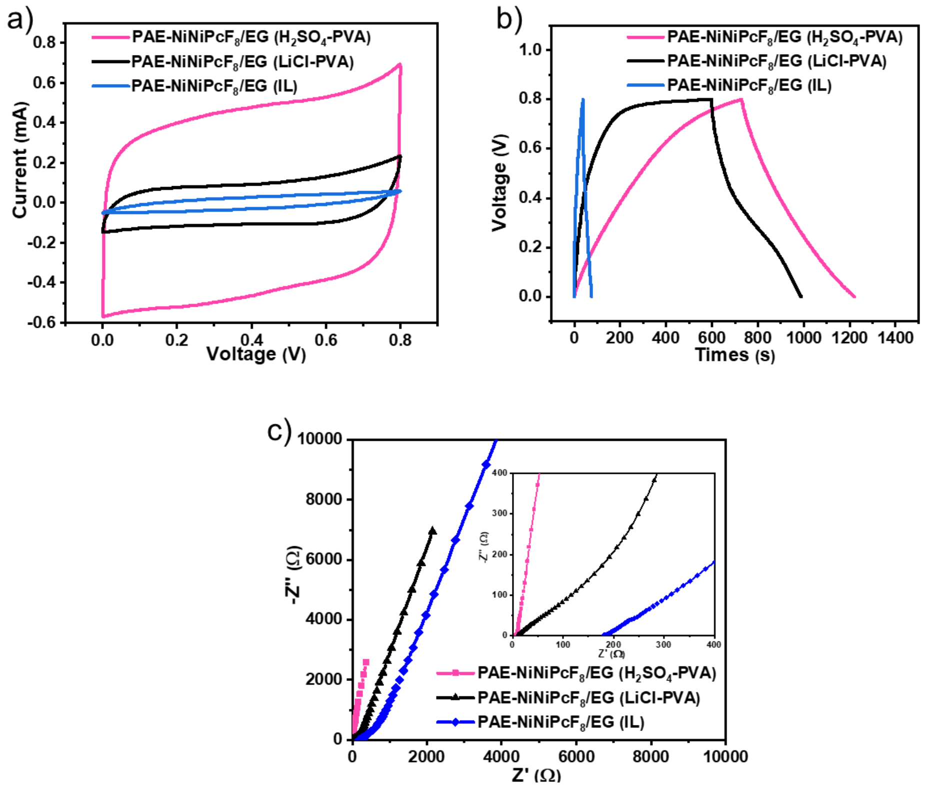

Figure S47. Supercapacitor performance of PAE-NiNiPcF8/EG-5 samples in different electrolytes (H_2SO_4/PVA, LiCl/PVA and IL (1-Butyl-3-Methylimidazolium Tetrafluoroborate), respectively): a) CV curves at 50 mV s^{-1}; b) GCD curves of RTT-MSC-4 at a current density of 0.04 mA cm^{-2}; c) Nyquist plots.

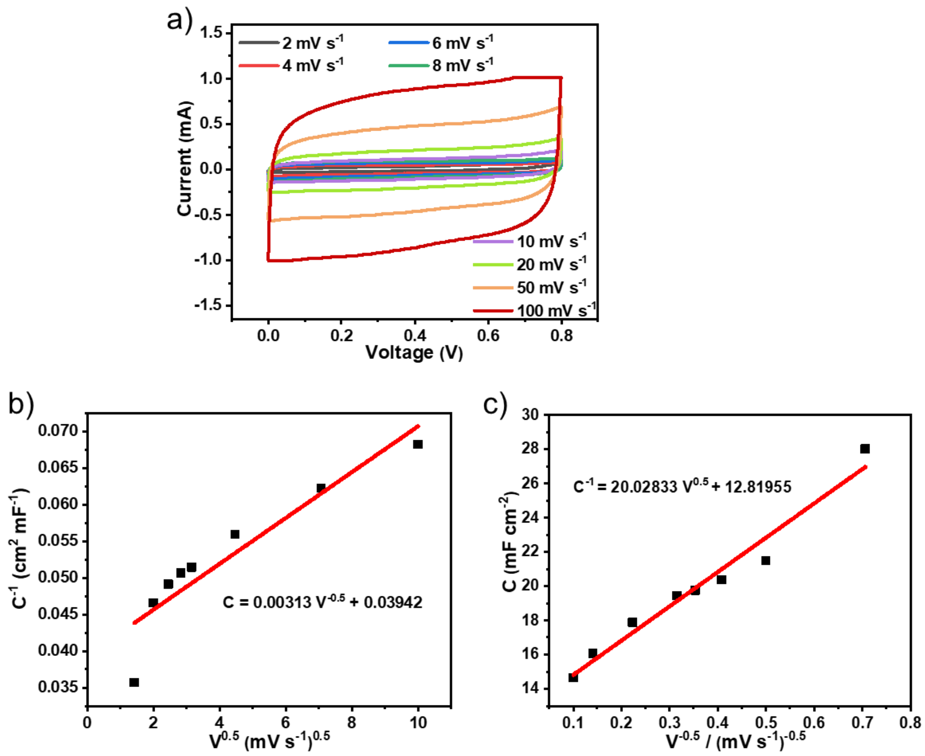

Figure S48. a) CV curves of the PAE-NiNiPcF$_8$/EG-5 MSCs device at different scan rates. Capacitance contribution calculation using Trasatti method: b) plot of reciprocal of areal capacitance (C^{-1}) against the square root of scan rate ($v^{0.5}$); c) plot of C against reciprocal of the square root of scan rate ($v^{-0.5}$).

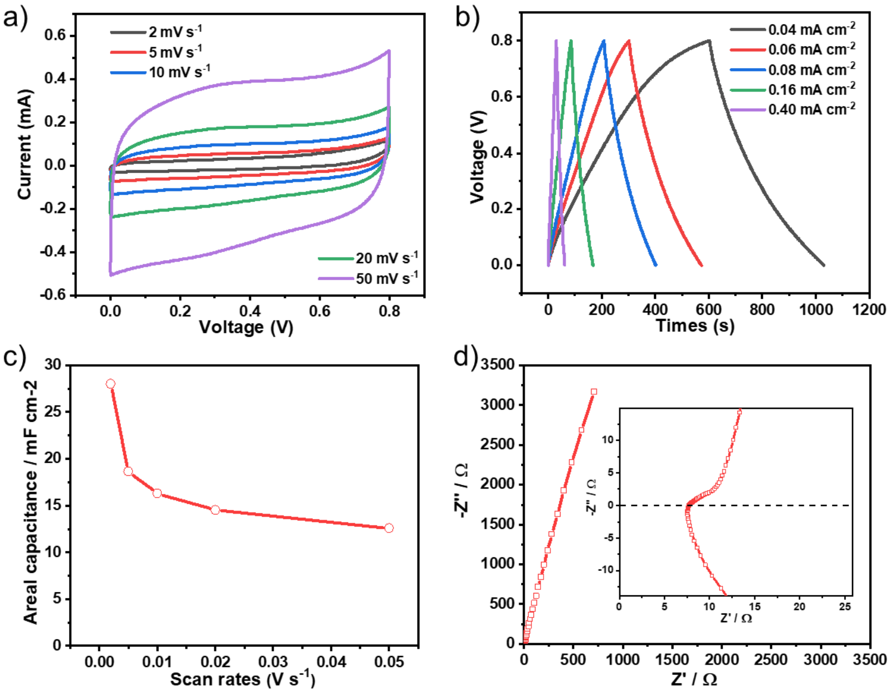

Figure S49. Electrochemical performance of PAE-NiNiPcF$_8$-CST in PVA/H_2SO_4: a) CV curves at the scan rate from 2 to 50 mV s^{-1}; b) GCD curves at current densities of 0.04-0.4 mA cm^{-2}; c) areal capacitance calculated from CV curves at different scan rates; d) Nyquist plot.

S10. Extended PAE-2D COFs based on ZnPc monomer

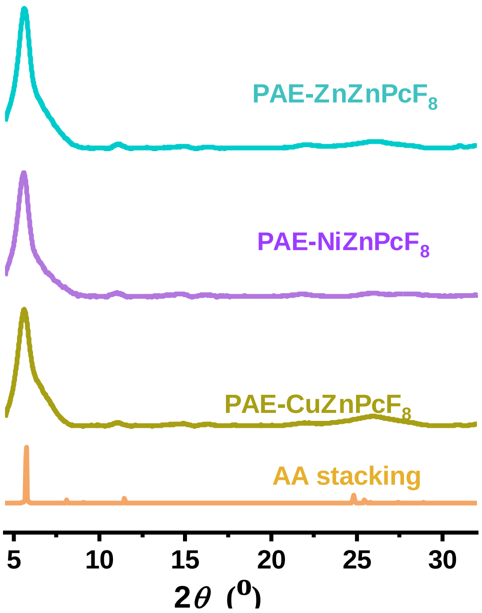

Figure S50. Experimental PXRD patterns of PAE-ZnZnPcF8 (cyan line), PAE-NiZnPcF8 (violet line) and PAE-CuZnPcF8 (brown line); calculated PXRD patterns for AA Stacking (saffron yellow line). The PAE-ZnZnPcF8, PAE-NiZnPcF8 and PAE-CuZnPcF8 have the same morphology and crystalline structure as that of PAE-NiNiPcF8. A series of PXRD peaks at 5.7°, 11.0°, and 26.1° are assigned to (100), (200) and (001) facets, respectively, indicating long-range ordering within the ab-plane with a metal center-to-center distance of 15.4 Å.

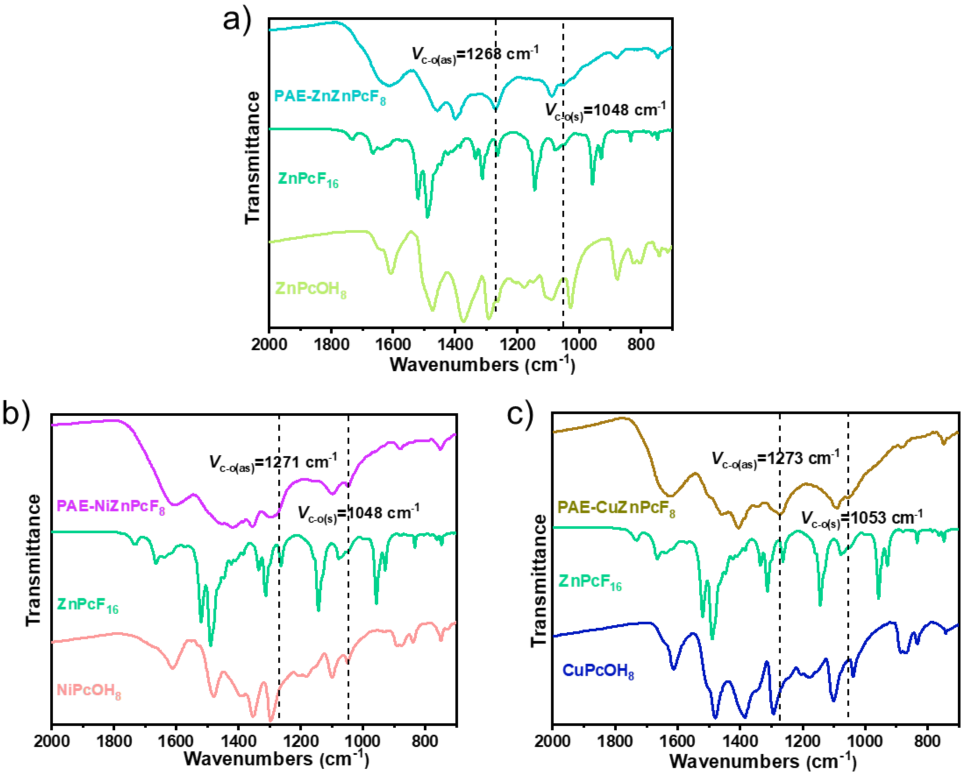

Figure S51. Comparison of PAE-$M_1M_2PcF_8$ and starting materials FT-IR. The vibration bands at ~1270 cm^{-1} and ~1050 cm^{-1} belong to the characteristic peaks of dioxin C-O asymmetric and symmetric stretching modes.

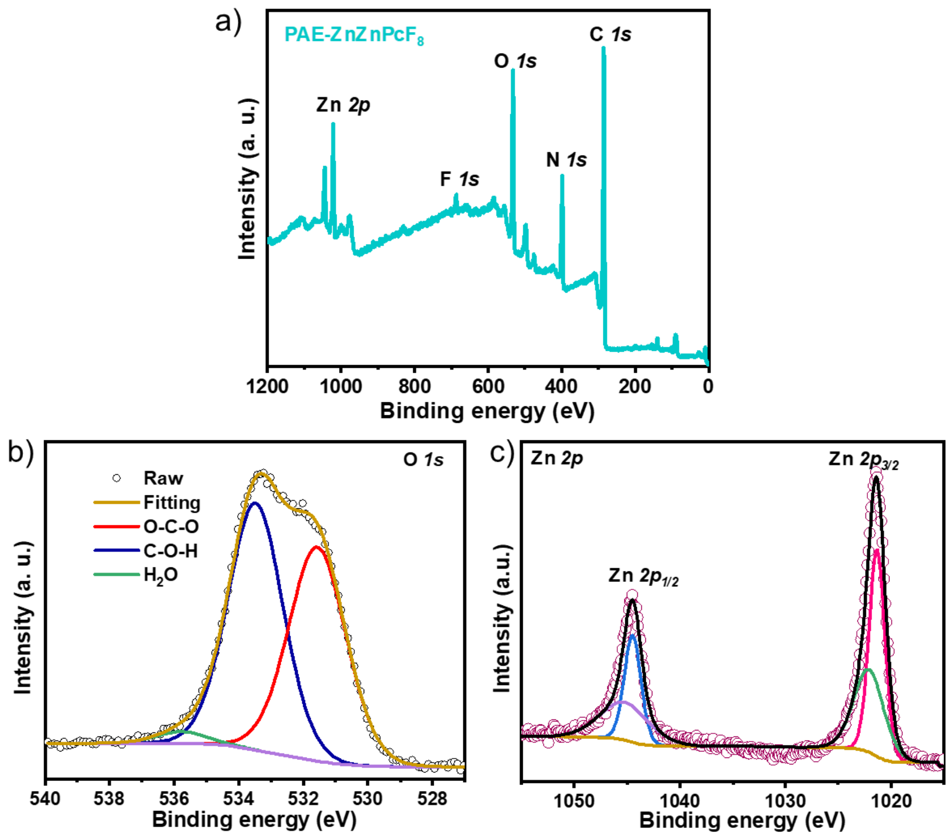

Figure S52. X-ray photoelectron spectroscopy survey spectrum a) and high-resolution spectra for b) O (1s) and c) Zn (2p) of PAE-$ZnZnPcF_8$.

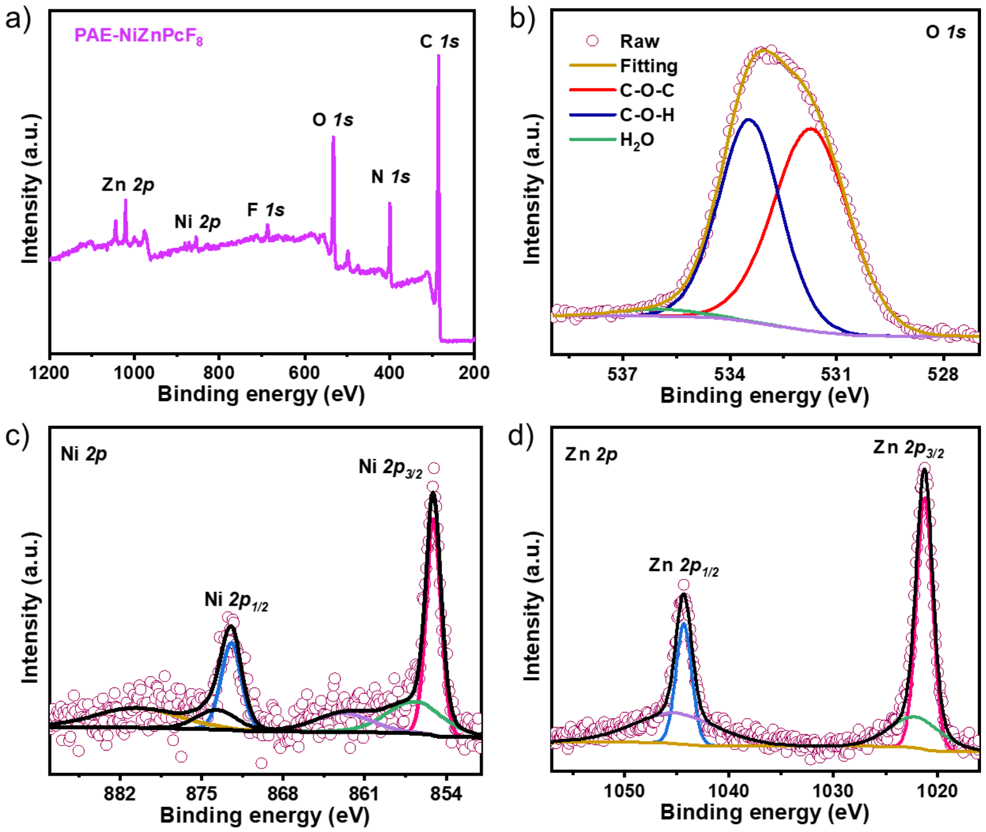

Figure S53. X-ray photoelectron spectroscopy survey spectrum a) and high-resolution spectra for b) O (1s), c) Ni (2p) and d) Zn (2p) of PAE-NiZnPcF$_8$.

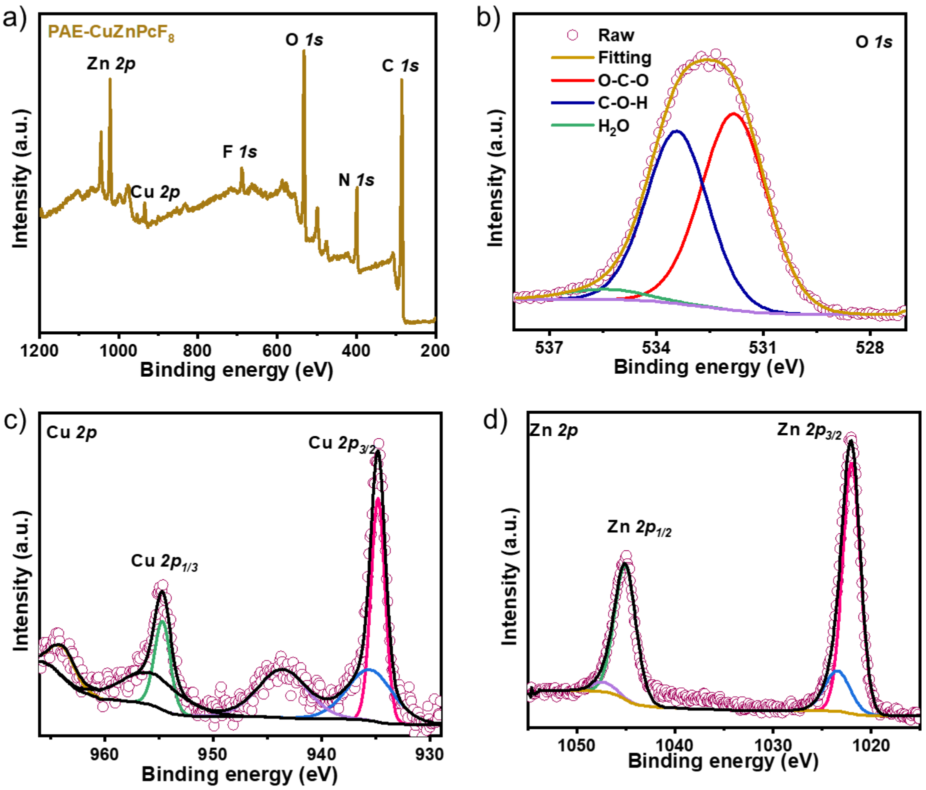

Figure S54. X-ray photoelectron spectroscopy survey spectrum a) and high-resolution spectra for b) O (1s), c) Cu (2p) and d) Zn (2p) of PAE-$CuZnPcF_8$.

The O 1s signals of PAE-$ZnZnPcF_8$, PAE-$NiZnPcF_8$ and PAE-$CuZnPcF_8$ generate peaks at ~531.7, which is attributed to the C-O-C bond of dioxin linkages. The Zn 2p, Ni 2p and Cu 2p XPS spectra exhibit two sets of peaks with binding energies of 1021.37 and 1044.51 eV for Zn $2p_{3/2}$ and Zn $2p_{1/2}$, 854.58 and 872.05 eV for Ni $2p_{3/2}$ and Ni $2p_{1/2}$, 934.79 and 954.77 eV for Cu $2p_{3/2}$ and Cu $2p_{1/2}$, respectively, which suggest stable existence of Zn (II), Ni (II) and Cu (II) species in PAE-$ZnZnPcF_8$, PAE-$NiZnPcF_8$ and PAE-$CuZnPcF_8$.

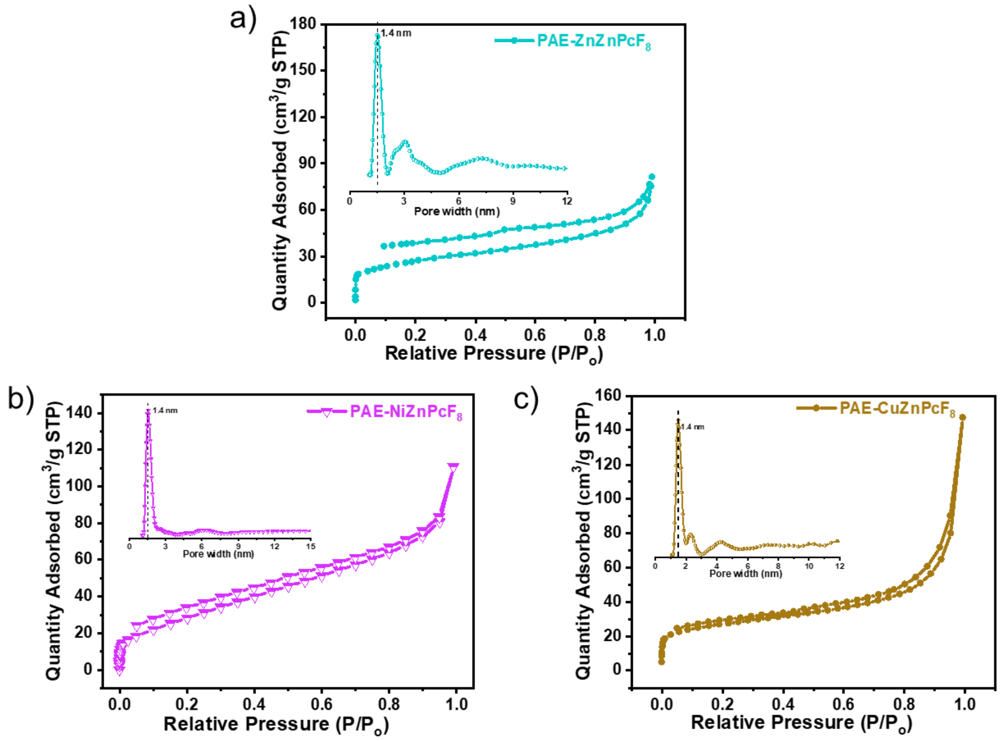

Figure S55. N_2 adsorption-desorption isotherms for a) PAE-ZnZnPcF$_8$, b) PAE-NiZnPcF$_8$, c) PAE-CuZnPcF$_8$. Insets: pore size distributions from fitting the QSDFT model to the adsorption data. Brunauer-Emmett-Teller (BET) surface areas of PAE-ZnZnPcF$_8$, PAE-NiZnPcF$_8$ and PAE-CuZnPcF$_8$ are found to be 70, 91, 96 m^2 g^{-1}, respectively. The pore size distributions of these PAE-2D COFs are calculated using QSDFT model to fit the adsorption branch of the isotherms yielding estimated pore widths of ~1.40 nm, which agree well with the predicted values for an AA stacking model.

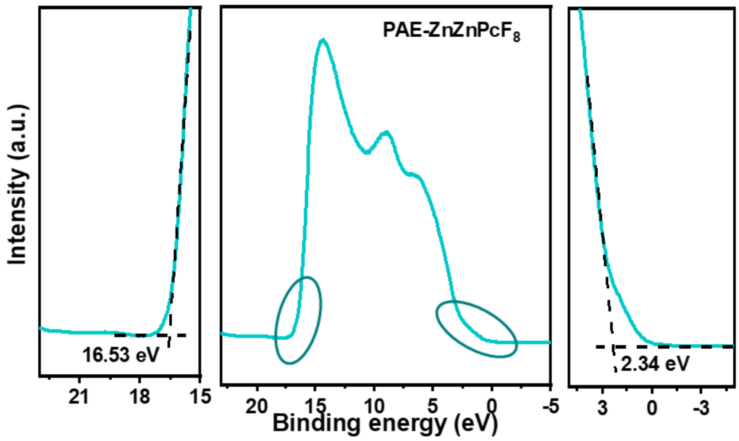

Figure S56. UPS spectrum of PAE-ZnZnPcF$_8$. The E_{vb} (7.03 eV) and work function (4.69) of PAE-ZnZnPcF$_8$ was determined by subtracting the UPS width using excitation photon energy (HeI, 21.22 eV).

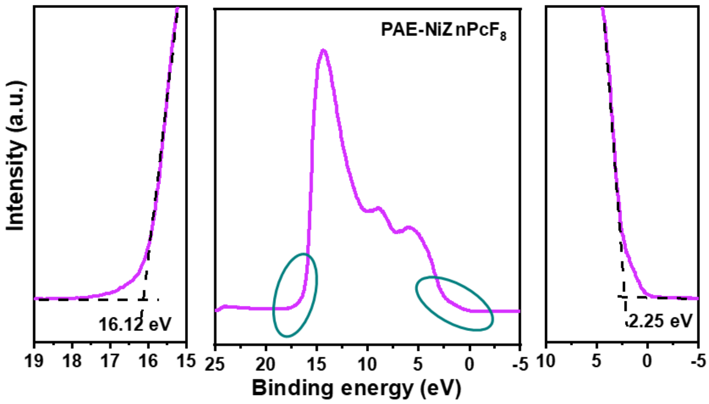

Figure S57. UPS spectrum of PAE-NiZnPcF$_8$. The E_{vb} (7.35 eV) and work function (5.10) of PAE-NiZnPcF$_8$ was determined by subtracting the UPS width using excitation photon energy (HeI, 21.22 eV).

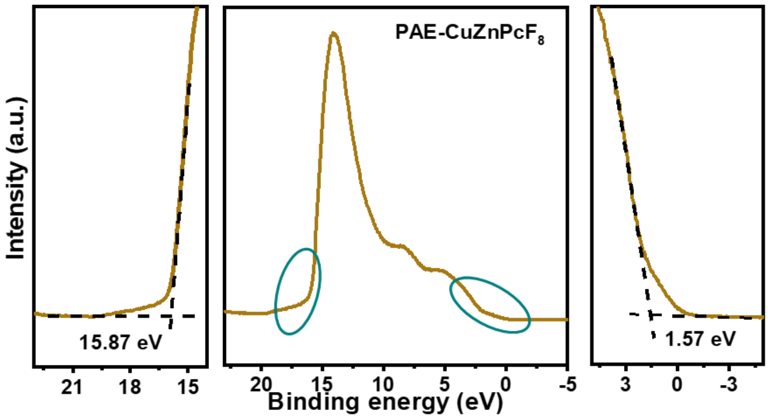

Figure S58. UPS spectrum of PAE-CuZnPcF$_8$. The E_{vb} (6.92 eV) and work function (5.35) of PAE-CuZnPcF$_8$ was determined by subtracting the UPS width using excitation photon energy (HeI, 21.22 eV).

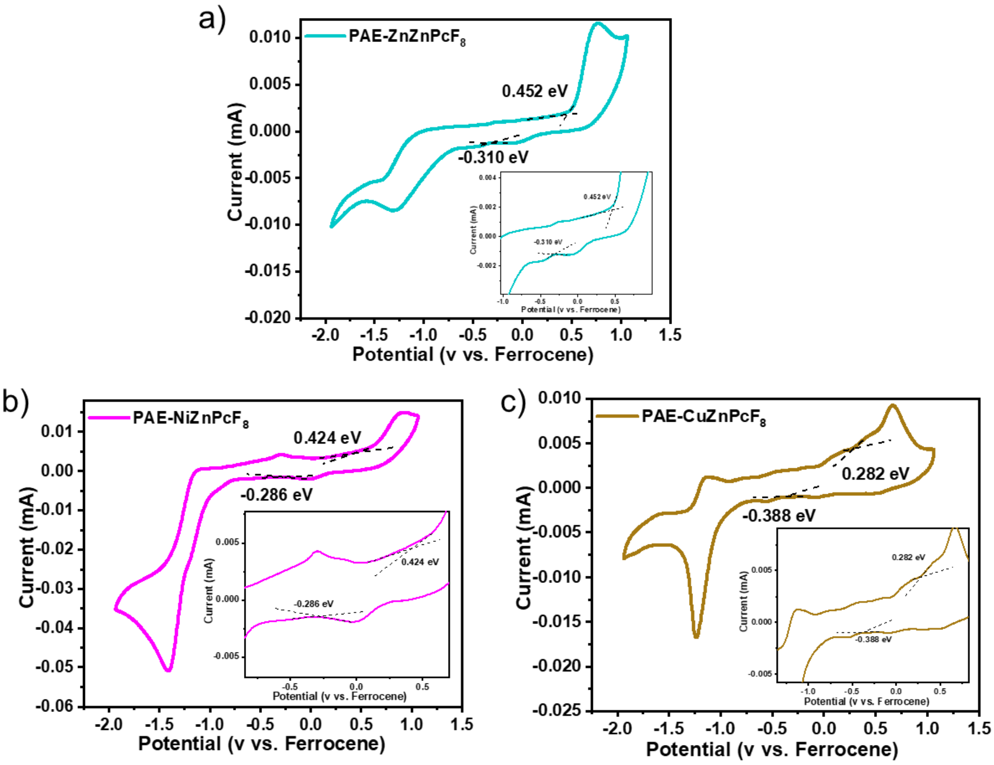

Figure S59. The cyclic voltammograms of a) PAE-ZnZnPcF_8, b) PAE-NiZNPcF_8, c) PAE-CuZnPcF_8 measured in CH_3CN at a scan rate of 20 mV·s^{-1}. Based on the onset reduction and onset oxidation potentials (*vs* Ferroncene), the energy level of valence band (E_{vb}) and electrochemical E_g are determined to be 5.25 and 0.76 eV for PAE-ZnZnPcF_8, 5.22 and 0.71 eV for PAE-NiZnPcF_8, 5.08 and 0.67 eV for PAE-CuZnPcF_8.

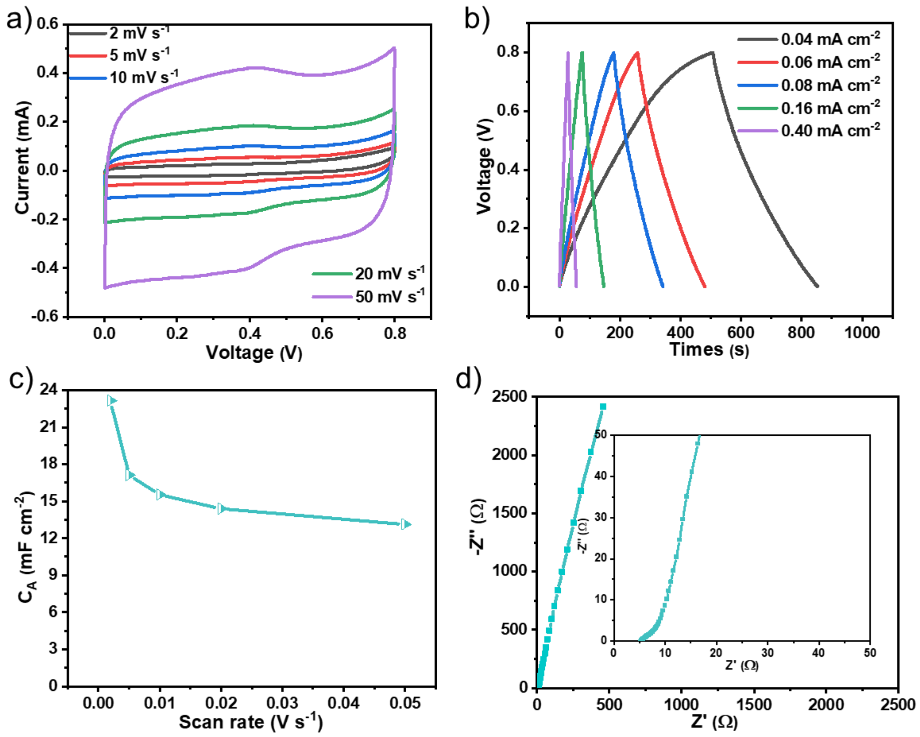

Figure S60. Electrochemical performance of PAE-ZnZnPcF$_8$ at PVA/H$_2$SO$_4$: a) CV curves at scan rates from 2 to 50 mV s^{-1}; b) GCD curves at current densities of 0.04-0.4 mA cm^{-2}; c) specific areal capacitance calculated from CV curves at different scan rates; d) Nyquist plot.

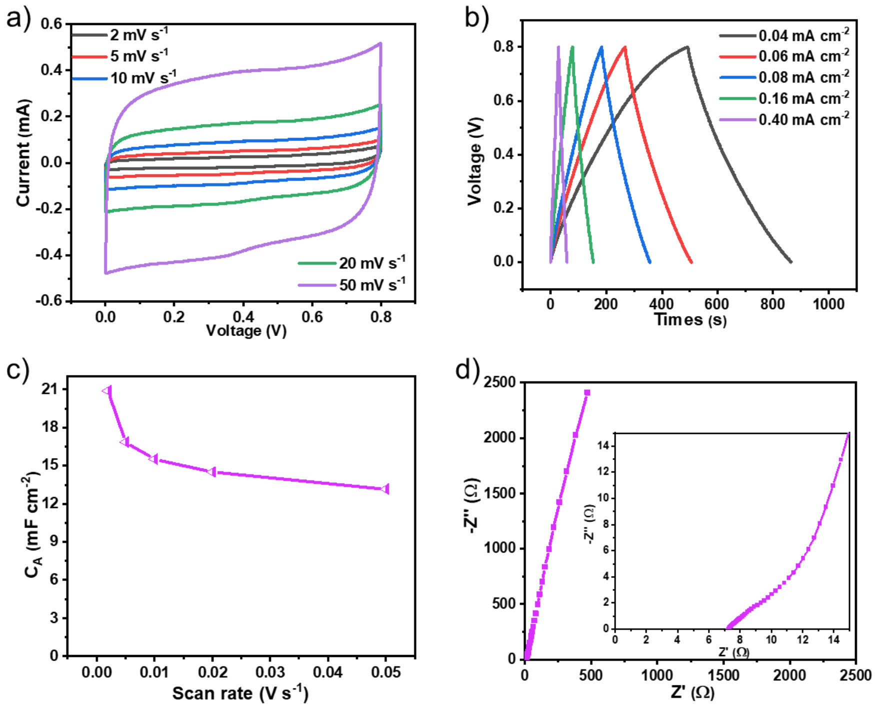

Figure S61. Electrochemical performance of PAE-NiZnPcF$_8$ at PVA/H$_2$SO$_4$: a) CV curves at scan rates from 2 to 50 mV s^{-1}; b) GCD curves at current densities of 0.04-0.4 mA cm^{-2}; c) specific areal capacitance calculated from CV curves at different scan rates; d) Nyquist plot.

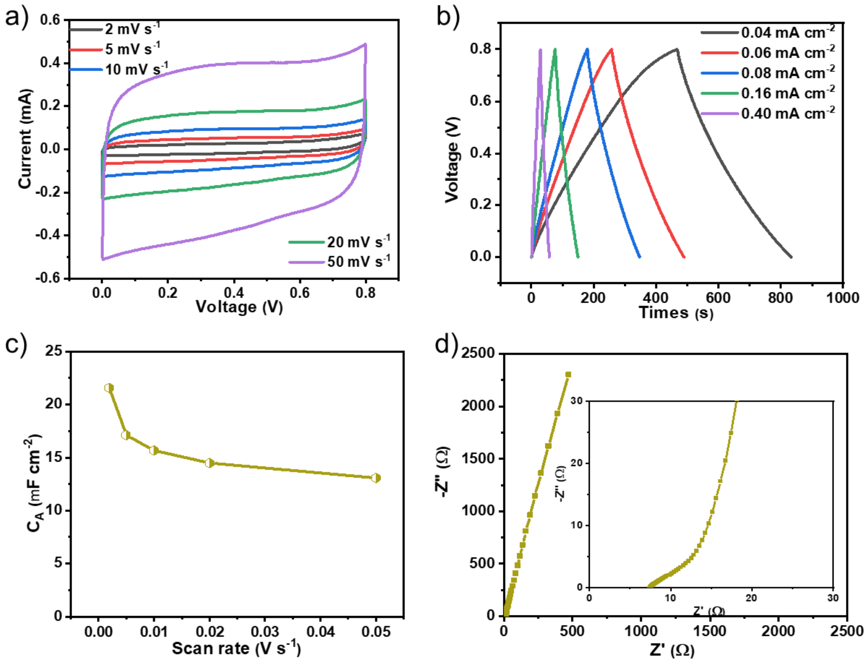

Figure S62. Electrochemical performance of PAE-CuZnPcF$_8$ at PVA/H_2SO_4: a) CV curves at scan rates from 2 to 50 mV s^{-1}; b) GCD curves at current densities of 0.04-0.4 mA cm^{-2}; c) specific areal capacitance calculated from CV curves at different scan rates; d) Nyquist plot.

Table S1. Relative stability of layered PAE-NiNiPcF_8 in different stacking modes.

**Stacking Model**	**AA'**	**AA**	**AB**
**Energy/eV**	-1668.48020679	-1670.07209200	-1667.40326402

Table S2. Nitrogen physisorption data of as-synthesized PAE-M_1M_2PcF_8.

**Sample**	**S_{BET}/m^2g^{-1}**	**S_{Lang}/m^2g^{-1}**	**$S_{V\text{-}t}$ /m^2g^{-1}**	**V_{DFT}/cm^3g^{-1}**	**D_{av}/nm**
**PAE-NiNiPcF_8**	230.401	319.657	31.045	0.106	1.4
**PAE-CuCuPcF_8**	261.285	621.986	66.695	0.331	1.4
**PAE-NiCuPcF_8**	368.758	632.603	72.999	0.212	1.4

Table S3. Elemental contents of as-synthesized PAE-M_1M_2PcF_8 based on XPS results.

**Element (At %)**	**PAE-NiNiPcF_8**	**PAE-CuCuPcF_8**	**PAE-NiCuPcF_8**
**C 1s**	75.80	73.16	74.77
**N 1s**	12.12	15.54	11.94
**O 1S**	10.69	8.81	11.46
**F 1s**	1.08	1.94	1.53
**Ni 2p**	0.31	-	0.18
**Cu 2p**	-	0.54	0.13

Table S4. Electrochemical properties and band gap of as-synthesized PAE-M_1M_2PcF_8.

Sample	E_g^{elec} (ev) [a]	E_{HOMO}^{elec}(ev) [b]	E_{LOMO}^{elec} (ev) [c]	E_g^{opt} (ev)
**PAE-NiNiPcF$_8$**	0.653	-4.810	-4.157	0.91
**PAE-CuCuPcF$_8$**	0.694	-5.044	-4.350	0.91
**PAE-NiCuPcF$_8$**	0.608	-5.020	-4.412	0.85

[a]Band gaps determined from the cyclic voltammogram; [b]Calculated from the corresponding onsets of redox waves referred to Fc/Fc^+ set as -4.8 eV versus vacuum, E_{HOMO}^{CV}= -E_{ox}- 4.8 eV; [c]E_{LOMO}^{CV}= -E_{red}- 4.8 eV.

Table S5. The performance of the in-plane micro-supercapacitors based on different materials.

Name	C_A / mF cm^{-2}	C_V / F cm^{-3}	Electrolyte	Voltage window / V	Energy density maximum	Power density maximum	Ref.
**PAE-NiNiPcF$_8$/EG**	**28.1**	**28.1**	**H_2SO_4/PVA**	**0-0.8**	**2.5 mWh cm^{-3}**	**321 mW cm^{-3}**	**This work**
Ni_2[CuPc(NH)$_8$]-MOF/EG	18.9	31.5	LiCl/PVA	0-0.8	1.7 µWh cm^{-2}	168 mW cm^{-2}	[5]
Ni-CAT MOF/LSG	15.2	-	LiCl/PVA	0-1.4	4.1 µWh cm^{-2}	7 mW cm^{-2}	[6]
g-$C_{34}N_6$ COF /CNT	15.2	-	LiCl/PVA	0-0.8	7.3 mWh cm^{-3}	10.4 W cm^{-3}	[7]
PiCBA-MOF	0.102	34.1	H_2SO_4/PVA	0-1.0	4.7 mWh cm^{-3}	1323 W cm^{-3}	[8]
$Ni(OH)_2$	-	8.8	KOH/PVA	-0.3-0.4	0.59 mWh cm^{-3}	1.8 W cm^{-3}	[9]
rGO/Fe_2O_3	0.347	11.57	KOH/PVA	0-1.0	1.61 mWh cm^{-3}	9.82 W cm^{-3}	[10]
Thiophene/Graphene	3.9	375	H_2SO_4/PVA	0-1.0	13 mWh cm^{-3}	776 W cm^{-3}	[11]
SPANI /Graphene	3.31	16.55	H_2SO_4/PVA	0-0.8	1.5 mWh cm^{-3}	-	[12]
Onion-like carbon	0.9	1.3	1 M TEABF4/PC	0-3.0	10 mWh cm^{-3}	1000 W cm^{-3}	[13]
EG/V_2O_5	3.92	130.7	LiCl/PVA	0-1.0	20 mWh cm^{-3}	235 W cm^{-3}	[14]
TTF-TCNQ/OG	11.6	-	H_2SO_4/PVA	0-1.0	1.57 mWh cm^{-3}	0.2 W cm^{-3}	[15]
MoS_2@rGO/CNT	13.7	-	H_2SO_4/PVA	0-1.0	1.9 µWh cm^{-2}	-	[16]
Phosphorene/Graphene	9.8	37.0	Ionic liquid	0-3.0	11.6 mWh cm^{-3}	1.5 W cm^{-3}	[17]
Laser-Scribed Graphene	2.32	3.05	H_2SO_4/PVA	0-1.0	-	200 W cm^{-3}	[18]
EG/MXene	3.26	33	H_2PO_4/PVA	0-0.5	3.4 mWh cm^{-3}	1.6 W cm^{-3}	[19]
TiO_2/rGO	1.5	233	H_2PO_4/PVA	0-0.8	7.7 mWh cm^{-3}	312 W cm^{-3}	[20]

Reference

[1] T. Iwasaki, A. Fukuoka, X. Min, W. Yokoyama, H. Kuniyasu, N. Kambe, *Org. Lett.* **2016**, *18*, 4868-4871.

[2] a) H. X. Zhong, K. H. Ly, J. C. Zhang, J. Ge, M. C. Wang, Z. Q. Liao, D. Makarov, E. Zschech, E. Brunner, R. H. Dong, X. L. Feng, *Nat. Commun.* **2020**, *11*, 1409; b) C. Yang, R. Dong, M. Wang, P. S. Petkov, Z. Zhang, M. Wang, P. Han, M. Ballabio, S. A. Bräuninger, Z. Liao, J. Zhang, F. Schwotzer, E. Zschech, H.-H. Klauss, E. Cánovas, S. Kaskel, M. Bonn, S. Zhou, T. Heine, X. Feng, *Nat. Commun.* **2019**, *10*, 3260; c) Z. Meng, A. Aykanat, K. A. Mirica, *J. Am. Chem. Soc.* **2019**, *141*, 2046-2053.

[3] a) I. M. Denekamp, F. L. P. Veenstra, P. Jungbacker, G. Rothenberg, *Appl. Organometal Chem.* **2019**, *33*, 4872; b) U. Weiler, T. Mayer, W. Jaegermann, C. Kelting, D. Schlettwein, S. Makarov, D. Wöhrle, *J. Phys. Chem. B* **2004**, *108*, 19398-19403; c) H. Brinkmann, C. Kelting, S. Makarov, O. Tsaryova, G. Schnurpfeil, D. Wöhrle, D. Schlettwein, *Phys. Stat. Sol. (a)* **2008**, *205*, 409-420; d) S. K. Das, A. Mahler, A. K. Wilson, F. D'Souza, *ChemPhysChem* **2014**, *15*, 2462-2472; e) T. V. Basova, N. S. Mikhaleva, A. K. Hassan, V. G. Kiselev, *Sensor Actuat. B: Chem.* **2016**, *227*, 634-642; f) L. Wang, S. Song, F. Ma, G. Jiang, *Dig. J. Nanomater. Bios.* **2018**, *13*, 505-509.

[4] K. Parvez, Z.-S. Wu, R. Li, X. Liu, R. Graf, X. Feng, K. Müllen, *J. Am. Chem. Soc.* **2014**, *136*, 6083-6091.

[5] M. Wang, H. Shi, P. Zhang, Z. Liao, M. Wang, H. Zhong, F. Schwotzer, A. S. Nia, E. Zschech, S. Zhou, S. Kaskel, R. Dong, X. Feng, *Adv. Funct. Mater.* **2020**, *30*, 2002664.

[6] H. Wu, W. Zhang, S. Kandambeth, O. Shekhah, M. Eddaoudi, H. N. Alshareef, *Adv. Energy Mater.* **2019**, *9*, 1900482.

[7] J. Xu, Y. He, S. Bi, M. Wang, P. Yang, D. Wu, J. Wang, F. Zhang, *Angew.Chem. Int. Ed.* **2019**, *58*, 12065-12069.

[8] C. Yang, K. S. Schellhammer, F. Ortmann, S. Sun, R. Dong, M. Karakus, Z. Mics, M. Loffler, F. Zhang, X. Zhuang, E. Canovas, G. Cuniberti, M. Bonn, X. Feng, *Angew Chem Int Ed.* **2017**, *56*, 3920-3924.

[9] H. Wu, K. Jiang, S. Gu, H. Yang, Z. Lou, D. Chen, G. Shen, *Nano Research* **2015**, *8*, 3544-3552.

[10] S. Gu, Z. Lou, L. Li, Z. Chen, X. Ma, G. Shen, *Nano Research* **2016**, *9*, 424-434.

[11] Z.-S. Wu, Y. Zheng, S. Zheng, S. Wang, C. Sun, K. Parvez, T. Ikeda, X. Bao, K. Müllen, X. Feng, *Adv. Mater.* **2017**, *29*, 1602960.

[12] B. Song, L. Li, Z. Lin, Z.-K. Wu, K.-s. Moon, C.-P. Wong, *Nano Energy* **2015**, *16*, 470-478.

[13] D. Pech, M. Brunet, H. Durou, P. Huang, V. Mochalin, Y. Gogotsi, P.-L. Taberna, P. Simon, *Nat. Nanotechnol.* **2010**, *5*, 651-654.

[14] P. Zhang, F. Zhu, F. Wang, J. Wang, R. Dong, X. Zhuang, O. G. Schmidt, X. Feng, *Adv. Mater.* **2017**, *29*, 1604491.

[15] D. Zhao, W. Chang, C. Lu, C. Yang, K. Jiang, X. Chang, H. Lin, F. Zhang, S. Han, Z. Hou, X. Zhuang, *Small* **2019**, *15*, 1901494.

[16] W. Yang, L. He, X. Tian, M. Yan, H. Yuan, X. Liao, J. Meng, Z. Hao, L. Mai, *Small* **2017**, *13*, 1700639.

[17] H. Xiao, Z.-S. Wu, L. Chen, F. Zhou, S. Zheng, W. Ren, H.-M. Cheng, X. Bao, *ACS Nano* **2017**, *11*, 7284-7292.

[18] M. F. El-Kady, R. B. Kaner, *Nat. Commun.* **2013**, *4*, 1475.

[19] H. Li, Y. Hou, F. Wang, M. R. Lohe, X. Zhuang, L. Niu, X. Feng, *Adv. Energy Mater.* **2017**, *7*, 1601847.

[20] S. Wang, Z.-S. Wu, S. Zheng, F. Zhou, C. Sun, H.-M. Cheng, X. Bao, *ACS Nano* **2017**, *11*, 4283-4291.
